# The effectiveness of protein supplements on athletic performance and post-exercise recovery − a Bayesian multilevel meta-analysis of randomized controlled trials

**DOI:** 10.1080/15502783.2025.2605338

**Published:** 2025-12-23

**Authors:** Shiao Zhao, Xiaoyu Zhang, Taihe Liang, Sanfan Ng, Yiran Liu, Ziheng Ning

**Affiliations:** a Macao Polytechnic University, Faculty of Health Sciences and Sports, Macao, People's Republic of China

**Keywords:** Protein supplements, athletic performance, post-exercise recovery, endurance performance, muscle strength

## Abstract

**Background:**

Protein supplements are a popular category of dietary supplements among fitness enthusiasts and athletes. However, research providing definitive conclusions on the effects of protein on athletic performance and post-exercise recovery remains limited. Key factors, such as protein source, timing, and optimal dosage, require further investigation to clarify their impact.

**Method:**

A systematic search across seven databases identified 6,129 studies, which were screened using the Covidence online tool. After independent selection, data extraction, and risk of bias assessment by two reviewers, 75 studies involving 1,206 athletes were included in the meta-analysis. A multilevel meta-analysis synthesized data from the included studies using a Bayesian hierarchical model with the brms package. Publication bias was assessed using a funnel plot generated with the PublicationBias package and by calculating the P value of Egger's test through the metafor package. Additionally, a moderation analysis with the brms package was conducted to examine the relationship between seven moderators and effect sizes.

**Results:**

The results demonstrated that the effects of protein-carbohydrate supplements showed statistical significance in comparison to the placebo group [μ(SMD): 0.57, 95% CI: 0.2 to 0.93] in enhancing endurance performance. Pure protein supplements demonstrated statistically significant effects compared to the placebo group in both endurance performance [μ(SMD): 0.37, 95% CI: 0.02 to 0.71] and muscle strength [μ(SMD): 0.72, 95% CI: 0.18 to 1.27]. For post-exercise recovery, pure protein supplements also showed statistically significant effects compared to carbohydrate supplements for maintaining glycogen resynthesis [μ(SMD): 0.83, 95% CI: 0.21 to 1.46]. However, the results indicated that all significant effects were observed in randomized controlled trials where the energy intake between the intervention and control groups was not matched.

**Conclusion:**

The effects of protein supplementation on athletic performance and post-exercise recovery appear to be limited. Protein supplements showed beneficial effects compared to no supplementation. However, all statistically significant results were derived from studies in which energy intake was not matched between groups. This suggests that the observed benefits may not be attributable to protein per se. An additional intake of 1 g/kg/day of protein from supplements, resulting in a total daily protein intake of approximately 2 g/kg/day, appears to be most effective for enhancing athletic performance.

**Registration:**

Registered at the International Prospective Register of Systematic Reviews (PROSPERO) (identification code CRD42024608194).

## 1 Background

Protein, a key macronutrient, is essential for athletes due to its role in growth, tissue repair, and metabolic and hormonal regulation [1]. The National Strength and Conditioning Association and the American College of Sports Medicine have established the recommended daily protein intake for endurance athletes (1.2–1.4 g/kg) and strength athletes (1.6–1.7 g/kg) [1–3]. This study aimed to investigate whether different types and doses of protein supplements are associated with improvements in athletic performance and post-exercise recovery.

Currently, the effectiveness of protein supplements on sports performance and post-exercise recovery remains unclear and contentious [4–7]. Research findings are inconsistent and often lack credibility due to small sample sizes. Some studies suggest that the beneficial effects of protein intake on performance, such as endurance and muscle strength, are relatively limited [8,9]. Multiple studies have shown that adding protein to carbohydrate does not improve endurance performance, particularly in cycling. Moreover, when energy intake is controlled and carbohydrate is ingested at a rate considered optimal for exogenous carbohydrate oxidation, the addition of protein provides no further enhancement in endurance performance [10–13]. Additionally, Jäger et al. found that the effect of protein on muscle strength is minimal, with many studies reporting non-significant outcomes [8].

Despite evidence questioning the efficacy of protein supplements, other studies have shown significant benefits for athletes [4,6,14–30]. Performance improvements in endurance exercise by protein supplements that lack statistical significance may still be therapeutically important, particularly for athletes [28,31]. In the Olympics, a one-second difference can determine the champion. A study involving 30 clinically healthy athletes found that protein supplements enhance aerobic energy provision, leading to improved endurance performance [15]. On the other hand, the latest review concluded that protein supplementation is an effective strategy for enhancing lower-body mass and strength [29,32].

Furthermore, few studies or reviews have systematically examined how factors such as protein type, timing, dose, and athlete characteristics (e.g. gender and age) influence outcomes. Recent research comparing plant protein to animal protein has been increasing, but most studies have not found a significant advantage of plant protein over animal protein in enhancing athletic performance, including endurance and muscle strength [21,33–35]. Additionally, pre-sleep protein ingestion may be an effective strategy to enhance overnight muscle protein synthesis. When used during extended resistance-type exercise, pre-sleep protein supplementation can significantly increase muscle mass and strength [36–38]. However, no comprehensive study has yet examined the effects of protein type and timing on athletes. The use of varying protein types and doses across different studies has led to inconsistent outcomes. Therefore, there is a need for large-scale studies that provide effective recommendations for protein supplement use in athletes.

This study aimed to evaluate the efficacy of protein supplements on athletic performance and post-exercise recovery in athletes using Bayesian multilevel meta-analysis. We also explored the relationship between various protein supplementation strategies, including protein supplements alone, protein co-ingested with carbohydrates, differing protein sources, protein timing, additional protein doses, and the total daily protein intake, which includes the protein content in three daily meals.

Based on existing but limited research, we proposed three hypotheses: (1) both protein-carbohydrate supplements and high-protein supplements can effectively improve athletic performance and post-exercise recovery; (2) whey protein and soy protein are the optimal protein types; (3) the effectiveness of protein supplementation may vary depending on factors such as protein timing, the distinction between acute and chronic interventions, and whether the energy intake is matched between the experimental and control groups.

## 2 Method

This study was registered with PROSPERO (CRD42024608194) and adhered to the PRISMA reporting guidelines [39]. A Bayesian meta-analysis was performed in conjunction with a systematic review using Covidence, GRADEprofiler, R version 4.4.1, and GetData Graph Digitiser.

### 2.1 Literature search

A comprehensive search strategy was developed using Medical Subject Headings (MeSH) and free-text search terms to systematically search the SPORTDiscus, PubMed, Ovid, Web of Science, MEDLINE, CINAHL, and Scopus databases on September 26, 2024. The keywords and subject headings were finalised through discussion between the two authors (SZ & XZ). Detailed search strings for each database are provided in Supplementary File S1. A total of 6,129 studies were retrieved using the Covidence online tool for systematic review.

### 2.2 Inclusion and exclusion criteria

Following the PICOS principle, non-human studies and non-comparative studies were excluded. Eligible studies were randomised controlled trials (RCTs) that included protein supplements. The studies without a control group or protein group were not considered. Non-original studies (e.g. letters, reviews, or editorials) and studies lacking available data for extraction were also excluded.

Participants in RCTs must be athletes. Studies were only included if the experimental group received a protein supplement and the control group did not receive any form of protein supplementation (e.g. placebo, carbohydrate, or no supplement). Trials comparing different types, timing, or doses of protein supplements without a non-protein control group were excluded to ensure the presence of a proper comparison group. Outcomes in studies were required to be related to athletic performance, including endurance performance and muscle strength, or post-exercise recovery, encompassing glycogen resynthesis and fatigue.

### 2.3 Selection process

The automatic tool in Covidence recommended by PRISMA was used to generate the flow diagram [39]. Two reviewers (SZ & XZ) independently screened titles and abstracts, followed by full texts against eligibility criteria using the Covidence online tool.

### 2.4 Risk of bias assessment

The risk of bias for all included studies was independently assessed using the guidelines and criteria outlined in the Cochrane Handbook for Systematic Reviews of Interventions. Two authors (SZ & XZ) assessed the included studies through the Cochrane risk of bias (RoB) criteria in RCTs within Covidence. Seven areas of bias were evaluated: (1) random sequence generation; (2) allocation concealment; (3) blinding of participants and personnel; (4) blinding of outcome assessment; (5) incomplete outcome data; (6) selective reporting; and (7) other bias. The risk of bias was classified as low, unclear, or high. After independent assessments, the authors reached a consensus through discussion. Final results were recorded in an Excel template and input into R software to create risk of bias summary plots using the robvis package [40]. Studies with more than two and fewer than four areas marked as unclear risk were classified as moderate risk overall.

### 2.5 Publication bias

To assess publication bias, we used the PublicationBias package in R [41,42], which provides a sensitivity analysis approach that does not rely on funnel plot symmetry. Instead, it estimates how strong publication bias would need to be to reduce the observed effect size or its confidence interval to a specified threshold.

Assuming that statistically significant and positive results were more likely to be published (favor_positive = TRUE), we created a significance-based funnel plot to visually compare affirmative (significant positive) and non-affirmative (non-significant or negative) studies. The worst-case estimate, based on non-affirmative studies only, provides a benchmark for evaluating robustness. We also calculated the s-value, defined as the minimum selection ratio (*η*) required to attenuate the effect to the null. An s-value of “not possible” indicates that no plausible publication bias could fully explain the observed effect.

To supplement this newer method, we also applied traditional techniques using the metafor package. We generated a standard funnel plot, conducted Egger's test, and applied the trim-and-fill method when Egger's test yielded *p* < 0.05, providing additional evidence from a Frequentist perspective.

### 2.6 Data extraction

Data were extracted independently by two authors (SZ & XZ) using Covidence, with conflicts resolved through discussion with the third author (ZN). For each study, characteristics including intervention group, control group, first author, publication year, study design, country, participants' ages, body mass index (BMI), athlete type, protein type, extra protein dose, overall protein dose, duration, washout or follow-up period, and outcome measure were extracted. The outcomes included time to exhaustion, lower body strength, upper body strength, one-repetition maximum (1 RM), cycling time trials, maximum voluntary contraction (MVC), counter-movement jump (CMJ), anaerobic peak and average power, vertical jump, cycling distance, hand grip strength, maximum speed, average speed, blood glucose, muscle glycogen, blood lactate, muscle soreness, physical and mental fatigue, and VO_2_max.

Data were presented as mean ± standard deviation (M ± SD). When data were not presented as exact numbers, GetData Graph Digitiser [43] was used to extract data from graphs. We used an online tool called Meta-analysis Accelerator to convert data that was not initially in M ± SD format and to calculate the change values (mean and standard deviation) accounting for pre- and post-intervention baseline measurements [44]. Since none of the studies provided correlation coefficients, a correlation of 0.5 was assumed for all trials, following the recommendation of Follmann et al [45].

The Metafor package in R was used to calculate the standardised mean difference (SMD) according to the formula:

$$\mathrm{SMD}=\sqrt{\frac{(\mathrm{n1i}-1)*{\mathrm{sd1i}}^{2}+{(\mathrm{n2i}-1)*\mathrm{sd2i}}^{2}}{\mathrm{n1i}+\mathrm{n2i}-2}}$$

### 2.7 Summary measures and synthesis

Bayesian mixed-effects models implemented in the R package brms [46] were used to analyse variation in effect sizes. We fitted the models assuming a normal distribution and included random effects for within-study ID and between-study ID to account for within-study and between-study heterogeneity in each result according to two formulas:

$$\begin{array}{l} I_{\mathrm{within}}^{2}=\frac{\tau_{\mathrm{withID}}^{2}}{\mathrm{total}\_\mathrm{variance}}\times 100\\ I_{\mathrm{between}}^{2}=\frac{\tau_{\mathrm{between}ID}^{2}}{\mathrm{total}\_\mathrm{variance}}\times 100 \end{array}$$

In addition to heterogeneity indicators, we tested two indices through the metainc package [47] including the dissimilarity index (DI) and the across-studies inconsistency (ASI) to assess the potential inconsistency in each model. These indices were considered more suitable for Bayesian meta-analysis compared to traditional heterogeneity indicators such as I^2^ or Q-test [48]. A DI ≥ 50% and an ASI ≥ 25% were considered indicative of important inconsistency. These indices differ from previously existing measures by considering effect size (ES) in the context of decision thresholds (DTs). We set three decision thresholds as suggested for SMD, namely 0.2, 0.5, and 0.8 [48].

Weakly informative priors (mean = 2, sd = 0.5) were used for the random effects. While not universally optimal, weakly informative priors are widely regarded as a best-practice default in Bayesian meta-analysis, especially when prior knowledge is limited [49]. Athletic performance and post-exercise recovery were analysed separately, with each data point categorised by protein supplementation strategy (PRO vs. PRO-CHO), protein source, additional protein dose, total protein intake, energy matching, fasted state, study design (parallel or crossover), acute or chronic ingestion and protein timing. Secondly, to explore the sources of heterogeneity, we further categorised the outcome measures by establishing muscle model, endurance model, glycogen model, and fatigue model. We then applied interaction models using the brms package to analyse the data. This approach helped reduce data complexity and mitigate heterogeneity.

Fifteen models were fitted for each of the athletic performance and post-exercise recovery datasets. Ten Bayesian models (Fasted model, Energy model, Design model, Acute-chronic model, Endurance model, Muscle model, Glycogen model, Fatigue model and Interaction model) used in this study were established post hoc. Model specifications were refined based on reviewer feedback and adjustments made during the analytical process to address initial errors and improve model fit. Details of the fitted models are provided below:

(1) Null model to estimate overall effect sizes. We fitted separate models on the full dataset for each athletic performance and post-exercise recovery.
(2) Supplements model (PRO vs. PLA, PRO vs. CHO, PRO-CHO vs. PLA, and PRO-CHO vs. CHO).
(3) Protein source model (whey, wheat, soy, milk, hydrolysed collagen, egg white, combined protein source, casein, beef, and BCAA protein).
(4) Protein dose model (0–1 g/kg, 1–2 g/kg and 2–3 g/kg). Protein dose model means the amount of daily protein intake in extra protein supplements. The protein intake from three meals a day was excluded.
(5) Overall dose model (0–1 g/kg, 1–2 g/kg and 2–3 g/kg). Overall dose model means daily overall protein intake.
(6) Protein timing model (day and night). Protein timing model refers to the timing of protein supplement ingestion.
(7) Fasted model (fasted or fed). The fasted model refers to whether athletes consumed any additional food before taking the supplement.
(8) Energy model (matched or unmatched). The energy model refers to whether the daily calorie intake was matched between the experimental and control groups.
(9) Design model (parallel or crossover). The design model refers to whether the study design was a randomised controlled trial or a randomised crossover trial.
(10) Acute-Chronic Model. The acute-chronic model refers to the exploration of the effectiveness of acute versus long-term protein intake on muscle strength and endurance.
(11) Endurance model. The endurance model refers to further categorising endurance outcomes to explore the effective sources and heterogeneity of protein supplementation effects.
(12) Muscle model. The muscle model refers to further categorising muscle strength outcomes to explore the effective sources and heterogeneity of protein supplementation effects.
(13) Glycogen model (glucose and muscle glycogen). The glycogen model refers to further categorising glycogen resynthesis outcomes to explore the effective sources of protein supplementation effects. Glycogen resynthesis is a biphasic process involving an initial rapid phase and a slower phase. During the rapid phase, increased muscle membrane permeability and glycogen synthase activity enhance glucose uptake from blood, facilitating the restoration of muscle glycogen. Carbohydrate ingestion elevates blood glucose and insulin levels, further accelerating glycogen synthesis, highlighting the pivotal role of blood glucose as a substrate for muscle glycogen replenishment [50].
(14) Fatigue model (fatigue index, blood lactate and subjective rating). The fatigue model refers to further categorising fatigue outcomes to explore the effective sources of protein supplementation effects.
(15) Interaction model (Performance × Energy, Performance × Supplements, or Performance × Timing). The interaction model refers to examining how performance outcomes differ under various conditions of protein supplementation, such as supplement type, timing of intake, and energy matching, with the main goal of exploring sources of heterogeneity and the effectiveness of protein supplements.

Four chains of 10,000 iterations were applied for each model. To assess Markov chain convergence in this study, we used the Rhat statistic as the sole diagnostic metric. The Rhat value compares between-chain and within-chain variances, with values near 1 indicating adequate convergence of the chains. The degree of support for a difference from zero was assessed using Bayes Factors (BFs). The function bayesfactor_parameters from the bayestestR package was used to implement the reciprocal of the Savage-Dickey density ratio, which was used to estimate BFs [51]. Bayes Factors were interpreted on a conventional scale, with values exceeding 3 indicating moderate evidence and those above 10 reflecting strong support for the presence of an effect [52]. For complex hierarchical models, computing the 95% highest-density interval (HDI), which represents the most plausible outcomes in the posterior distribution, is seamless, as opposed to approximating *p*-values and confidence intervals, which involve additional assumptions [53].

### 2.8 Certainty in evidence

The quality of the protein evidence was assessed using the GRADE approach, which evaluates the risk of bias, inconsistency, indirectness, and imprecision of effect estimates. The GRADE approach classifies the quality of evidence as high, moderate, low, or very low. Furthermore, in the evaluation of inconsistency, in addition to the heterogeneity indicator (I^2^), we also used the previously calculated inconsistency indicator (DI & ASI) to help judge the quality of the results. If the inconsistency indicator showed moderate or above inconsistency, the level of the results was downgraded by at least one level.

### 2.9 Moderation analysis

In this study, a moderation analysis was conducted using the brms package in R, incorporating four trait moderator variables: age, weight, percentage of women among participants, and sample size. Additionally, three dose moderators concerning duration of protein supplementation, additional protein supplement dose, and total daily protein intake were incorporated into a moderation analysis to investigate the optimal daily protein consumption and intervention duration for athletes. The analysis utilised a Bayesian framework, allowing for the estimation of effect sizes while accounting for measurement error and hierarchical data structure.

## 3 Results

The results are presented in seven sections as follows: study selection, characteristics of included studies, quality assessment, meta-analysis using fifteen models, moderation analysis, quality grading for each outcome, and publication bias.

### 3.1 Study Selection

**Figure 1.**
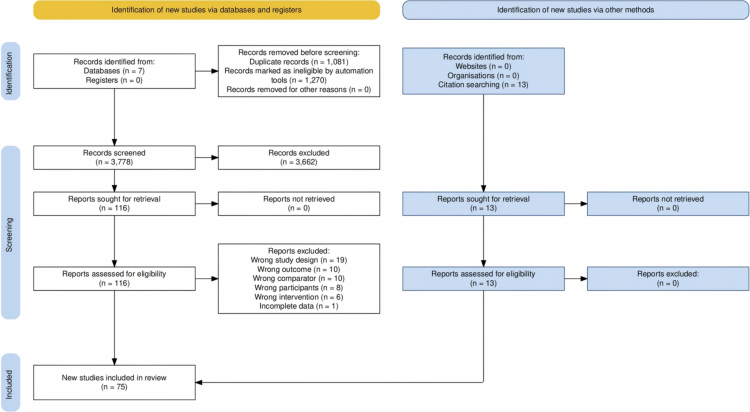
PRISMA flow chart for the identification of the included studies.

Figure 1 illustrates the selection process and information sources. Covidence automatically removed 1,079 duplicate records, and two duplicates were removed manually. A total of 6,129 studies were screened, with 1,270 marked as ineligible by an automatic tool and 3,662 manually excluded as irrelevant. After full-text screening of 116 studies, 54 were excluded, resulting in 62 studies being included in the meta-analysis. An additional 13 RCTs were identified through citation searching, yielding a final total of 75 RCTs.

### 3.2 Characteristics of included studies

The detailed characteristics of each included study are provided in Supplementary File S2. A total of 75 studies involving 1206 athletes (220 females and 916 males) were included, with 70 athletes lacking gender information [54–58]. Twenty-nine studies were randomised controlled trials, and 46 were randomised crossover trials. Geographically, 37 studies (49%) were conducted in Europe, 21 (28%) in North America, seven (9%) in Oceania, six (8%) in Asia, three (4%) in Africa, and one (1%) in South America.

Forty-four studies used protein-carbohydrate supplements as the intervention, while 27 used pure protein supplements. Two studies implemented high-protein diets [59,60] and two utilised enriched protein yoghurts [54] or protein plus probiotic supplements [61] as their interventions. Eleven studies did not report daily energy intake for either the intervention or control groups [10,27,61–69].

Among the 75 included studies, 8 studies (10.67%) did not specify the protein source and another 8 studies (10.67%) used supplements combining multiple protein types. Thirty-five studies (46.67%) used whey protein, six (8.00%) used soy protein, one (1.33%) used branched-chain amino acid (BCAA) supplements, nine (12.00%) used casein protein, and ten (13.33%) used milk protein. Additionally, one study each (1.33%) used hydrolysed collagen, egg white protein, wheat protein, or beef protein.

The participants' sports disciplines included cycling, triathlon, wrestling, boxing, orienteering, running, field hockey, weightlifting, football, soccer, basketball, rugby, volleyball, track and field, sailing, and judo. Nine studies did not report the athletic discipline of the participants [54,59,60,70–75]. Regarding additional protein ingestion from supplements, six studies reported more than 2 g/kg/day [76–81], four studies reported between 1 and 2 g/kg/day [70,82–84], and the remainder reported less than 1 g/kg/day.

### 3.3 Risk of bias assessment

**Figure 2.**
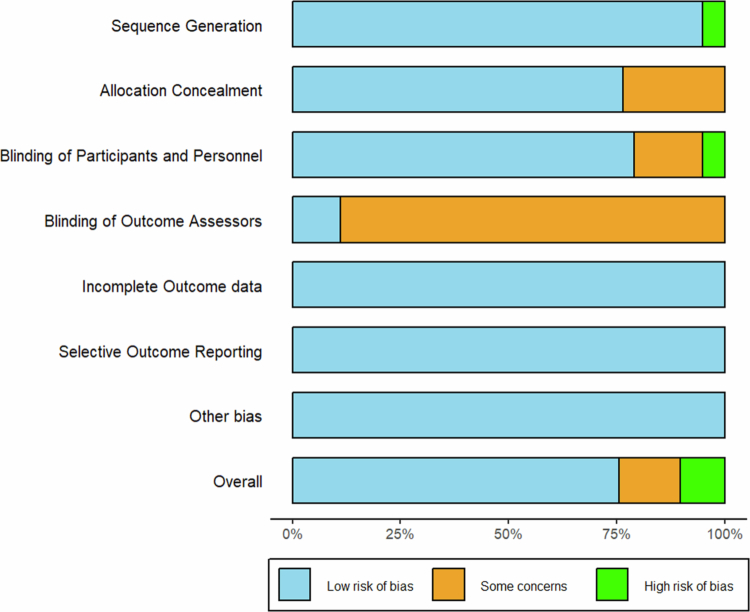
Risk of bias summary.

The risk-of-bias summary is illustrated in Figure 2, and the specific risk-of-bias graph in each study is provided in Supplementary File S3. The Cochrane Risk of Bias tool (RoB) was utilised to assess the included studies, with results visualised using the R package robvis. Some studies were rated as high risk because they used an inappropriate randomisation method, such as block randomisation (4%) [55,75,85], and some authors explicitly acknowledged that the lack of blinding could introduce bias (4%) [86–88]. The majority of studies were rated as having some concerns in the domain of blinding of outcome assessors due to insufficient reporting (84%). Some studies were rated as having some concerns in allocation concealment and blinding of participants and personnel due to a lack of information. Overall, nearly 75% of studies were assessed as low risk of bias, fewer than 15% as moderate risk, and fewer than 10% as high risk.

### 3.4 Meta-analysis

The meta-analysis was divided into fifteen sections, each presenting four outcome measures: muscle strength, endurance performance, glycogen resynthesis, and fatigue recovery. Detailed results in each model are presented in Supplementary File S4. The Markov chain convergence in all fifteen models showed good convergence based on the Rhat parameter. The Rhat value in all results was close to 1.00. Therefore, we did not present the results of Markov chain convergence in the text.

### 3.5 Null model

In the null model, the overall effect size was calculated for four outcomes. All forest plots and density plots of four outcomes in the null model are presented in Supplementary File S5. Sixty-four studies involving 1,048 athletes were included in the analysis of endurance performance. The Bayesian meta-analysis showed a statistically significant effect [μ(SMD): 0.21, 95% CI: 0.07 to 0.35; HDI: 0.07 to 0.34; BF: 2.76], with high between-study heterogeneity and low within-study heterogeneity [τ_within_: 0.10, within I^2^: 3.85%, τ_between_: 0.43, between I^2^: 96.15%]. Thirty studies, including 548 athletes, were included in the analysis of muscle strength in the null model, with no statistically significant effect [μ(SMD): 0.31, 95% CI: –0.01 to 0.64; HDI: –0.01 to 0.63; BF: 0.54], with high between-study heterogeneity and low within-study heterogeneity [τ_within_: 0.06, within I^2^: 0.52%, τ_between_: 0.83, between I^2^: 99.48%].

Thirty-two studies with 425 athletes were included in the analysis of glycogen resynthesis in the null model. No statistically significant effect was observed in the meta-analysis [μ(SMD): 0.17, 95% CI: –0.01 to 0.35; HDI: –0.01 to 0.36; BF: 0.26], with low between-study heterogeneity and moderate within-study heterogeneity [τ_within_: 0.31, within I^2^: 68.54%, τ_between_: 0.21, between I^2^: 31.46%]. Forty-three studies containing 663 athletes were included in the analysis of post-exercise fatigue recovery in the null model. No statistically significant effect was observed in the meta-analysis [μ(SMD): 0.16, 95% CI: –0.01 to 0.33; HDI: –0.01 to 0.32; BF: 0.23], with high between-study heterogeneity and low within-study heterogeneity [τ_within_: 0.16, within I^2^: 13.79%, τ_between_: 0.40, between I^2^: 86.21%].

### 3.6 Supplements model

Five comparisons including protein supplements (PRO) vs. placebo (PLA), protein supplements (PRO) vs. carbohydrate supplements (CHO), protein plus probiotic vs. placebo (PLA), protein-carbohydrate supplements (CHOPRO) vs. carbohydrate supplements (CHO), and protein-carbohydrate supplements (CHOPRO) vs. placebo (PLA) were conducted.

**Figure 3.**
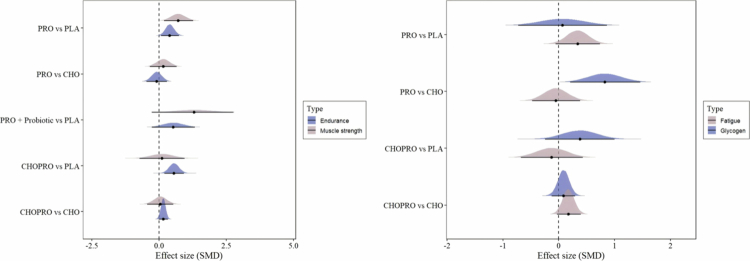
The forest plots in supplements model.

The forest plot is shown in Figure 3. In athletic performance outcomes, including muscle strength and endurance performance, fifteen studies involving 334 athletes were included in the PRO vs. PLA comparison. Statistically significant effects were observed for both endurance performance [μ(SMD): 0.37, 95% CI: 0.02 to 0.71; HDI: 0.07 to 0.73; BF: 1.6] and for muscle strength [μ(SMD): 0.72, 95% CI: 0.18 to 1.27; HDI: 0.18 to 1.26; BF: 4.37]. Eight studies involving 98 athletes were included in the CHOPRO vs. PLA comparison. No statistically significant effect was observed for muscle strength, whereas a statistically significant effect was observed for endurance performance [μ(SMD): 0.57, 95% CI: 0.2 to 0.93; HDI: 0.19 to 0.93; BF: 7.69]. Moderate between-study heterogeneity and low within-study heterogeneity were observed in the supplements model for endurance [τ_within_: 0.08, within I^2^: 2.22%, τ_between_: 0.45, between I^2^: 70.09%] and muscle strength [τ_within_: 0.06, within I^2^: 0.52%, τ_between_: 0.79, between I^2^: 90.75%].

In post-exercise recovery outcomes, including glycogen resynthesis and post-exercise fatigue recovery, eight studies involving 146 athletes were included in the PRO vs. CHO comparison. No statistically significant effect was found for fatigue, whereas a statistically significant effect was observed for glycogen resynthesis [μ(SMD): 0.83, 95% CI: 0.21 to 1.46; HDI: 0.21 to 1.46; BF: 4.84]. Low between-study and within-study heterogeneity were observed in the supplements model for glycogen resynthesis [τ_within_: 0.21, within I^2^: 13.15%, τ_between_: 0.18, between I^2^: 9.92%] and fatigue [τ_within_: 0.15, within I^2^: 7%, τ_between_: 0.37, between I^2^: 42.86%].

### 3.7 Protein source model

**Figure 4.**
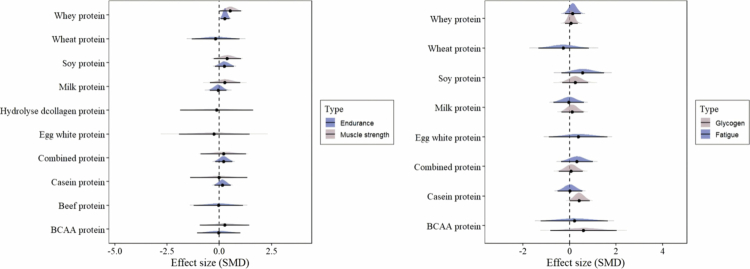
The forest plots in protein source model.

The protein source model divided the data into ten protein types for the analysis of athletic performance outcomes. The forest plot is shown in Figure 4. Only one protein source (whey protein) showed statistically significant effects for both endurance performance [μ(SMD): 0.28, 95% CI: 0.07 to 0.49; HDI: 0.07 to 0.49; BF: 1.52] and muscle strength [μ(SMD): 0.53, 95% CI: 0.01 to 1.05; HDI: 0.004 to 1.05; BF: 1.04]. No statistically significant effects were observed in other types of protein sources. Moderate between-study heterogeneity and low within-study heterogeneity were observed in the supplements model for endurance [τ_within_: 0.09, within I^2^: 2.39%, τ_between_: 0.48, between I^2^: 68.06%] and muscle strength [τ_within_: 0.06, within I^2^: 0.36%, τ_between_: 0.94, between I^2^: 89.51%].

For post-exercise recovery outcomes, the data were divided into ten protein types. In all types of protein sources, no statistically significant effect was observed. Low between-study and within-study heterogeneity were observed in the protein source model for glycogen resynthesis [τ_within_: 0.27, within I^2^: 15.8%, τ_between_: 0.22, between I^2^: 10.49%] and fatigue [τ_within_: 0.16, within I^2^: 7.41%, τ_between_: 0.41, between I^2^: 46.22%].

### 3.8 Protein dose model

In the protein dose model, data were divided into three types (0–1 g/kg, 1–2 g/kg, and 2–3 g/kg) based on the extra protein dose in daily protein supplement ingestion. The forest plot can be seen in Supplementary File S6. In athletic performance, only an extra protein dose ingested with 0–1 g/kg a day from protein supplements showed a statistically significant effect in endurance performance [μ(SMD): 0.25, 95% CI: 0.10 to 0.41; HDI: 0.10 to 0.40; BF: 4.99], with moderate between-study and low within-study heterogeneity [τ_within_: 0.09, within I^2^: 2.78%, τ_between_: 0.44, between I^2^: 66.37%]. Fifty-five studies with 901 athletes were included in this group.

In post-exercise recovery, only the extra protein dose ingested with 0–1 g/kg from protein supplements showed a small statistically significant effect in fatigue recovery [μ(SMD): 0.18, 95% CI: 0.02 to 0.36; HDI: 0.01 to 0.36; BF: 0.44], with low between-study and within-study heterogeneity [τ_within_: 0.14, within I^2^: 7.73%, τ_between_: 0.29, between I^2^: 33.35%]. Although the 95% HDI excluded zero, indicating a small but credible positive effect, the Bayes Factor (BF = 0.44) offered only weak evidence for the alternative hypothesis, suggesting the effect should be interpreted with caution. Thirty-five studies and 551 athletes were included in this group.

### 3.9 Overall dose model

In the overall dose model, the data were divided into three types (0–1 g/kg, 1–2 g/kg, and 2–3 g/kg) based on the overall protein dose in daily protein intake from both protein supplements and three daily meals. The forest plot is illustrated in Supplementary File S6. In terms of athletic performance and post-exercise recovery, no statistically significant effects were observed for endurance, muscle strength, glycogen resynthesis, or fatigue recovery across any of the three protein dose categories.

### 3.10 Protein timing model

In the protein timing model, data were divided into two parts (day and night). The forest plot is shown in Supplementary File S6. Only the group that consumed protein supplements during the day showed a statistically significant effect in endurance performance [μ(SMD): 0.25, 95% CI: 0.09 to 0.41; HDI: 0.09 to 0.40; BF: 3.97], with low between-study and within-study heterogeneity [τ_within_: 0.08, within I^2^: 6.04%, τ_between_: 0.14, between I^2^: 18.49%]. Twenty studies with 313 athletes were included in this group. No statistically significant effect was observed for muscle strength, glycogen resynthesis, and fatigue.

### 3.11 Energy model

In the energy model, data were divided into two groups: energy-matched and energy-unmatched. The forest plot is presented in Supplementary File S6. Statistically significant effects on endurance [μ(SMD): 0.47, 95% CI: 0.24 to 0.70; HDI: 0.24 to 0.70; BF: 147.66] and muscle strength [μ(SMD): 0.52, 95% CI: 0.01 to 1.04; HDI: 0.01 to 1.04; BF: 0.99] were observed only in the energy-unmatched group. Low within-study heterogeneity and moderate between-study heterogeneity were observed in endurance performance [τ_within_: 0.08, within I^2^: 2.35%, τ_between_: 0.42, between I^2^: 64.66%], and low within-study heterogeneity and high between-study heterogeneity were detected in muscle strength [τ_within_: 0.06, within I^2^: 0.47%, τ_between_: 0.84, between I^2^: 91.73%].

In the results of post-exercise recovery, no statistically significant effect was found for either glycogen resynthesis or fatigue recovery.

### 3.12 Fasted model

In the fasted model, data were divided into two groups: fasted and fed. The corresponding forest plot is presented in Supplementary File S6. Regarding athletic performance, a statistically significant effect was observed only for endurance in the fed group [μ(SMD): 0.15, 95% CI: 0.03 to 0.26; HDI: 0.03 to 0.26; BF: 0.60], with low within-study and between-study heterogeneity [τ_within_: 0.06, within I^2^: 4.18%, τ_between_: 0.15, between I^2^: 26.13%]. Although the 95% HDI excluded zero, indicating a small positive effect of feeding on endurance performance, the Bayes Factor (BF = 0.6) provided anecdotal evidence in favour of the null hypothesis, suggesting that the evidence for a true effect remains weak.

For post-exercise recovery outcomes, a statistically significant effect was observed only for fatigue recovery in the fasted group [μ(SMD): 0.30, 95% CI: 0.06 to 0.54; HDI: 0.06 to 0.54; BF: 1.24], with low within-study and between-study heterogeneity [τ_within_: 0.14, within I^2^: 9.36%, τ_between_: 0.2, between I^2^: 13.08%].

### 3.13 Design model

In the design model, data were divided into two groups: parallel and crossover. The corresponding forest plot is presented in Supplementary File S6. Regarding athletic performance, a statistically significant effect was observed for endurance [μ(SMD): 0.39, 95% CI: 0.14 to 0.65; HDI: 0.14 to 0.65; BF: 5.78] and muscle strength [μ(SMD): 0.53, 95% CI: 0.01 to 0.97; HDI: 0.10 to 0.96; BF: 2.01] only in the parallel group. Low within-study heterogeneity and moderate between-study heterogeneity were observed in endurance performance [τ_within_: 0.09, within I^2^: 2.78%, τ_between_: 0.44, between I^2^: 66.37%], and low within-study heterogeneity and high between-study heterogeneity were found in muscle strength [τ_within_: 0.06, within I^2^: 0.50%, τ_between_: 0.81, between I^2^: 91.16%].

For post-exercise recovery outcomes, no statistically significant effect was observed in either the parallel or crossover group. The corresponding forest plot is presented in Supplementary File S6.

### 3.14 Acute-chronic model

In the acute-chronic model (acute vs chronic), a statistically significant effect on muscle strength was observed in the chronic protein group [μ(SMD): 0.5, 95% CI: 0.05 to 0.96; HDI: 0.05 to 0.95; BF: 1.36], with low within-study heterogeneity and high between-study heterogeneity [τ_within_: 0.06, within I^2^: 0.48%, τ_between_: 0.83, between I^2^: 91.55%]. For endurance performance, a statistical significance was found in both the chronic protein group [μ(SMD): 0.26, 95% CI: 0.03 to 0.49; HDI: 0.03 to 0.49; BF: 0.66] and the acute protein group [μ(SMD): 0.18, 95% CI: 0.001 to 0.36; HDI: 0.004 to 0.37; BF: 0.33], with low within-study heterogeneity and moderate between-study heterogeneity [τ_within_: 0.08, within I^2^: 2.14%, τ_between_: 0.45, between I^2^: 67.75%]. While both posterior distributions excluded zero, indicating credible positive effects, the Bayes Factors (chronic: BF = 0.66; acute: BF = 0.33) suggested only anecdotal to weak support for the alternative hypothesis, with stronger uncertainty observed in the acute group. The corresponding forest plot is presented in Supplementary File S6.

### 3.15 Endurance model

In the endurance model, a statistically significant effect was observed in aerobic [μ(SMD): 0.23, 95% CI: 0.04 to 0.42; HDI: 0.03 to 0.42; BF: 0.74] and anaerobic performance [μ(SMD): 0.47, 95% CI: 0.05 to 0.88; HDI: 0.05 to 0.88; BF: 1.21] when assessed through cycling-based tests, with low within-study heterogeneity and moderate between-study heterogeneity [τ_within_: 0.09, within I^2^: 2.69%, τ_between_: 0.45, between I^2^: 67.37%]. The corresponding forest plot is presented in Supplementary File S6.

### 3.16 Muscle model

In the muscle model, a statistically significant effect was observed only in lower body strength [μ(SMD): 0.52, 95% CI: 0.06 to 0.97; HDI: 0.05 to 0.97; BF: 1.47], with low within-study heterogeneity and high between-study heterogeneity [τ_within_: 0.07, within I^2^: 0.67%, τ_between_: 0.81, between I^2^: 89.75%]. The corresponding forest plot is presented in Supplementary File S6.

### 3.17 Glycogen model

In the glycogen model, no statistically significant effect was observed in either glucose or muscle glycogen indicators. The corresponding forest plot is presented in Supplementary File S6.

### 3.18 Fatigue model

In the fatigue model, a statistically significant effect was found only in fatigue index [μ(SMD): 1.06, 95% CI: 0.25 to 1.90; HDI: 0.24 to 1.89; BF: 5.63], with low within-study and between-study heterogeneity [τ_within_: 0.14, within I^2^: 6.55%, τ_between_: 0.36, between I^2^: 43.32%]. The corresponding forest plot is presented in Supplementary File S6.

### 3.19 Interaction model

In the interaction model one (Endurance × Energy), a statistically significant effect was only observed in the energy-unmatched condition for anaerobic endurance performance [μ(SMD): 1.38, 95% CI: 0.19 to 2.46], with low within-study and between-study heterogeneity [τ_within_: 0.1, within I^2^: 5.18%, τ_between_: 0.27, between I^2^: 37.79%].

In the interaction model two (Muscle Strength × Protein Timing), no statistically significant effects were observed in any timing condition across all muscle strength indicators.

In the interaction model three (Muscle strength × Supplements), a statistically significant effect was only observed in jump performance (PRO vs PLA) [μ(SMD): 2.21, 95% CI: 0.97 to 3.41], with low within-study and moderate between-study heterogeneity [τ_within_: 0.07, within I^2^: 2.97%, τ_between_: 0.3, between I^2^: 54.58%].

Compared to the previous models, all interaction models showed a substantial reduction in heterogeneity. The heterogeneity in the original endurance performance model primarily stemmed from differences in energy matching between groups, while the heterogeneity in the muscle strength model was mainly attributed to variations in protein timing and types of protein supplements.

### 3.20 Moderation analysis (linear regression)

**Figure 5.**
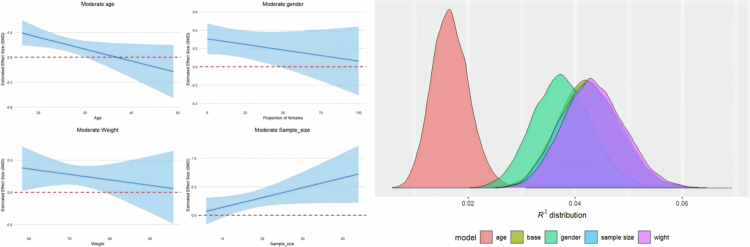
The regression plots and R^2^ density plot moderated by age, weight, sample size and gender in athletic performance.

**Figure 6.**
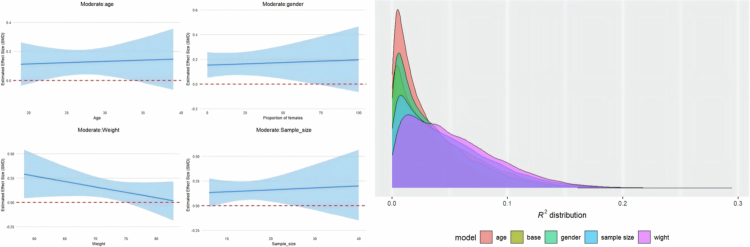
The regression plots and R^2^ density plot moderated by age, weight, sample size and gender in post-exercise recovery.

Four trait moderators were added to the meta-regression. The regression plot is presented in Figure 5 for athletic performance and in Figure 6 for post-exercise recovery, while the detailed results are provided in Supplementary File S7.

In terms of athletic performance, age was the only moderator that showed a significant negative effect, indicating that the effectiveness of protein on athletic performance decreases with increasing age [coefficient estimate: –0.02, 95% CI: –0.04 to –0.001; R² = 2%]. Other moderators were not found to have statistical significance in athletic performance. However, from the graph, the influence of weight and female proportion showed a negative trend.

In post-exercise recovery, no moderators were found to have a significant effect.

Based on the trait moderation analysis, the models including the four trait moderators all showed low R² values, indicating that they cannot fully explain the heterogeneity. Therefore, it is unlikely that the heterogeneity originates from these four moderators (age, weight, sample size, and gender).

### 3.21 Moderation analysis (non-linear regression)

**Figure 7.**
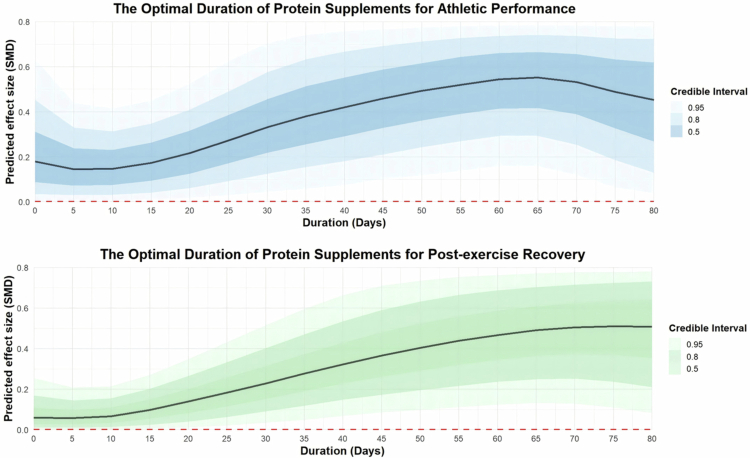
Moderation analysis of protein supplementation duration on athletic performance and post-exercise recovery.

Three dose moderators, duration of protein supplementation (in days), extra protein supplement dose and total daily protein intake, were added to the moderation analysis. Regarding the duration of protein supplement interventions, the results suggest that a period of 40 to 65 days is optimal for enhancing athletic performance [40 days: coefficient estimate: 0.45, 95% CI: 0.07 to 0.88; R²: 4%; 65 days: coefficient estimate: 0.60, 95% CI: 0.23 to 0.99; R²: 4%], while a duration of 40 to 80 days appears suitable for promoting post-exercise recovery [40 days: coefficient estimate: 0.33, 95% CI: 0.08 to 0.62; R²: 10%; 80 days: coefficient estimate: 0.57, 95% CI: 0.10 to 1.05; R²: 10%]. The regression plots are presented in Figure 7.

**Figure 8.**
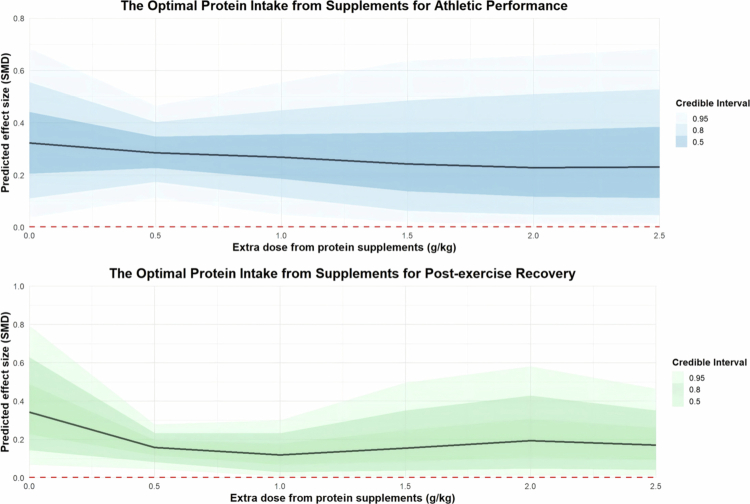
Moderation analysis of extra protein dose on athletic performance and post-exercise recovery.

Regarding the extra protein dose from supplements, the results show that a daily intake of 1 g/kg of additional protein from supplements [coefficient estimates: 0.27, 95% CI: 0.05 to 0.50; R²: 4%] is more effective for improving athletic performance than 1.5 g/kg [coefficient estimates: 0.20, 95% CI: –0.16 to 0.56; R²: 4%] and 2 g/kg [coefficient estimates: 0.13, 95% CI: –0.33 to 0.57; R²: 4%]. For promoting post-exercise recovery, an extra dose of 0.5 g/kg [coefficient estimates: 0.16, 95% CI: 0.06 to 0.26; R²: 7%] shows better outcomes compared to 1 g/kg [coefficient estimates: 0.09, 95% CI: –0.1 to 0.26; R²: 7%] and 2 g/kg [coefficient estimates: 0.17, 95% CI: –0.11 to 0.49; R²: 7%]. The regression plots are presented in Figure 8.

**Figure 9.**
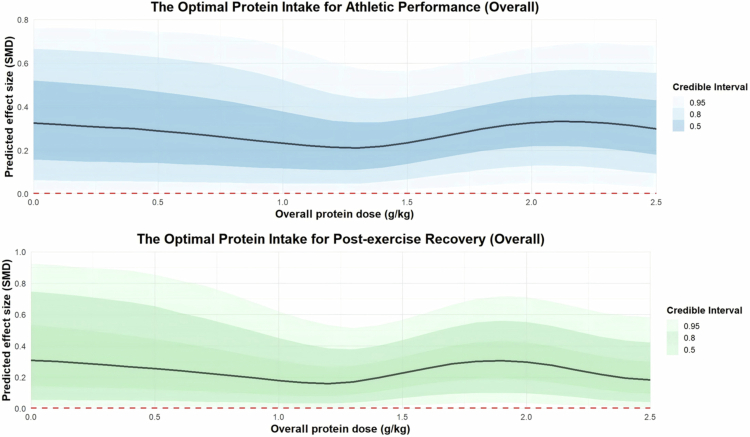
Moderation analysis of overall protein dose on athletic performance and post-exercise recovery.

Regarding total daily protein intake, the results indicate that consuming 2 g/kg per day yields better improvements in athletic performance [coefficient estimate: 0.33, 95% CI: 0.05 to 0.63; R²: 63%] compared to 1 g/kg [coefficient estimate: 0.05, 95% CI: –0.52 to 0.56; R²: 63%] and 1.5 g/kg [coefficient estimate: 0.21, 95% CI: –0.05 to 0.50; R²: 63%]. For promoting post-exercise recovery, an intake of 2 g/kg also shows more favourable effects [coefficient estimate: 0.30, 95% CI: 0.001 to 0.63; R²: 21%] than 1 g/kg [coefficient estimate: –0.02, 95% CI: –0.50 to 0.44; R²: 21%] and 1.5 g/kg [coefficient estimate: 0.21, 95% CI: –0.06 to 0.49; R²: 21%]. The regression plots are presented in Figure 9.

The R² density plots are presented in Supplementary File S8. The results indicate that the overall protein dose model is the optimal model for both athletic performance and post-exercise recovery (R² for performance: 63%; R² for recovery: 21%).

### 3.22 Quality grade in each outcome

**Figure 10.**
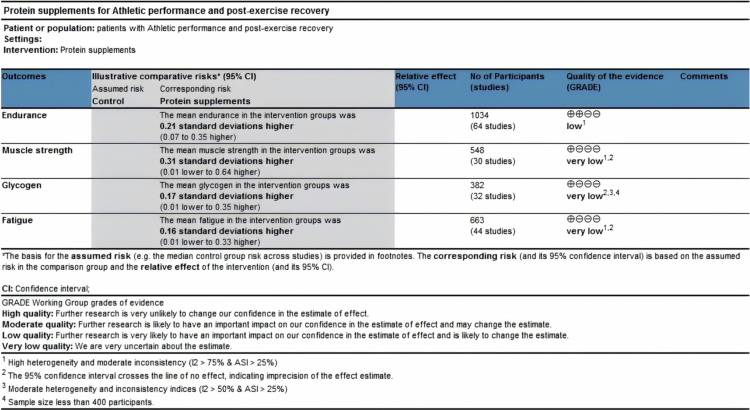
Summary of evidence quality using the GRADE approach.

The quality grade for each outcome was determined based on sample size, meta-analysis results, and quality assessment, including risk of bias, result inconsistency, indirectness, and imprecision of effect estimates. The results showed that the quality of evidence for muscle strength, glycogen resynthesis, and fatigue was rated as very low due to high heterogeneity or inconsistency, non-significant findings, and small sample sizes. The quality of evidence for endurance performance was rated as low because of high heterogeneity and inconsistency. The GRADE summary is presented in Figure 10.

Additionally, we conducted GRADE assessments for all models. In the interaction models, we found that heterogeneity was generally reduced, leading to upgraded quality ratings (high quality for endurance and moderate quality for muscle strength). The previously observed high heterogeneity in the null model for endurance performance was mainly attributed to differences among endurance indicators and variations in macronutrient distribution. For muscle strength, the high heterogeneity in the null model stemmed from differences in strength measurement indicators, timing of protein intake, and types of supplementation used. The additional GRADE summary is provided in Supplementary File S9.

### 3.23 Publication bias

The funnel plots generated by the publicationbias and metafor packages are illustrated in Supplementary File S10. First, a multilevel Egger's test indicated the presence of publication bias in the athletic performance data, including both endurance and muscle strength outcomes (*P* < 0.0001), whereas no such bias was detected in the post-exercise recovery data (*P* = 0.27). Second, significance funnel plots generated using the PublicationBias package revealed that most studies were non-significant across all outcome types. However, the grey diamond (representing non-significant studies only) was closely aligned with the black diamond (representing all studies), suggesting that the overall findings were not substantially affected by publication bias. Third, the computed s-values indicated that, for both endurance and muscle strength, no plausible level of publication bias would be sufficient to attenuate the observed effects to null (s-value = not possible), supporting the robustness of the results.

Additionally, a trim-and-fill analysis using the metafor package further confirmed the robustness of the athletic performance data, with no imputed studies and no clear asymmetry observed in the funnel plot.

## 4 Discussion

This is the first multilevel meta-analysis to explore the efficacy of different types of protein supplements, protein sources, protein timing, extra doses of protein supplements, and overall daily protein doses on athletic performance and post-exercise recovery. The current systematic review and meta-analysis summarise the evidence on the effects of (1) protein supplements on athletic performance, as well as (2) protein supplements on post-exercise recovery.

### 4.1 Summary of findings

The results indicate that protein supplements offer significant benefits for both athletic performance and post-exercise recovery. Protein-carbohydrate supplements were found to improve endurance when compared to placebo supplements, though they did not improve muscle strength. In contrast, pure protein supplements enhanced both muscle strength and endurance relative to the placebo group, with no significant differences observed compared with carbohydrate supplements. Both protein-carbohydrate and pure protein supplements promoted more efficient glycogen resynthesis than placebo, and pure protein supplements additionally reduced fatigue. However, all significant findings were derived from studies in which the energy ratios between the experimental and control groups were unequal.

### 4.2 Protein supplements and athletic performance

The effect of protein supplements on athletic performance is weak and only marginally significant, and even when statistical significance is detected, it originates from studies with unbalanced energy intake between the intervention and control groups [10,11,27,37,61–63,65–67,69,89–94]. In these energy-unmatched studies, protein supplementation was found to improve anaerobic and aerobic endurance in cycling tests, as well as lower-body strength in athletes. Whey protein remains the most effective type for improving athletic performance; however, the absence of significant effects for other protein types may result from the limited number of studies examining them.

Firstly, regarding endurance performance, many studies support the conclusion that in isocaloric trials, endurance performance does not improve with pure protein or protein-carbohydrate supplementation compared with control groups [10,69,95,96]. Jäger et al. concluded that adding protein to a carbohydrate beverage before or during endurance exercise does not generally improve performance, especially in isocaloric conditions [8]. Another review further noted that such performance improvements are likely attributable to the additional energy provided by protein co-ingestion, rather than protein itself [97]. Moreover, protein supplementation may offer greater benefits for individuals with low aerobic capacity, but not for those capable of substantial VO_2_max improvements through endurance or high-intensity interval training [94]. Additionally, when daily nitrogen balance is already positive, the benefits of protein supplementation on subsequent endurance performance appear to be negligible [98]. Therefore, current evidence suggests that protein supplementation is not essential for enhancing endurance performance, particularly in well-trained athletes or when adequate carbohydrate intake is ensured.

Second, the effect of protein supplementation on muscle strength is similarly limited and appears to be even less effective than its impact on endurance performance. Long-term protein interventions tend to show better results than acute (single-dose) supplementation. Positive effects are primarily observed in improvements in lower-body strength, with many studies providing supportive evidence for this finding [54,70,71,99,100]. Jäger et al. summarised that protein supplementation exerts a small to modest impact on strength development in both men and women. While findings from individual studies are mixed, pooled analyses support a modest benefit, particularly when combined with sufficient training duration and total protein intake [8]. Recent evidence indicates that protein supplementation during resistance training results in small but significant gains in lean body mass and lower-body strength, especially in younger or trained individuals, while having minimal effects on fat mass, handgrip strength, and muscle fibre hypertrophy [32,101]. Additionally, a systematic review found that acute protein supplementation enhances myofibrillar protein synthesis following concurrent exercise, while longer-term supplementation shows inconsistent but sometimes positive effects on muscle mass and strength, with no benefits for aerobic capacity [102]. Therefore, protein supplementation should be viewed as a complementary strategy that supports muscular adaptations, particularly when combined with structured resistance training, but its benefits are limited and context-dependent.

Third, whey protein is superior to other types of protein sources and provides the majority of the benefits. However, this meta-analysis included a large number of studies that investigated whey protein (35 included studies), suggesting that no significant effects were found on other protein types, which may be due to insufficient studies. Although no significant effect was found, soy protein (6 included studies) appears to be the most promising alternative to whey protein for athletes. In the future, more robust evidence will be needed to prove this. Whey protein appears to play a role in enhancing lymphatic and immune system responses, making it an ideal protein source for athletes [8,103]. The latest review [104] shows that soy protein appears to be an effective alternative to whey protein in promoting optimal muscle mass and strength gains, but the data are limited, and its amino acid content is lower than that of whey protein. Therefore, its true effectiveness requires further evaluation in future studies.

In the moderation analysis, results indicated that older athletes experienced less benefit from protein supplementation. Evidence suggests that an additional intake of approximately 1 g/kg/day from supplements, resulting in a total daily protein intake of around 2 g/kg/day, is most effective for enhancing athletic performance. Furthermore, intervention durations between 40 and 65 days appear to yield the most favourable outcomes. These findings are consistent with previous research demonstrating an age-related decline in the efficacy of protein supplementation, as well as the importance of optimising dosage and duration. Specifically, Jäger et al. concluded that protein supplementation of 15 to 25 g per day over a period of 4 to 21 weeks positively influences performance [8]. It has also been reported that increasing age reduces the effectiveness of protein supplementation during resistance training, whereas greater training experience enhances it [105].

Regarding optimal intake levels, the recommended daily protein intake for maximising strength gains is above 1.6 g/kg/day [101,105], while for endurance athletes, approximately 1.85 g/kg/day appears to be most effective [106]. Additionally, for athletes aiming to maintain an anabolic environment, particularly during periods of energy restriction, higher protein intakes may be necessary. A daily intake of 1.4 to 2.0 g/kg/day is generally considered the minimum requirement, with greater amounts potentially needed to preserve fat-free mass [8]. These insights highlight the necessity of individualised protein strategies that consider factors such as age, training experience, exercise type, and nutritional status to optimise athletic performance.

In the moderation analysis regarding gender, although the results were not statistically significant, the regression plot suggested a negative trend. Whether protein intake is influenced by gender remains unclear. Most current studies have been conducted primarily on male participants [107,108], highlighting the need for more research focusing on female subjects to explore potential differences in the effects of protein supplementation between males and females. Moore et al. concluded that it is too early to recommend sex-specific carbohydrate or protein guidelines for female athletes if their energy needs are met. More research is needed using sport-specific protocols that control for factors such as prior exercise, nutritional status, contraceptive use, and menstrual cycle phase [109]. Therefore, future studies should prioritise gender-specific investigations to determine whether physiological and hormonal differences influence protein metabolism and supplementation outcomes. Such research is essential for developing tailored nutritional strategies that optimise performance and recovery in both male and female athletes.

### 4.3 Protein supplements and post-exercise recovery

Due to length constraints, examining the relationship between protein supplementation and all aspects of post-exercise recovery was challenging. Therefore, this meta-analysis focused on two key components: glycogen resynthesis (i.e. muscle glycogen and blood glucose) and fatigue. Unfortunately, only a small number of results showed statistical significance, and the observed significance was primarily attributable to studies in which the experimental and control groups had unequal energy intake, rather than the independent effect of protein supplementation.

Several studies have provided supporting evidence. Jäger et al. summarised that when combined with sub-optimal intake of carbohydrates (<1.2 g/kg/day), protein-carbohydrate supplements probably can heighten muscle glycogen recovery and may help mitigate changes in muscle damage markers [8]. Additionally, an RCT involving eight cyclists found protein-carbohydrate supplements could increase muscle glycogen by 128%, but these benefits may be due to the energy intake from extra carbohydrate ingestion, not the protein itself [110].

In terms of post-exercise fatigue recovery, only a statistically significant effect was observed in the fatigue index measured by the Wingate test. Some studies provided supporting evidence. Mhamed et al. concluded that the fatigue index reflects the degree of anaerobic fatigue by measuring the decline in power output during high-intensity exercise. Its improvement is likely due to increased muscle protein synthesis, reduced muscle inflammation, and timely protein intake post-exercise, which together enhance muscle recovery and endurance capacity [111]. A systematic review by Pasiakos et al. investigated the relationship between protein and muscle soreness [112]. In this review, some included studies showed a decrease in muscle soreness in groups consuming protein after initial exercise bout [10,113,114], whereas others did not [91,115,116]. Therefore, when protein supplements are provided, acute changes in post-exercise protein synthesis and anabolic intracellular signalling have not resulted in measurable reductions in muscle damage and enhanced recovery of muscle function [112]. The protein-rich supplementation regime seems to attenuate exercise-induced muscular and inflammatory stress responses [94]. However, apart from the fatigue index, no significant effects were observed in blood lactate levels or subjective fatigue assessments, indicating that the impact of protein supplementation on fatigue recovery requires further investigation. Additionally, some relevant biomarkers, such as creatine kinase (CK), were not included in this analysis, thus no definitive conclusion can be drawn regarding the effect of protein supplementation on fatigue recovery.

### 4.4 Limitations

Several limitations should be acknowledged. First, although interaction models reduced heterogeneity, the limited sample size (~1,000 athletes) and persistent high heterogeneity in the main models led to multiple downgrades, thereby limiting the overall quality and credibility. More studies that include a large sample size are needed to provide robust evidence. Second, a potential risk of bias in the athletic performance outcomes reduces the credibility of the results. Third, this meta-analysis cannot provide comprehensive conclusions across all aspects of protein supplementation, particularly regarding athletic performance and post-exercise recovery. For instance, further research is required to clarify the efficacy of protein timing and sources. The limited number of studies has contributed to the inconsistency of the results. Moreover, key recovery-related biomarkers, such as CK which reflects muscle damage, were not extracted in this analysis; this omission may partly explain the negative findings. Finally, we found that the significant improvement in endurance performance associated with protein supplementation was mainly observed in studies where participants were not fasted before the intervention. This suggests that the observed effects may result from additional dietary intake rather than protein itself. Future studies should adopt more rigorous experimental designs to eliminate confounding factors and clarify the true effect of protein supplementation.

## 5 Conclusion

Overall, the effects of protein supplementation on athletes' performance and post-exercise recovery appear to be limited. The significant findings observed so far mostly come from studies with unequal energy matching between groups. In these studies, protein supplementation showed significant improvements in cycling endurance (both anaerobic and aerobic), lower-body strength, and the fatigue index. An additional protein intake of approximately 1 g/kg/day from supplements, combined with a total daily protein intake of approximately 2 g/kg/day, for an intervention period of 40 to 65 days, was identified as the most effective dosage and duration for performance enhancement. Long-term protein supplementation demonstrated greater improvements in muscle strength and endurance compared with acute protein intake.

In protein supplementation studies, factors such as the consumption of other foods by athletes prior to performance testing and the use of randomised crossover designs may introduce bias, potentially contributing to negative results. Therefore, athletes should tailor protein supplement dosages to their individual needs. Meanwhile, future research should involve large-sample, high-quality studies exploring potential moderators such as protein type, supplementation timing, and sex differences.

## Supplementary Material

supplementary material Supplementary_file_S12_The_included_literature_in_meta_analysis.

supplementary material Supplementary_file_S11_Graphical_Abstract.

supplementary material Supplementary_file_S10.

supplementary material Supplementary_file_S9.

supplementary material Supplementary_file_S8.

supplementary material xxxxxx.

supplementary material Supplementary_file_S6.

supplementary material Supplementary_file_S5.

supplementary material Supplemental Material.

supplementary material Supplementary_file_S3.

supplementary material xxxxxx.

supplementary material Supplementary_file_S1.

supplementary material Supplemental Material.

## Data Availability

All data and supplementary files used in this study, including graphs, codes in R, and results, have been uploaded to the OSF database for sharing. (https://osf.io/y25va/?view_only=b8b9aa4fef4443dd88647974b6b3d7b8).

## References

[1] Campbell BI, Spano MA, editors. National Strength & Conditioning Association (U.S.) NSCA’s Guide to Sport and Exercise Nutrition. Human Kinetics; 2011.

[2] Benardot D. ACSM’s. Nutrition for exercise science. Wolters Kluwer Health; 2018.

[3] Dunford M, Doyle JA, Killian L. Nutrition for sport and exercise. 5th ed. Cengage; 2022.

[4] Campbell B, Kreider RB, Ziegenfuss T, et al. International society of sports nutrition position stand: protein and exercise. J Int Soc Sports Nutr. 2007;4(1):8. doi: 10.1186/1550-2783-4-8 17908291 PMC2117006

[5] Knuiman P, Hopman MTE, Verbruggen C, et al. Protein and the adaptive response with endurance training: wishful thinking or a competitive edge? Front Physiol. 2018;9:598. doi: 10.3389/fphys.2018.00598 29875696 PMC5974122

[6] López-Martínez MI, Miguel M, Garcés-Rimón M. Protein and sport: alternative sources and strategies for bioactive and sustainable sports nutrition. Front Nutr. 2022;9. doi: 10.3389/fnut.2022.926043 PMC9247391 35782926

[7] Nutrition and Athletic Performance. Med Sci Sports Exerc. 2016;48(3):543–568. doi: 10.1249/MSS.0000000000000852 26891166

[8] Jäger R, Kerksick CM, Campbell BI, et al. International society of sports nutrition position stand: protein and exercise. J Int Soc Sports Nutr. 2017;14(1):20. doi: 10.1186/s12970-017-0177-8 28642676 PMC5477153

[9] McLellan TM, Pasiakos SM, Lieberman HR. Effects of protein in combination with carbohydrate supplements on acute or repeat endurance exercise performance: a systematic review. Sports Med. 2014;44(4):535–550. doi: 10.1007/s40279-013-0133-y 24343835

[10] Romano-Ely BC, Todd MK, Saunders MJ, et al. Effect of an isocaloric carbohydrate-protein-antioxidant drink on cycling performance. Med Sci Sports Exerc. 2006;38(9):1608–1616. doi: 10.1249/01.mss.0000229458.11452.e9 16960522

[11] Van Essen M, Gibala MJ. Failure of protein to improve time trial performance when added to a sports drink. Med Sci Sports Exerc. 2006;38(8):1476–1483. doi: 10.1249/01.mss.0000228958.82968.0a 16888462

[12] Osterberg KL, Zachwieja JJ, Smith JW. Carbohydrate and carbohydrate + protein for cycling time-trial performance. J Sports Sci. 2008;26(3):227–233. doi: 10.1080/02640410701459730 18074296

[13] Breen L, Tipton KD, Jeukendrup AE. No effect of carbohydrate-protein on cycling performance and indices of recovery. Med Sci Sports Exerc. 2010;42(6):1140–1148. doi: 10.1249/MSS.0b013e3181c91f1a 19997018

[14] Bagheri R, Kargarfard M, Sadeghi R, et al. Effects of 16 weeks of two different high-protein diets with either resistance or concurrent training on body composition, muscular strength and performance, and markers of liver and kidney function in resistance-trained males. J Int Soc Sports Nutr. 2023;20(1):2236053. doi: 10.1080/15502783.2023.2236053 37516903 PMC10388821

[15] Berg A, Schaffner D, Pohlmann Y, et al. A soy-based supplement alters energy metabolism but not the exercise-induced stress response. Published online. 2012 22876725

[16] Fritz P, Fritz R, Bóday P, et al. Gut microbiome composition: link between sports performance and protein absorption? J Int Soc Sports Nutr. 2024;21(1):2297992. doi: 10.1080/15502783.2023.2297992 38151716 PMC10763846

[17] Hartono FA, Martin-Arrowsmith PW, Peeters WM, et al. The effects of dietary protein supplementation on acute changes in muscle protein synthesis and longer-term changes in muscle mass, strength, and aerobic capacity in response to concurrent resistance and endurance exercise in healthy adults: a systematic review. Sports Med. 2022;52(6):1295–1328. doi: 10.1007/s40279-021-01620-9 35113389

[18] Highton J, Twist C, Lamb K, et al. Carbohydrate-protein coingestion improves multiple-sprint running performance. J Sports Sci. 2013;31(4):361–369. doi: 10.1080/02640414.2012.735370 23134234

[19] Kloby Nielsen LL, Tandrup Lambert MN, Jeppesen PB. The effect of ingesting carbohydrate and proteins on athletic performance: a systematic review and meta-analysis of randomized controlled trials. Nutrients. 2020;12(5):1483. doi: 10.3390/nu12051483 32443678 PMC7284704

[20] Lam FC, Bukhsh A, Rehman H, et al. Efficacy and safety of whey protein supplements on vital sign and physical performance among athletes: a network meta-analysis. Front Pharmacol. 2019;10:317. doi: 10.3389/fphar.2019.00317 31068804 PMC6491698

[21] Lim MT, Pan BJ, Toh DWK, et al. Animal protein versus plant protein in supporting lean mass and muscle strength: a systematic review and meta-analysis of randomized controlled trials. Nutrients. 2021;13(2):661. doi: 10.3390/nu13020661 33670701 PMC7926405

[22] Moore DR, Camera DM, Areta JL, et al. Beyond muscle hypertrophy: why dietary protein is important for endurance athletes. Appl Physiol Nutr Metab. 2014;39(9):987–997. doi: 10.1139/apnm-2013-0591 24806440

[23] Pasiakos SM, McLellan TM, Lieberman HR. The effects of protein supplements on muscle mass, strength, and aerobic and anaerobic power in healthy adults: a systematic review. Sports Med. 2015;45(1):111–131. doi: 10.1007/s40279-014-0242-2 25169440

[24] Pourabbas M, Bagheri R, Hooshmand Moghadam B, et al. Strategic ingestion of high-protein dairy milk during a resistance training program increases lean mass, strength, and power in trained young males. Nutrients. 2021;13(3):948. doi: 10.3390/nu13030948 33804259 PMC7999866

[25] Qin L, Sun FH, Huang Y, et al. Effect of pre-exercise ingestion of *α* -lactalbumin on subsequent endurance exercise performance and mood states. Br J Nutr. 2019;121(1):22–29. doi: 10.1017/S000711451800274X 30588901

[26] Rustad PI, Sailer M, Cumming KT. Intake of protein plus carbohydrate during the first two hours after exhaustive cycling improves performance the following day In: Eynon N, editor. PLOS One. 2016;11(4): e0153229. doi: 10.1371/journal.pone.0153229 PMC4831776 27078151

[27] Shenoy S, Dhawan M, Singh Sandhu J. Four weeks of supplementation with isolated soy protein attenuates exercise-induced muscle damage and enhances muscle recovery in well trained athletes: a randomized trial. Asian J Sports Med. 2016;7(3). doi: 10.5812/asjsm.33528 PMC5098124 27826398

[28] Stearns RL, Emmanuel H, Volek JS, et al. Effects of ingesting protein in combination with carbohydrate during exercise on endurance performance: a systematic review with meta-analysis. J Strength Cond Res. 2010;24(8):2192–2202. doi: 10.1519/JSC.0b013e3181ddfacf 20683237

[29] Valenzuela PL, Mata F, Morales JS, et al. Does beef protein supplementation improve body composition and exercise performance? a systematic review and meta-analysis of randomized controlled trials. Nutrients. 2019;11(6):1429. doi: 10.3390/nu11061429 31242624 PMC6628355

[30] Zare R, Devrim-Lanpir A, Guazzotti S, et al. Effect of soy protein supplementation on muscle adaptations, metabolic and antioxidant status, hormonal response, and exercise performance of active individuals and athletes: a systematic review of randomised controlled trials. Sports Med. 2023;53(12):2417–2446. doi: 10.1007/s40279-023-01899-w 37603200 PMC10687132

[31] Cintineo HP, Arent MA, Antonio J, et al. Effects of protein supplementation on performance and recovery in resistance and endurance training. Front Nutr. 2018;5:83. doi: 10.3389/fnut.2018.00083 30255023 PMC6142015

[32] Cermak NM, Res PT, De Groot LC, et al. Protein supplementation augments the adaptive response of skeletal muscle to resistance-type exercise training: a meta-analysis. AJCN. 2012;96(6):1454–1464. doi: 10.3945/ajcn.112.037556 23134885

[33] Loureiro LL, Ferreira TJ, Cahuê FLC, et al. Comparison of the effects of pea protein and whey protein on the metabolic profile of soccer athletes: a randomized, double-blind, crossover trial. Front Nutr. 2023;10:1210215. doi: 10.3389/fnut.2023.1210215 37810915 PMC10556705

[34] Nieman DC, Zwetsloot KA, Simonson AJ, et al. Effects of whey and pea protein supplementation on post-eccentric exercise muscle damage: a randomized trial. Nutrients. 2020;12(8):2382. doi: 10.3390/nu12082382 32784847 PMC7468723

[35] Teixeira FJ, Matias CN, Faleiro J, et al. A novel plant-based protein has similar effects compared to whey protein on body composition, strength, power, and aerobic performance in professional and semi-professional futsal players. Front Nutr. 2022;9:934438. doi: 10.3389/fnut.2022.934438 35938106 PMC9355667

[36] Trommelen J, Van Loon L. Pre-sleep protein ingestion to improve the skeletal muscle adaptive response to exercise training. Nutrients. 2016;8(12):763. doi: 10.3390/nu8120763 27916799 PMC5188418

[37] Miles KH, Clark B, Fowler PM, et al. ɑ-Lactalbumin improves sleep and recovery after simulated evening competition in female athletes. Med Sci Sports Exerc. 2021;53(12):2618–2627. doi: 10.1249/MSS.0000000000002743 34649262

[38] Abbott W, Brett A, Cockburn E, et al. Presleep casein protein ingestion: acceleration of functional recovery in professional soccer players. Int J Sports Physiol Perform. 2019;14(3):385–391. doi: 10.1123/ijspp.2018-0385 30204517

[39] Haddaway NR, Page MJ, Pritchard CC, et al. *PRISMA2020*: an R package and shiny app for producing prisma 2020-compliant flow diagrams, with interactivity for optimised digital transparency and open synthesis. Campbell Syst Rev. 2022;18(2):e1230. doi: 10.1002/cl2.1230 36911350 PMC8958186

[40] McGuinness LA, Higgins JPT. Risk‐of‐bias VISualization (robvis): an R package and shiny web app for visualizing risk‐of‐bias assessments. Res Synth Methods. 2021;12(1):55–61. doi: 10.1002/jrsm.1411 32336025

[41] Mathur MB, VanderWeele TJ. Sensitivity Analysis for Publication Bias in Meta-Analyses. 10.1111/rssc.12440 PMC7590147 33132447

[42] Braginsky M, Mathur M, VanderWeele TJ. PublicationBias: sensitivity analysis for publication bias in meta-analyses. Published online. 2019:2.4.0. doi: 10.32614/CRAN.package.PublicationBias PMC7590147 33132447

[43] Digitizer GG. Getdata-Graph-Digitizer. Available online. 2020. 2020;http://getdata-graph-digitizer.com/

[44] Abbas A, Hefnawy MT, Negida A. Meta-analysis accelerator: a comprehensive tool for statistical data conversion in systematic reviews with meta-analysis. BMC Med Res Methodol. 2024;24(1):243. doi: 10.1186/s12874-024-02356-6 39425031 PMC11487830

[45] Follmann D, Elliott P, Suh I, et al. Variance imputation for overviews of clinical trials with continuous response. J Clin Epidemiol. 1992;45(7):769–773. doi: 10.1016/0895-4356(92)90054-Q 1619456

[46] Bürkner PC. Advanced Bayesian multilevel modeling with the R package brms. R J. 2018;10(1):395. doi: 10.32614/RJ-2018-017

[47] Sousa-Pinto B, Vieira RJ, Marques-Cruz M, et al. metainc: assessment of inconsistency in meta-analysis using decision thresholds. Published online. 2024;0.2–1. doi: 10.32614/CRAN.package.metainc 39955079

[48] Sousa-Pinto B, Neumann I, Vieira RJ, et al. Quantitative assessment of inconsistency in meta-analysis using decision thresholds with two new indices. J Clin Epidemiol. 2025;181:111725. doi: 10.1016/j.jclinepi.2025.111725 39955079

[49] Harrer M, Cuijpers P, Furukawa TA, et al. Doing meta-analysis with R: a hands-on guide 1st ed. Chapman and Hall/CRC; 2021. doi: 10.1201/9781003107347

[50] John LI. Muscle glycogen synthesis before and after exercise. Sports Med. 1991;11(1):6–19. doi: 10.2165/00007256-199111010-00002 2011684

[51] Makowski D, Ben-Shachar M, Lüdecke D. bayestestR: describing effects and their uncertainty, existence and significance within the bayesian framework. J Open Source Softw. 2019;4(40):1541. doi: 10.21105/joss.01541

[52] Kass RE, Raftery AE. Bayes factors. J Am Stat Assoc. 1995;90:773–795. doi: 10.1080/01621459.1995.10476572

[53] Kruschke JK, Liddell TM. The bayesian new statistics: hypothesis testing, estimation, meta-analysis, and power analysis from a bayesian perspective. Psychon Bull Rev. 2018;25(1):178–206. doi: 10.3758/s13423-016-1221-4 28176294

[54] Gomaa MAE, Allam MG, Haridi AAIM, et al. High-protein concentrated pro-yogurt (Pro-WPI) enriched with whey protein isolate improved athletic anemia and performance in a placebo-controlled study. Front Nutr. 2022;8:788446. doi: 10.3389/fnut.2021.788446 35127786 PMC8811298

[55] Laskowski R, Antosiewicz J. Increased adaptability of young judo sportsmen after protein supplementation. J Sports Med Phys Fitness. 2003;43(3):342–346. 14625516

[56] Macdermid PW, Stannard SR. A whey-supplemented, high-protein diet versus a high-carbohydrate diet: effects on endurance cycling performance. Int J Sport Nutr Exerc Metab. 2006;16(1):65–77. doi: 10.1123/ijsnem.16.1.65 16676704

[57] Valenzuela PL, Alejo LB, Montalvo-Perez A, et al. Pre-sleep protein supplementation in professional cyclists during a training camp: a three-arm randomized controlled trial. J Int Soc Sports Nutr. 2023;20(1). doi: 10.1080/15502783.2023.2166366 PMC9848340 36686220

[58] Wolfe AS, Brandt SA, Krause IA, et al. Shorter duration time trial performance and recovery is not improved by inclusion of protein in a multiple carbohydrate supplement. J Strength Cond Res. 2017;31(9):2509–2518. doi: 10.1519/JSC.0000000000001733 27930452

[59] Hiroux C, Schouten M, De Glisezinski I, et al. Effect of increased protein intake and exogenous ketosis on body composition, energy expenditure and exercise capacity during a hypocaloric diet in recreational female athletes. Front Physiol. 2023;13:1063956. doi: 10.3389/fphys.2022.1063956 36714318 PMC9880233

[60] Mettler S, Mitchell N, Tipton KD. Increased protein intake reduces lean body mass loss during weight loss in athletes. Med Sci Sports Exerc. 2010;42(2):326–337. doi: 10.1249/MSS.0b013e3181b2ef8e 19927027

[61] Imanian B, Hemmatinafar M, Daryanoosh F, et al. The effect of probiotics and casein supplementation on aerobic capacity parameters of male soccer players. J Int Soc Sports Nutr. 2024;21(1):2382165. doi: 10.1080/15502783.2024.2382165 39039903 PMC11268215

[62] Ivy JL, Res PT, Sprague RC, et al. Effect of a carbohydrate-protein supplement on endurance performance during exercise of varying intensity. Int J Sport Nutr Exerc Metab. 2003;13(3):382–395. doi: 10.1123/ijsnem.13.3.382 14669937

[63] Jentjens RL, van Loon LJ, Mann CH, et al. Addition of protein and amino acids to carbohydrates does not enhance postexercise muscle glycogen synthesis. J Appl Physiol Bethesda Md 1985. 2001;91(2):839–846. doi: 10.1152/jappl.2001.91.2.839 11457801

[64] Röhling M, McCarthy D, Berg A. Continuous protein supplementation reduces acute exercise-induced stress markers in athletes performing marathon. Nutrients. 2021;13(9):2929. doi: 10.3390/nu13092929 34578807 PMC8472015

[65] Saunders MJ, Luden ND, Herrick JE. Consumption of an oral carbohydrate-protein gel improves cycling endurance and prevents postexercise muscle damage. J Strength Cond Res. 2007;21(3):678–684. doi: 10.1519/r-20506.1 17685703

[66] Saunders MJ, Moore RW, Kies AK, et al. Carbohydrate and protein hydrolysate coingestion’s improvement of late-exercise time-trial performance. Int J Sport Nutr Exerc Metab. 2009;19(2):136–149. doi: 10.1123/ijsnem.19.2.136 19478339

[67] Schroer AB, Saunders MJ, Baur DA, et al. Cycling time trial performance may be impaired by whey protein and l-alanine intake during prolonged exercise. Int J Sport Nutr Exerc Metab. 2014;24(5):507–515. doi: 10.1123/ijsnem.2013-0173 24937205

[68] Van Essen M, Gibala MJ. Failure of protein to improve time trial performance when added to a sports drink. Med Sci Sports Exerc. 2006;38(8):1476–1483. doi: 10.1249/01.mss.0000228958.82968.0a 16888462

[69] Vegge G, Rønnestad BR, Ellefsen S. Improved cycling performance with ingestion of hydrolyzed marine protein depends on performance level. J Int Soc Sports Nutr. 2012;9(1):14. doi: 10.1186/1550-2783-9-14 22490509 PMC3349497

[70] Obradović J, Vukadinović Jurišić M, Rakonjac D. The effects of leucine and whey protein supplementation with eight weeks of resistance training on strength and body composition. J Sports Med Phys Fitness. 2020;60(6):864–869. doi: 10.23736/S0022-4707.20.09742-X 32118385

[71] Hida A, Hasegawa Y, Mekata Y, et al. Effects of egg white protein supplementation on muscle strength and serum free amino acid concentrations. Nutrients. 2012;4(10):1504–1517. doi: 10.3390/nu4101504 23201768 PMC3497008

[72] Hoffman JR, Ratamess NA, Tranchina CP, et al. Effect of a proprietary protein supplement on recovery indices following resistance exercise in strength/power athletes. Amino Acids. 2010;38(3):771–778. doi: 10.1007/s00726-009-0283-2 19347247

[73] Campbell BI, Aguilar D, Conlin L, et al. Effects of high versus low protein intake on body composition and maximal strength in aspiring female physique athletes engaging in an 8-week resistance training program. Int J Sport Nutr Exerc Metab. 2018;28(6):580–585. doi: 10.1123/ijsnem.2017-0389 29405780

[74] Grubic TJ, Sowinski RJ, Nevares BE, et al. Comparison of ingesting a food bar containing whey protein and isomalto-oligosaccharides to carbohydrate on performance and recovery from an acute bout of resistance-exercise and sprint conditioning: an open label, randomized, counterbalanced, crossover pilot study. J Int Soc Sports Nutr. 2019;16(1):34. doi: 10.1186/s12970-019-0301-z 31409363 PMC6693099

[75] Naclerio F, Larumbe-Zabala E, Larrosa M, et al. Intake of animal protein blend plus carbohydrate improves body composition with no impact on performance in endurance athletes. Int J Sport Nutr Exerc Metab. 2019;29(5):474–480. doi: 10.1123/ijsnem.2018-0359 30676135

[76] Goh Q, Boop CA, Luden ND, et al. Recovery from cycling exercise: effects of carbohydrate and protein beverages. Nutrients. 2012;4(7):568–584. doi: 10.3390/nu4070568 22852050 PMC3407981

[77] Rowlands DS, Thorp RM, Rossler K, et al. Effect of protein-rich feeding on recovery after intense exercise. Int J Sport Nutr Exerc Metab. 2007;17(6):521–543. doi: 10.1123/ijsnem.17.6.521 18156659

[78] Rowlands DS, Rössler K, Thorp RM, et al. Effect of dietary protein content during recovery from high-intensity cycling on subsequent performance and markers of stress, inflammation, and muscle damage in well-trained men. Appl Physiol Nutr Metab. 2008;33(1):39–51. doi: 10.1139/H07-136 18347652

[79] Furber M, Pyle S, Roberts M, et al. Comparing acute, high dietary protein and carbohydrate intake on transcriptional biomarkers, fuel utilisation and exercise performance in trained male runners. Nutrients. 2021;13(12):4391. doi: 10.3390/nu13124391 34959943 PMC8706924

[80] Bourrilhon C, Lepers R, Philippe M, et al. Influence of protein- versus carbohydrate-enriched feedings on physiological responses during an ultraendurance climbing race. Horm Metab Res Horm Stoffwechselforschung Horm Metab. 2010;42(1):31–37. doi: 10.1055/s-0029-1237727 19821225

[81] Rowlands DS, Wadsworth DP. Effect of high-protein feeding on performance and nitrogen balance in female cyclists. Med Sci Sports Exerc. 2011;43(1):44–53. doi: 10.1249/MSS.0b013e3181e93316 20508536

[82] Portier H, Chatard JC, Filaire E, et al. Effects of branched-chain amino acids supplementation on physiological and psychological performance during an offshore sailing race. Eur J Appl Physiol. 2008;104(5):787–794. doi: 10.1007/s00421-008-0832-5 18704484

[83] Poulios A, Georgakouli K, Draganidis D, et al. Protein-based supplementation to enhance recovery in team sports: what is the evidence? J Sports Sci Med. 2019;18(3):523–536. 31427875 PMC6683614

[84] Hill KM, Stathis CG, Grinfeld E, et al. Co-ingestion of carbohydrate and whey protein isolates enhance PGC-1α mRNA expression: a randomised, single blind, cross over study. J Int Soc Sports Nutr. 2013;10(1):8. doi: 10.1186/1550-2783-10-8 23402493 PMC3577454

[85] Naclerio F, Seijo M, Larumbe-Zabala E, et al. Effects of supplementation with beef or whey protein versus carbohydrate in master triathletes. J Am Coll Nutr. 2017;36(8):593–601. doi: 10.1080/07315724.2017.1335248 28910233

[86] Lunn WR, Pasiakos SM, Colletto MR, et al. Chocolate milk and endurance exercise recovery: protein balance, glycogen, and performance. Med Sci Sports Exerc. 2012;44(4):682–691. doi: 10.1249/MSS.0b013e3182364162 21904247

[87] Rankin P, Landy A, Stevenson E, et al. Milk: an effective recovery drink for female athletes. Nutrients. 2018;10(2):228. doi: 10.3390/nu10020228 29462969 PMC5852804

[88] Rankin P, Lawlor MJ, Hills FA, et al. The effect of milk on recovery from repeat-sprint cycling in female team-sport athletes. Appl Physiol Nutr Metab. 2018;43(2):113–122. doi: 10.1139/apnm-2017-0275 28972854

[89] Cepero González M, Rojas Ruiz FJ, Geerlings A, et al. Effects of a carbohydrate and a carbohydrate and casein protein beverages on recovery and performance of endurance cycling capacity. J Hum Sport Exerc. 2009;4(2):161–172. doi: 10.4100/jhse.2009.42.09

[90] Goldstein ER, Stout JR, Wells AJ, et al. Carbohydrate-protein drink is effective for restoring endurance capacity in masters class athletes after a two-hour recovery. J Int Soc Sports Nutr. 2023;20(1):2178858. doi: 10.1080/15502783.2023.2178858 36843067 PMC9970201

[91] Ferguson-Stegall L, McCleave EL, Ding Z, et al. Postexercise carbohydrate–protein supplementation improves subsequent exercise performance and intracellular signaling for protein synthesis. J Strength Cond Res. 2011;25(5):1210–1224. doi: 10.1519/JSC.0b013e318212db21 21522069

[92] Martínez-Lagunas V, Ding Z, Bernard JR, et al. Added protein maintains efficacy of a low-carbohydrate sports drink. J Strength Cond Res. 2010;24(1):48–59. doi: 10.1519/JSC.0b013e3181c32e20 19924010

[93] Eddens L, Browne S, Stevenson EJ, et al. The efficacy of protein supplementation during recovery from muscle-damaging concurrent exercise. Appl Physiol Nutr Metab. 2017;42(7):716–724. doi: 10.1139/apnm-2016-0626 28199799

[94] Röhling M, McCarthy D, Berg A. Continuous protein supplementation reduces acute exercise-induced stress markers in athletes performing marathon. Nutrients. 2021;13(9):2929. doi: 10.3390/nu13092929 34578807 PMC8472015

[95] Naclerio F, Larumbe-Zabala E, Larrosa M, et al. Intake of animal protein blend plus carbohydrate improves body composition with no impact on performance in endurance athletes. Int J Sport Nutr Exerc Metab. 2019;29(5):474–480. doi: 10.1123/ijsnem.2018-0359 30676135

[96] Finger D, Lanferdini FJ, Farinha JB, et al. Ingestion of carbohydrate or carbohydrate plus protein does not enhance performance during endurance exercise: a randomized crossover placebo-controlled clinical trial. Appl Physiol Nutr Metab. 2018;43(9):937–944. doi: 10.1139/apnm-2017-0835 29544062

[97] McCartney D, Desbrow B, Irwin C. Post-exercise ingestion of carbohydrate, protein and water: a systematic review and meta-analysis for effects on subsequent athletic performance. Sports Med. 2018;48(2):379–408. doi: 10.1007/s40279-017-0800-5 29098657

[98] Nelson AR, Phillips SM, Stellingwerff T, et al. A protein–leucine supplement increases branched-chain amino acid and nitrogen turnover but not performance. Med Sci Sports Exerc. 2012;44(1):57–68. doi: 10.1249/MSS.0b013e3182290371 21685813

[99] Setiawan MI, Susanto H, Kartasurya MI. Milk protein consumption improves muscle performance and total antioxidant status in young soccer athletes: a randomized controlled trial. Med J Indones. 2020;29(2):164–171. doi: 10.13181/mji.oa.202872

[100] Colombani PC, Mannhart C, Mettler S. Carbohydrates and exercise performance in non-fasted athletes: a systematic review of studies mimicking real-life. Nutr J. 2013;12(1):16. doi: 10.1186/1475-2891-12-16 23356905 PMC3570376

[101] Nunes EA, Colenso‐Semple L, McKellar SR, et al. Systematic review and meta‐analysis of protein intake to support muscle mass and function in healthy adults. J Cachexia Sarcopenia Muscle. 2022;13(2):795–810. doi: 10.1002/jcsm.12922 35187864 PMC8978023

[102] Hartono FA, Martin-Arrowsmith PW, Peeters WM, et al. The effects of dietary protein supplementation on acute changes in muscle protein synthesis and longer-term changes in muscle mass, strength, and aerobic capacity in response to concurrent resistance and endurance exercise in healthy adults: a systematic review. Sports Med. 2022;52(6):1295–1328. doi: 10.1007/s40279-021-01620-9 35113389

[103] Staples AW, Burd NA, West DWD, et al. Carbohydrate does not augment exercise-induced protein accretion versus protein alone. Med Sci Sports Exerc. 2011;43(7):1154–1161. doi: 10.1249/MSS.0b013e31820751cb 21131864

[104] Zare R, Devrim-Lanpir A, Guazzotti S, et al. Effect of soy protein supplementation on muscle adaptations, metabolic and antioxidant status, hormonal response, and exercise performance of active individuals and athletes: a systematic review of randomised controlled trials. Sports Med. 2023;53(12):2417–2446. doi: 10.1007/s40279-023-01899-w 37603200 PMC10687132

[105] Morton RW, Murphy KT, McKellar SR, et al. A systematic review, meta-analysis and meta-regression of the effect of protein supplementation on resistance training-induced gains in muscle mass and strength in healthy adults. Br J Sports Med. 2018;52(6):376–384. doi: 10.1136/bjsports-2017-097608 28698222 PMC5867436

[106] Williamson E, Fung HJW, Adams C, et al. Protein requirements are increased in endurance-trained athletes but similar between females and males during postexercise recovery. Med Sci Sports Exerc. 2023;55(10):1866–1875. doi: 10.1249/MSS.0000000000003219 37710376

[107] Mercer D, Convit L, Condo D, et al. Protein requirements of pre-menopausal female athletes: systematic literature review. Nutrients. 2020;12(11):3527. doi: 10.3390/nu12113527 33207749 PMC7696053

[108] Moore DR. Maximizing post-exercise anabolism: the case for relative protein intakes. Front Nutr. 2019;6:147. doi: 10.3389/fnut.2019.00147 31552263 PMC6746967

[109] Moore DR, Sygo J, Morton JP. Fuelling the female athlete: carbohydrate and protein recommendations. Eur J Sport Sci. 2022;22(5):684–696. doi: 10.1080/17461391.2021.1922508 34015236

[110] Williams MB, Raven PB, Fogt DL, et al. Effects of recovery beverages on glycogen restoration and endurance exercise performance. J Strength Cond Res. 2003;17(1):12–19. 12580650 10.1519/1533-4287(2003)017<0012:eorbog>2.0.co;2

[111] Mhamed MB, Zarrouk F, Mrad M, et al. Effects of whey protein on body composition, biochemical profile, and high intensity physical performances in well-trained endurance runners. Sci Sports. 2024;39(7):588–598. doi: 10.1016/j.scispo.2024.02.001

[112] Pasiakos SM, Lieberman HR, McLellan TM. Effects of protein supplements on muscle damage, soreness and recovery of muscle function and physical performance: a systematic review. Sports Med. 2014;44(5):655–670. doi: 10.1007/s40279-013-0137-7 24435468

[113] Rowlands DS, Rössler K, Thorp RM, et al. Effect of dietary protein content during recovery from high-intensity cycling on subsequent performance and markers of stress, inflammation, and muscle damage in well-trained men. Appl Physiol Nutr Metab. 2008;33(1):39–51. doi: 10.1139/H07-136 18347652

[114] Valentine RJ, Saunders MJ, Todd MK, et al. Influence of carbohydrate-protein beverage on cycling endurance and indices of muscle disruption. Int J Sport Nutr Exerc Metab. 2008;18(4):363–378. doi: 10.1123/ijsnem.18.4.363 18708686

[115] Breen L, Tipton KD, Jeukendrup AE. No effect of carbohydrate-protein on cycling performance and indices of recovery. Med Sci Sports Exerc. 2010;42(6):1140–1148. doi: 10.1249/MSS.0b013e3181c91f1a 19997018

[116] Cermak NM, Solheim AS, Gardner MS, et al. Muscle metabolism during exercise with carbohydrate or protein-carbohydrate ingestion. Med Sci Sports Exerc. 2009;41(12):2158–2164. doi: 10.1249/MSS.0b013e3181ac10bf 19915503

