# Supplementary material for: The effectiveness of protein supplements on athletic performance and post-exercise recovery − a Bayesian multilevel meta-analysis of randomized controlled trials
Source: J Int Soc Sports Nutr. 2025 Dec 23;23(1):2605338. doi: 10.1080/15502783.2025.2605338 (PMC12777903; doi:10.1080/15502783.2025.2605338)
Supplement: supplementary material — Supplementary_file_S12_The_included_literature_in_meta_analysis. [file RSSN_A_2605338_SM7140.docx]

**Supplementary file S12: The Included Literature in Meta-analysis**

1. Goh Q, Boop CA, Luden ND, Smith AG, Womack CJ, Saunders MJ. Recovery from Cycling Exercise: Effects of Carbohydrate and Protein Beverages. *Nutrients*. 2012;4(7):568-584. doi:10.3390/nu4070568

2. Hiroux C, Schouten M, De Glisezinski I, et al. Effect of increased protein intake and exogenous ketosis on body composition, energy expenditure and exercise capacity during a hypocaloric diet in recreational female athletes. *Front Physiol*. 2023;13:1063956. doi:10.3389/fphys.2022.1063956

3. Fabre M, Mathieu B, Tiollier E, et al. Effects of Native Whey Protein and Carbohydrate Supplement on Physical Performance and Plasma Markers of Muscle Damage and Inflammation during a Simulated Rugby Sevens Tournament: A Double-Blind, Randomized, Placebo-Controlled, Crossover Study. *Nutrients*. 2022;14(22):4780. doi:10.3390/nu14224780

4. Mourier A, Bigard A, Kerviler ED, Roger B, Legrand H, Guezennec C. Combined Effects of Caloric Restriction and Branched-Chain Amino Acid Supplementation on Body Composition and Exercise Performance in Elite Wrestlers. *Int J Sports Med*. 1997;18(01):47-55. doi:10.1055/s-2007-972594

5. Cepero González M, Rojas Ruiz FJ, Geerlings A, Cruz Márquez JCDL, Romero Granados S, Boza Puerta JJ. Effects of a carbohydrate and a carbohydrate and casein protein beverages on recovery and performance of endurance cycling capacity. *jhse*. 2009;4(2):161-172. doi:10.4100/jhse.2009.42.09

6. Cogan KE, Evans M, Iuliano E, et al. Co-ingestion of protein or a protein hydrolysate with carbohydrate enhances anabolic signaling, but not glycogen resynthesis, following recovery from prolonged aerobic exercise in trained cyclists. *Eur J Appl Physiol*. 2018;118(2):349-359. doi:10.1007/s00421-017-3775-x

7. Shenoy S, Dhawan M, Singh Sandhu J. Four Weeks of Supplementation With Isolated Soy Protein Attenuates Exercise-Induced Muscle Damage and Enhances Muscle Recovery in Well Trained Athletes: A Randomized Trial. *Asian J Sports Med*. 2016;7(3). doi:10.5812/asjsm.33528

8. Hansen M, Bangsbo J, Jensen J, Bibby BM, Madsen K. Effect of Whey Protein Hydrolysate on Performance and Recovery of Top-Class Orienteering Runners. *International Journal of Sport Nutrition and Exercise Metabolism*. 2015;25(2):97-109. doi:10.1123/ijsnem.2014-0083

9. Miles KH, Clark B, Fowler PM, et al. ɑ-Lactalbumin Improves Sleep and Recovery after Simulated Evening Competition in Female Athletes. *Medicine & Science in Sports & Exercise*. 2021;53(12):2618-2627. doi:10.1249/MSS.0000000000002743

10. Obradović J, Vukadinović Jurišić M, Rakonjac D. The effects of leucine and whey protein supplementation with eight weeks of resistance training on strength and body composition. *J Sports Med Phys Fitness*. 2020;60(6). doi:10.23736/S0022-4707.20.09742-X

11. Gomaa MAE, Allam MG, Haridi AAIM, Eliwa AEM, Darwish AMG. High-Protein Concentrated Pro-Yogurt (Pro-WPI) Enriched With Whey Protein Isolate Improved Athletic Anemia and Performance in a Placebo-Controlled Study. *Front Nutr*. 2022;8:788446. doi:10.3389/fnut.2021.788446

12. Rankin P, Lawlor MJ, Hills FA, Bell PG, Stevenson EJ, Cockburn E. The effect of milk on recovery from repeat-sprint cycling in female team-sport athletes. *Appl Physiol Nutr Metab*. 2018;43(2):113-122. doi:10.1139/apnm-2017-0275

13. Goldstein ER, Stout JR, Wells AJ, Antonio J, Vasenina E, Fukuda DH. Carbohydrate-Protein drink is effective for restoring endurance capacity in masters class athletes after a two-Hour recovery. *Journal of the International Society of Sports Nutrition*. 2023;20(1):2178858. doi:10.1080/15502783.2023.2178858

14. Mhamed MB, Zarrouk F, Mrad M, et al. Effects of whey protein on body composition, biochemical profile, and high intensity physical performances in well-trained endurance runners. *Science & Sports*. 2024;39(7):588-598. doi:10.1016/j.scispo.2024.02.001

15. Sollie O, Clauss M, Jeppesen PB, et al. Similar performance after intake of carbohydrate plus whey protein and carbohydrate only in the early phase after non‐exhaustive cycling. *Scandinavian Med Sci Sports*. 2023;33(7):1091-1103. doi:10.1111/sms.14364

16. Rankin P, Stevenson E, Cockburn E. The effect of milk on the attenuation of exercise-induced muscle damage in males and females. *Eur J Appl Physiol*. 2015;115(6):1245-1261. doi:10.1007/s00421-015-3121-0

17. Imanian B, Hemmatinafar M, Daryanoosh F, et al. The effect of probiotics and casein supplementation on aerobic capacity parameters of male soccer players. *Journal of the International Society of Sports Nutrition*. 2024;21(1):2382165. doi:10.1080/15502783.2024.2382165

18. Lee C. Psyching Up for a Muscular Endurance Task: Effects of Image Content on Performance and Mood State. *Journal of Sport and Exercise Psychology*. 1990;12(1):66-73. doi:10.1123/jsep.12.1.66

19. Larsen MS, Clausen D, Jørgensen AA, Mikkelsen UR, Hansen M. Presleep Protein Supplementation Does Not Improve Recovery During Consecutive Days of Intense Endurance Training: A Randomized Controlled Trial. *International Journal of Sport Nutrition and Exercise Metabolism*. 2019;29(4):426-434. doi:10.1123/ijsnem.2018-0286

20. Taylor LW, Wilborn C, Roberts MD, White A, Dugan K. Eight weeks of pre- and postexercise whey protein supplementation increases lean body mass and improves performance in Division III collegiate female basketball players. *Appl Physiol Nutr Metab*. 2016;41(3):249-254. doi:10.1139/apnm-2015-0463

21. Ferguson-Stegall L, McCleave EL, Ding Z, et al. The Effect of a Low Carbohydrate Beverage with Added Protein on Cycling Endurance Performance in Trained Athletes. *Journal of Strength and Conditioning Research*. 2010;24(10):2577-2586. doi:10.1519/JSC.0b013e3181ecccca

22. Cepero González M, Padial R, Rojas Ruiz FJ, Geerlings A, Cruz Márquez JCDL, Boza Puerta JJ. Influence of ingesting casein protein and whey protein carbohydrate beverages on recovery and performance of an endurance cycling test. *jhse*. 2010;5(2):158-175. doi:10.4100/jhse.2010.52.06

23. Hida A, Hasegawa Y, Mekata Y, et al. Effects of Egg White Protein Supplementation on Muscle Strength and Serum Free Amino Acid Concentrations. *Nutrients*. 2012;4(10):1504-1517. doi:10.3390/nu4101504

24. Setiawan MI, Susanto H, Kartasurya MI. Milk protein consumption improves muscle performance and total antioxidant status in young soccer athletes: a randomized controlled trial. *Med J Indones*. 2020;29(2):164-171. doi:10.13181/mji.oa.202872

25. Ferguson-Stegall L, McCleave EL, Ding Z, et al. Postexercise Carbohydrate–Protein Supplementation Improves Subsequent Exercise Performance and Intracellular Signaling for Protein Synthesis. *Journal of Strength and Conditioning Research*. 2011;25(5):1210-1224. doi:10.1519/JSC.0b013e318212db21

26. Hill KM, Stathis CG, Grinfeld E, Hayes A, McAinch AJ. Co-ingestion of carbohydrate and whey protein isolates enhance PGC-1α mRNA expression: a randomised, single blind, cross over study. *Journal of the International Society of Sports Nutrition*. 2013;10(1):8. doi:10.1186/1550-2783-10-8

27. Dow K, Pritchett R, Roemer K, Pritchett K. Chocolate Milk as a Post-Exercise Recovery Aid in Division II Collegiate Volleyball Players. *Women in Sport and Physical Activity Journal*. 2019;27(1):45-51. doi:10.1123/wspaj.2018-0012

28. Romano-Ely BC, Todd MK, Saunders MJ, St.Laurent T. Effect of an Isocaloric Carbohydrate-Protein-Antioxidant Drink on Cycling Performance. *Medicine & Science in Sports & Exercise*. 2006;38(9):1608-1616. doi:10.1249/01.mss.0000229458.11452.e9

29. Oosthuyse T, Carstens M, Millen A. Whey or Casein Hydrolysate with Carbohydrate for Metabolism and Performance in Cycling. *Int J Sports Med*. 2015;36(08):636-646. doi:10.1055/s-0034-1398647

30. Rankin P, Landy A, Stevenson E, Cockburn E. Milk: An Effective Recovery Drink for Female Athletes. *Nutrients*. 2018;10(2):228. doi:10.3390/nu10020228

31. Wolfe AS, Brandt SA, Krause IA, Mavison RW, Aponte JA, Ferguson-Stegall LM. Shorter Duration Time Trial Performance and Recovery Is Not Improved by Inclusion of Protein in a Multiple Carbohydrate Supplement. *Journal of Strength and Conditioning Research*. 2017;31(9):2509-2518. doi:10.1519/JSC.0000000000001733

32. Hoffman JR, Ratamess NA, Tranchina CP, Rashti SL, Kang J, Faigenbaum AD. Effect of a proprietary protein supplement on recovery indices following resistance exercise in strength/power athletes. *Amino Acids*. 2010;38(3):771-778. doi:10.1007/s00726-009-0283-2

33. Poulios A, Georgakouli K, Draganidis D, et al. Protein-Based Supplementation to Enhance Recovery in Team Sports: What is the Evidence? *Journal of Sports Science & Medicine*. 2019;18(3):523-536.

34. Abbott W, Brett A, Cockburn E, Clifford T. Presleep Casein Protein Ingestion: Acceleration of Functional Recovery in Professional Soccer Players. *International Journal of Sports Physiology and Performance*. 2019;14(3):385-391. doi:10.1123/ijspp.2018-0385

35. Martínez-Lagunas V, Ding Z, Bernard JR, Wang B, Ivy JL. Added Protein Maintains Efficacy of a Low-Carbohydrate Sports Drink. *Journal of Strength and Conditioning Research*. 2010;24(1):48-59. doi:10.1519/JSC.0b013e3181c32e20

36. Kirk B, Mitchell J, Jackson M, Amirabdollahian F, Alizadehkhaiyat O, Clifford T. A2 Milk Enhances Dynamic Muscle Function Following Repeated Sprint Exercise, a Possible Ergogenic Aid for A1-Protein Intolerant Athletes? *Nutrients*. 2017;9(2):94. doi:10.3390/nu9020094

37. Huang WC, Chang YC, Chen YM, et al. Whey Protein Improves Marathon-Induced Injury and Exercise Performance in Elite Track Runners. *Int J Med Sci*. 2017;14(7):648-654. doi:10.7150/ijms.19584

38. Eddens L, Browne S, Stevenson EJ, Sanderson B, Van Someren K, Howatson G. The efficacy of protein supplementation during recovery from muscle-damaging concurrent exercise. *Appl Physiol Nutr Metab*. 2017;42(7):716-724. doi:10.1139/apnm-2016-0626

39. Nelson AR, Phillips SM, Stellingwerff T, et al. A Protein–Leucine Supplement Increases Branched-Chain Amino Acid and Nitrogen Turnover But Not Performance. *Medicine & Science in Sports & Exercise*. 2012;44(1):57-68. doi:10.1249/MSS.0b013e3182290371

40. Vegge G, Rønnestad BR, Ellefsen S. Improved cycling performance with ingestion of hydrolyzed marine protein depends on performance level. *Journal of the International Society of Sports Nutrition*. 2012;9(1):14. doi:10.1186/1550-2783-9-14

41. Van Essen M, Gibala MJ. Failure of Protein to Improve Time Trial Performance when Added to a Sports Drink. *Medicine & Science in Sports & Exercise*. 2006;38(8):1476-1483. doi:10.1249/01.mss.0000228958.82968.0a

42. Saunders MJ, Kane MD, Todd MK. Effects of a Carbohydrate-Protein Beverage on Cycling Endurance and Muscle Damage: *Medicine & Science in Sports & Exercise*. 2004;36(7):1233-1238. doi:10.1249/01.MSS.0000132377.66177.9F

43. Saunders MJ, Moore RW, Kies AK, Luden ND, Pratt CA. Carbohydrate and Protein Hydrolysate Coingestion’s Improvement of Late-Exercise Time-Trial Performance. *International Journal of Sport Nutrition and Exercise Metabolism*. 2009;19(2):136-149. doi:10.1123/ijsnem.19.2.136

44. Lunn WR, Pasiakos SM, Colletto MR, et al. Chocolate Milk and Endurance Exercise Recovery: Protein Balance, Glycogen, and Performance. *Medicine & Science in Sports & Exercise*. 2012;44(4):682-691. doi:10.1249/MSS.0b013e3182364162

45. Ivy JL, Res PT, Sprague RC, Widzer MO. Effect of a Carbohydrate-Protein Supplement on Endurance Performance during Exercise of Varying Intensity. *International Journal of Sport Nutrition and Exercise Metabolism*. 2003;13(3):382-395. doi:10.1123/ijsnem.13.3.382

46. Rowlands DS, Rössler K, Thorp RM, et al. Effect of dietary protein content during recovery from high-intensity cycling on subsequent performance and markers of stress, inflammation, and muscle damage in well-trained men. *Appl Physiol Nutr Metab*. 2008;33(1):39-51. doi:10.1139/H07-136

47. Rowlands DS, Thorp RM, Rossler K, Graham DF, Rockell MJ. Effect of Protein-Rich Feeding on Recovery after Intense Exercise. *International Journal of Sport Nutrition and Exercise Metabolism*. 2007;17(6):521-543. doi:10.1123/ijsnem.17.6.521

48. Gilson SF, Saunders MJ, Moran CW, Moore RW, Womack CJ, Todd MK. Effects of chocolate milk consumption on markers of muscle recovery following soccer training: a randomized cross-over study. *Journal of the International Society of Sports Nutrition*. 2010;7(1):19. doi:10.1186/1550-2783-7-19

49. Alghannam AF, Jedrzejewski D, Bilzon J, Thompson D, Tsintzas K, Betts JA. Influence of Post-Exercise Carbohydrate-Protein Ingestion on Muscle Glycogen Metabolism in Recovery and Subsequent Running Exercise. *International Journal of Sport Nutrition and Exercise Metabolism*. 2016;26(6):572-580. doi:10.1123/ijsnem.2016-0021

50. Jentjens RL, van Loon LJ, Mann CH, Wagenmakers AJ, Jeukendrup AE. Addition of protein and amino acids to carbohydrates does not enhance postexercise muscle glycogen synthesis. *J Appl Physiol (1985)*. 2001;91(2):839-846. doi:10.1152/jappl.2001.91.2.839

51. Hall AH, Leveritt MD, Ahuja KD k, Shing CM. Coingestion of carbohydrate and protein during training reduces training stress and enhances subsequent exercise performance. *Applied Physiology, Nutrition & Metabolism*. 2013;38(12):597-604. doi:10.1139/apnm-2012-0281

52. Furber M, Pyle S, Roberts M, Roberts J. Comparing Acute, High Dietary Protein and Carbohydrate Intake on Transcriptional Biomarkers, Fuel Utilisation and Exercise Performance in Trained Male Runners. *NUTRIENTS*. 2021;13(12). doi:10.3390/nu13124391

53. Saunders MJ, Luden ND, Herrick JE. CONSUMPTION OF AN ORAL CARBOHYDRATE-PROTEIN GEL IMPROVES CYCLING ENDURANCE AND PREVENTS POSTEXERCISE MUSCLE DAMAGE. *Journal of Strength & Conditioning Research*. 2007;21(3):678-684. doi:10.1519/r-20506.1

54. Bourrilhon C, Lepers R, Philippe M, et al. Influence of protein- versus carbohydrate-enriched feedings on physiological responses during an ultraendurance climbing race. *Horm Metab Res*. 2010;42(1):31-37. doi:10.1055/s-0029-1237727

55. Finger D, Lanferdini FJ, Farinha JB, et al. Ingestion of carbohydrate or carbohydrate plus protein does not enhance performance during endurance exercise: a randomized crossover placebo-controlled clinical trial. *Applied Physiology, Nutrition & Metabolism*. 2018;43(9):937-944. doi:10.1139/apnm-2017-0835

56. Toone RJ, Betts JA. Isocaloric carbohydrate versus carbohydrate-protein ingestion and cycling time-trial performance. *International Journal of Sport Nutrition & Exercise Metabolism*. 2010;20(1):34-43. doi:10.1123/ijsnem.20.1.34

57. Sollie O, Jeppesen PB, Tangen DS, et al. Protein intake in the early recovery period after exhaustive exercise improves performance the following day. *JOURNAL OF APPLIED PHYSIOLOGY*. 2018;125(6):1731-1742. doi:10.1152/japplphysiol.01132.2017

58. Ghosh AK, Rahaman AA, Singh R. Combination of sago and soy-protein supplementation during endurance cycling exercise and subsequent high-intensity endurance capacity. *INTERNATIONAL JOURNAL OF SPORT NUTRITION AND EXERCISE METABOLISM*. 2010;20(3):216-223. doi:10.1123/ijsnem.20.3.216

59. Campbell BI, Aguilar D, Conlin L, et al. Effects of High Versus Low Protein Intake on Body Composition and Maximal Strength in Aspiring Female Physique Athletes Engaging in an 8-Week Resistance Training Program. *International Journal of Sport Nutrition and Exercise Metabolism*. 2018;28(6):580-585. doi:10.1123/ijsnem.2017-0389

60. Schroer AB, Saunders MJ, Baur DA, Womack CJ, Luden ND. Cycling Time Trial Performance May Be Impaired by Whey Protein and L-Alanine Intake During Prolonged Exercise. *International Journal of Sport Nutrition & Exercise Metabolism*. 2014;24(5):507-515. doi:10.1123/ijsnem.2013-0173

61. Rowlands DS, Wadsworth DP. Effect of High-Protein Feeding on Performance and Nitrogen Balance in Female Cyclists. *Medicine & Science in Sports & Exercise*. 2011;43(1):44-53. doi:10.1249/MSS.0b013e3181e93316

62. Macdermid PW, Stannard SR. A whey-supplemented, high-protein diet versus a high-carbohydrate diet: effects on endurance cycling performance. *INTERNATIONAL JOURNAL OF SPORT NUTRITION AND EXERCISE METABOLISM*. 2006;16(1):65-77. doi:10.1123/ijsnem.16.1.65

63. Grubic TJ, Sowinski RJ, Nevares BE, et al. Comparison of ingesting a food bar containing whey protein and isomalto-oligosaccharides to carbohydrate on performance and recovery from an acute bout of resistance-exercise and sprint conditioning: an open label, randomized, counterbalanced, crossover pilot study. *JOURNAL OF THE INTERNATIONAL SOCIETY OF SPORTS NUTRITION*. 2019;16(1):34. doi:10.1186/s12970-019-0301-z

64. Kritikos S, Papanikolaou K, Draganidis D, et al. Effect of whey vs. soy protein supplementation on recovery kinetics following speed endurance training in competitive male soccer players: a randomized controlled trial. *JOURNAL OF THE INTERNATIONAL SOCIETY OF SPORTS NUTRITION*. 2021;18(1):23. doi:10.1186/s12970-021-00420-w

65. Valenzuela PL, Alejo LB, Montalvo-Perez A, et al. Pre-sleep protein supplementation in professional cyclists during a training camp: a three-arm randomized controlled trial. *JOURNAL OF THE INTERNATIONAL SOCIETY OF SPORTS NUTRITION*. 2023;20(1). doi:10.1080/15502783.2023.2166366

66. Hansen M, Bangsbo J, Jensen J, et al. Protein intake *during* training sessions has no effect on performance and recovery during a strenuous training camp for elite cyclists. *Journal of the International Society of Sports Nutrition*. 2016;13(1):9. doi:10.1186/s12970-016-0120-4

67. Mettler S, Mitchell N, Tipton KD. Increased protein intake reduces lean body mass loss during weight loss in athletes. *Medicine & Science in Sports & Exercise*. 2010;42(2):326-337. doi:10.1249/MSS.0b013e3181b2ef8e

68. Naclerio F, Larumbe-Zabala E, Larrosa M, Centeno A, Esteve-Lanao J, Moreno-Pérez D. Intake of Animal Protein Blend Plus Carbohydrate Improves Body Composition With no Impact on Performance in Endurance Athletes. *International Journal of Sport Nutrition & Exercise Metabolism*. 2019;29(5):474-480. doi:10.1123/ijsnem.2018-0359

69. Thomson JS, Ali A, Rowlands DS. Leucine-protein supplemented recovery feeding enhances subsequent cycling performance in well-trained men. *Applied Physiology, Nutrition & Metabolism*. 2011;36(2):242-253. doi:10.1139/h10-104

70. Naclerio F, Seijo M, Larumbe-Zabala E, et al. Effects of Supplementation with Beef or Whey Protein Versus Carbohydrate in Master Triathletes. *Journal of the American College of Nutrition*. 2017;36(8):593-601. doi:10.1080/07315724.2017.1335248

71. Portier H, Chatard JC, Filaire E, Jaunet-Devienne MF, Robert A, Guezennec CY. Effects of branched-chain amino acids supplementation on physiological and psychological performance during an offshore sailing race. *European Journal of Applied Physiology*. 2008;104(5):787-794. doi:10.1007/s00421-008-0832-5

72. Williams MB, Raven PB, Fogt DL, Ivy JL. Effects of recovery beverages on glycogen restoration and endurance exercise performance. *J Strength Cond Res*. 2003;17(1):12-19. doi:10.1519/1533-4287(2003)017<0012:eorbog>2.0.co;2

73. Highton J, Twist C, Lamb K, Nicholas C. Carbohydrate-protein coingestion improves multiple-sprint running performance. *Journal of Sports Sciences*. 2013;31(4):361-369. doi:10.1080/02640414.2012.735370

74. Röhling M, McCarthy D, Berg A. Continuous Protein Supplementation Reduces Acute Exercise-Induced Stress Markers in Athletes Performing Marathon. *Nutrients*. 2021;13(9):2929. doi:10.3390/nu13092929

75. Laskowski R. Increased adaptability of youth judo sportsmen after protein supplementation. *Journal of sports medicine and physical fitness*. 2003;43(3):342.
