# Supplementary figures and images for: The effectiveness of protein supplements on athletic performance and post-exercise recovery − a Bayesian multilevel meta-analysis of randomized controlled trials
Source: J Int Soc Sports Nutr. 2025 Dec 23;23(1):2605338. doi: 10.1080/15502783.2025.2605338 (PMC12777903; doi:10.1080/15502783.2025.2605338)

**Supplementary File S11: Graphical Abstract**


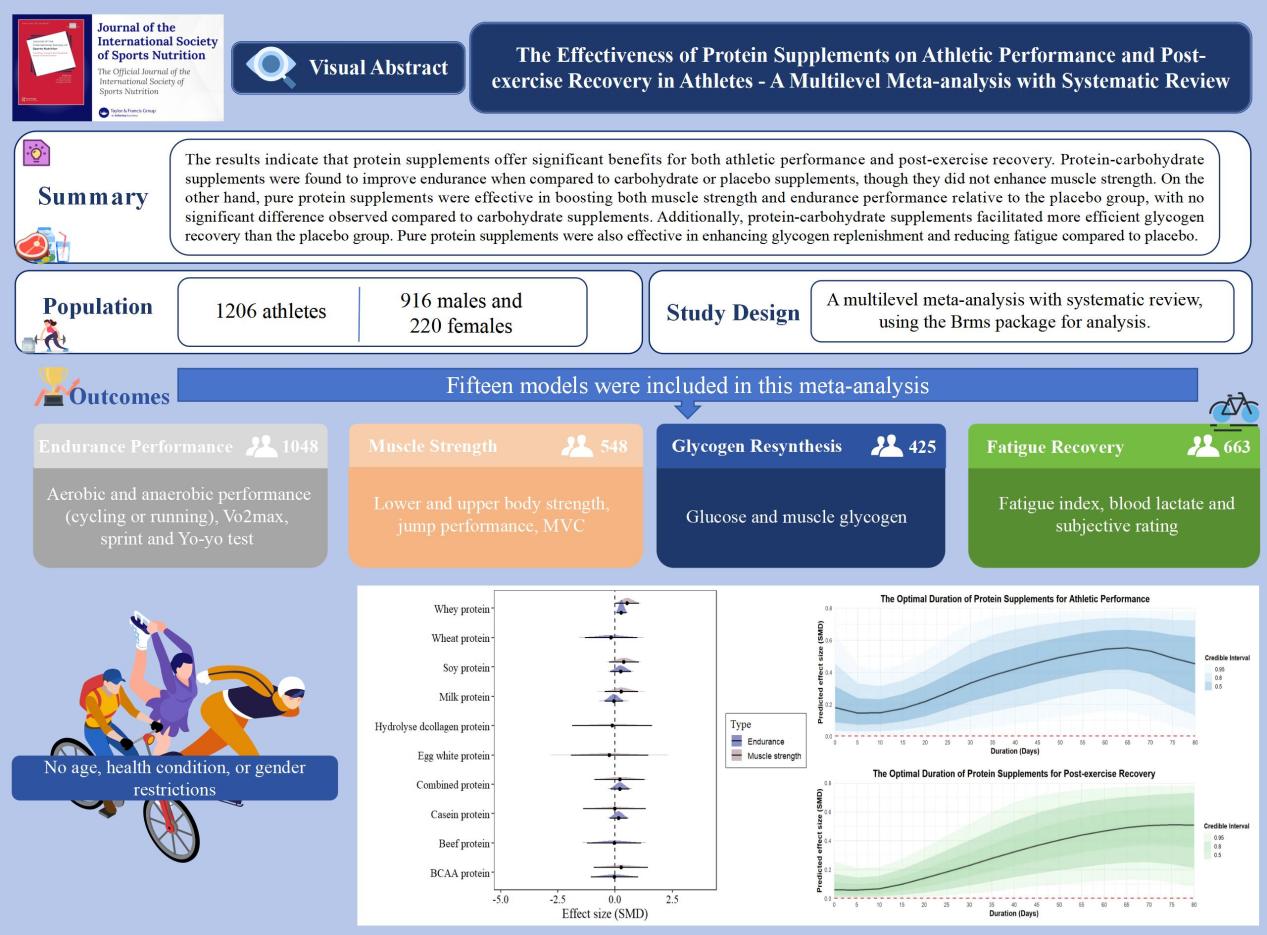


**Fig.S1** The Graphical Abstract

Supplement: supplementary material — Supplementary_file_S11_Graphical_Abstract. [file RSSN_A_2605338_SM6228.docx]
