# Supplementary material for: The effectiveness of protein supplements on athletic performance and post-exercise recovery − a Bayesian multilevel meta-analysis of randomized controlled trials
Source: J Int Soc Sports Nutr. 2025 Dec 23;23(1):2605338. doi: 10.1080/15502783.2025.2605338 (PMC12777903; doi:10.1080/15502783.2025.2605338)
Supplement: supplementary material — Supplementary_file_S10. [file RSSN_A_2605338_SM6216.docx]

**Supplementary File S10: Funnel Plots (Publicationbias vs. Metafor)**

**
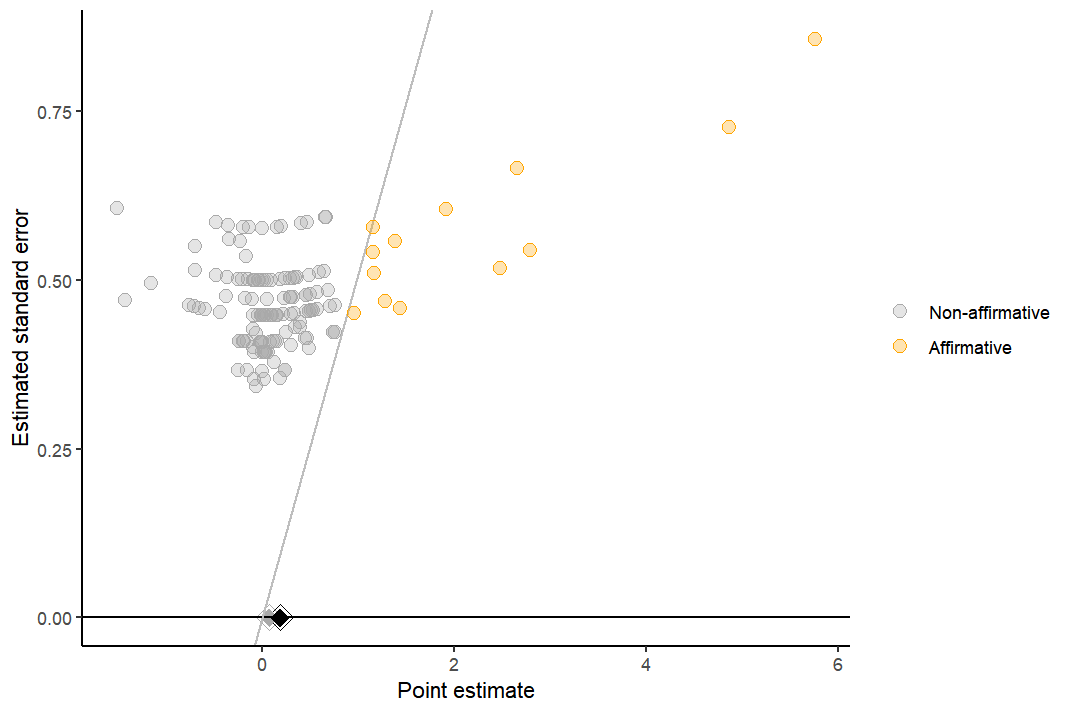
**

**Fig.S1** The Funnel Plot via PublicationBias Package (Endurance)


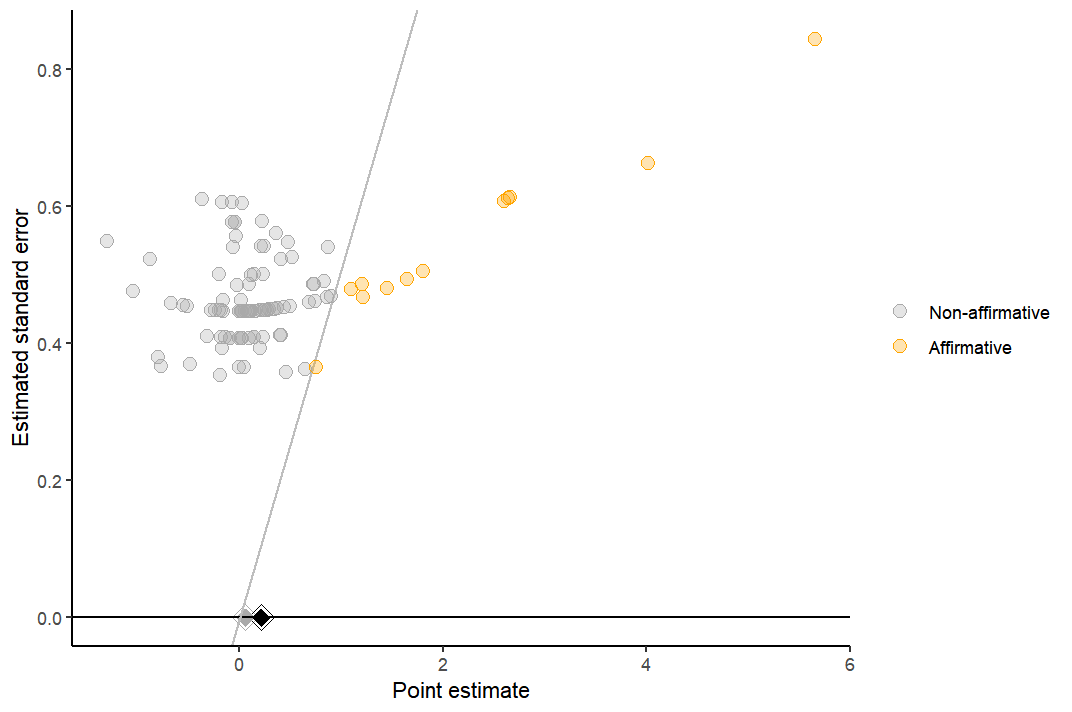


**Fig.S2** The Funnel Plot via PublicationBias Package (Muscle Strength)


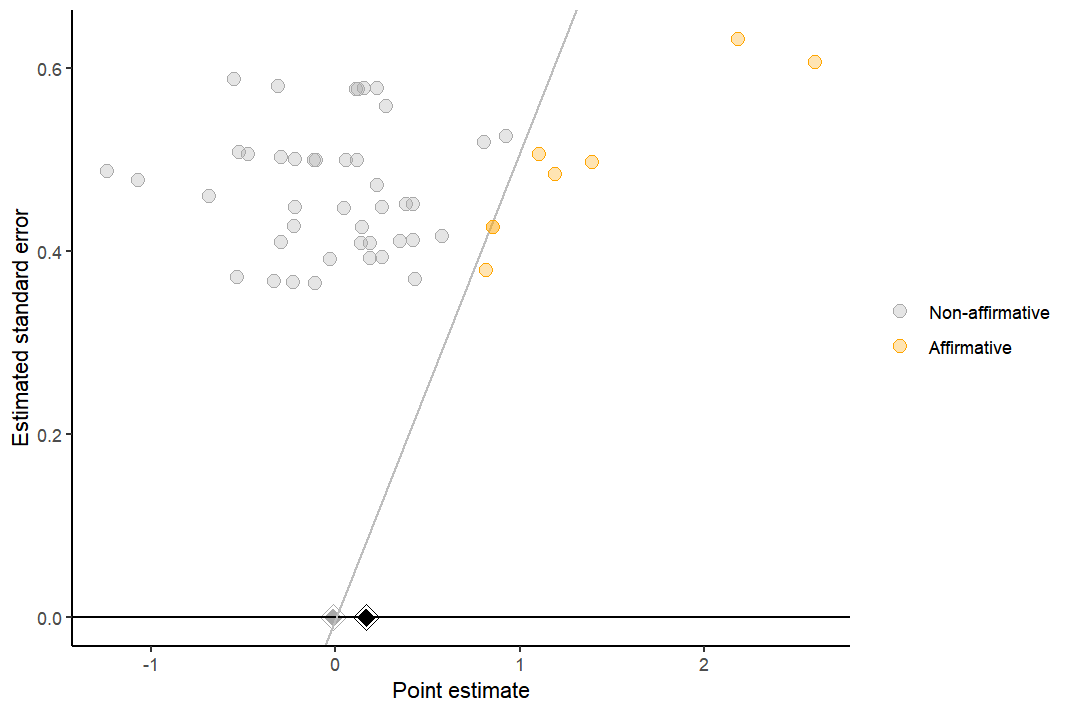


**Fig.S3** The Funnel Plot via PublicationBias Package (Glycogen Resynthesis)


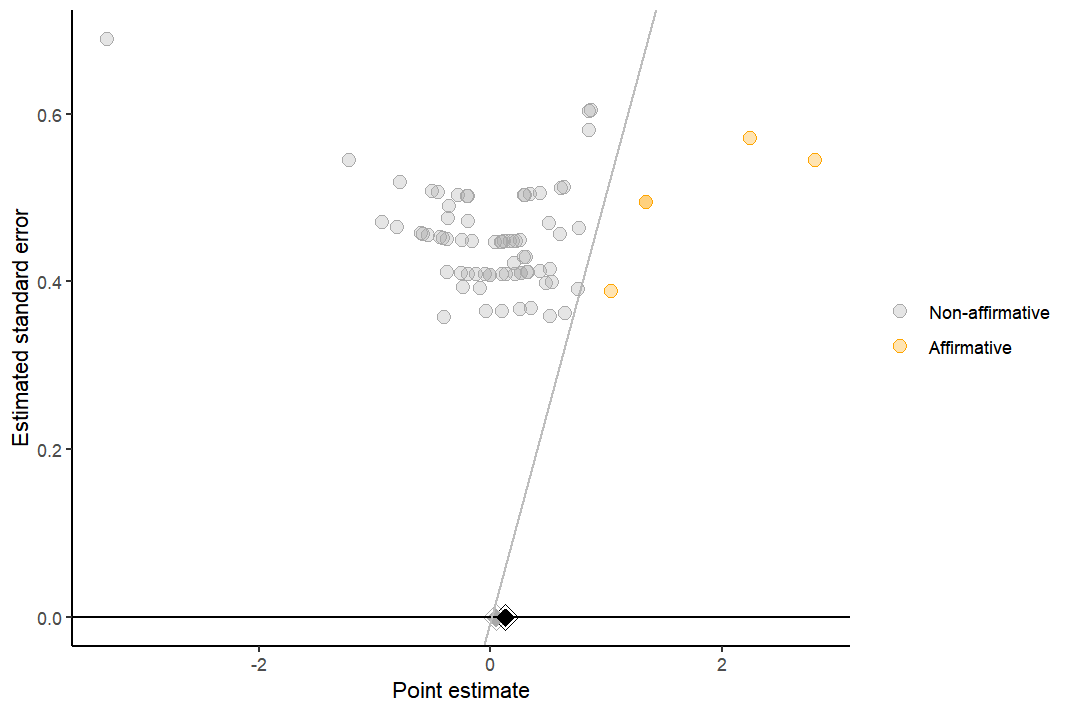


**Fig.S4** The Funnel Plot via PublicationBias Package (Fatigue Recovery)


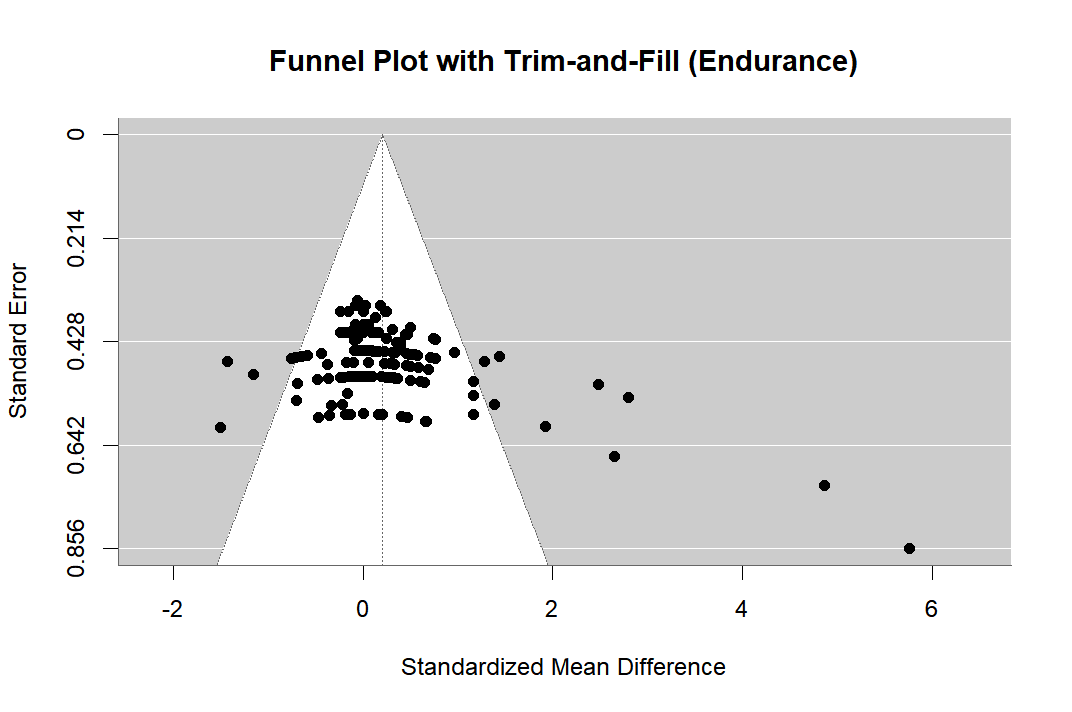


**Fig.S5** The Funnel Plot via Metafor Package (Endurance)


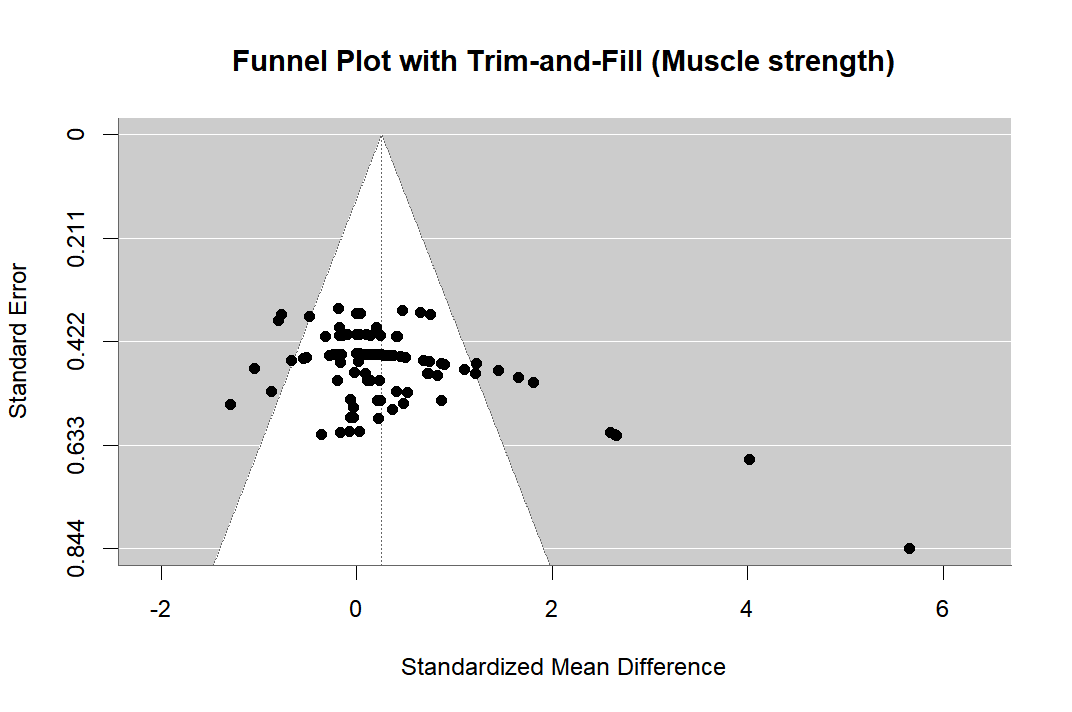


**Fig.S6** The Funnel Plot via Metafor Package (Muscle Strength)


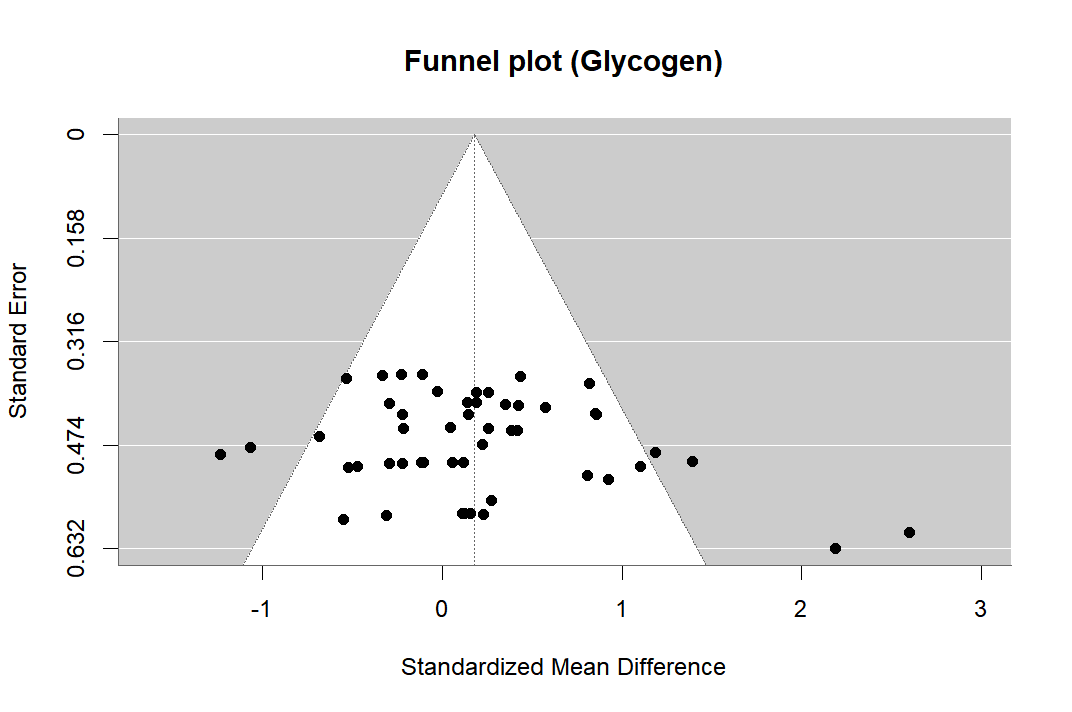


**Fig.S7** The Funnel Plot via Metafor Package (Glycogen Resynthesis)


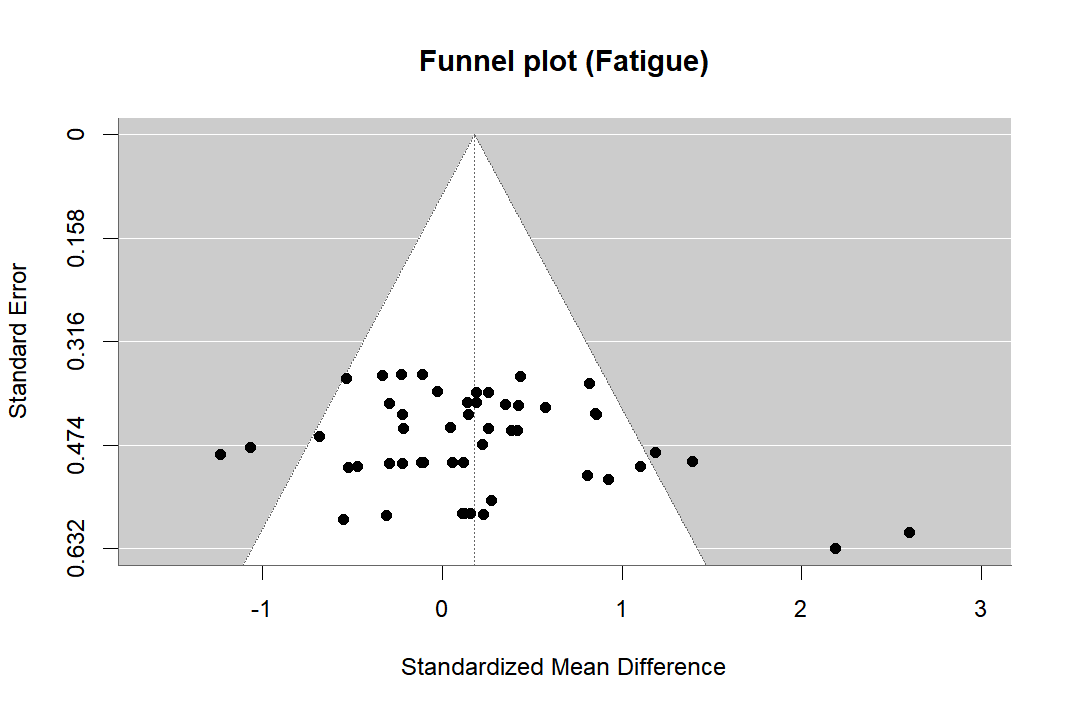


**Fig.S8** The Funnel Plot via Metafor Package (Fatigue Recovery)
