# Supplementary material for: The effectiveness of protein supplements on athletic performance and post-exercise recovery − a Bayesian multilevel meta-analysis of randomized controlled trials
Source: J Int Soc Sports Nutr. 2025 Dec 23;23(1):2605338. doi: 10.1080/15502783.2025.2605338 (PMC12777903; doi:10.1080/15502783.2025.2605338)
Supplement: supplementary material — Supplementary_file_S9. [file RSSN_A_2605338_SM6199.docx]

**Supplementary File S9: GRADE Summary**

**
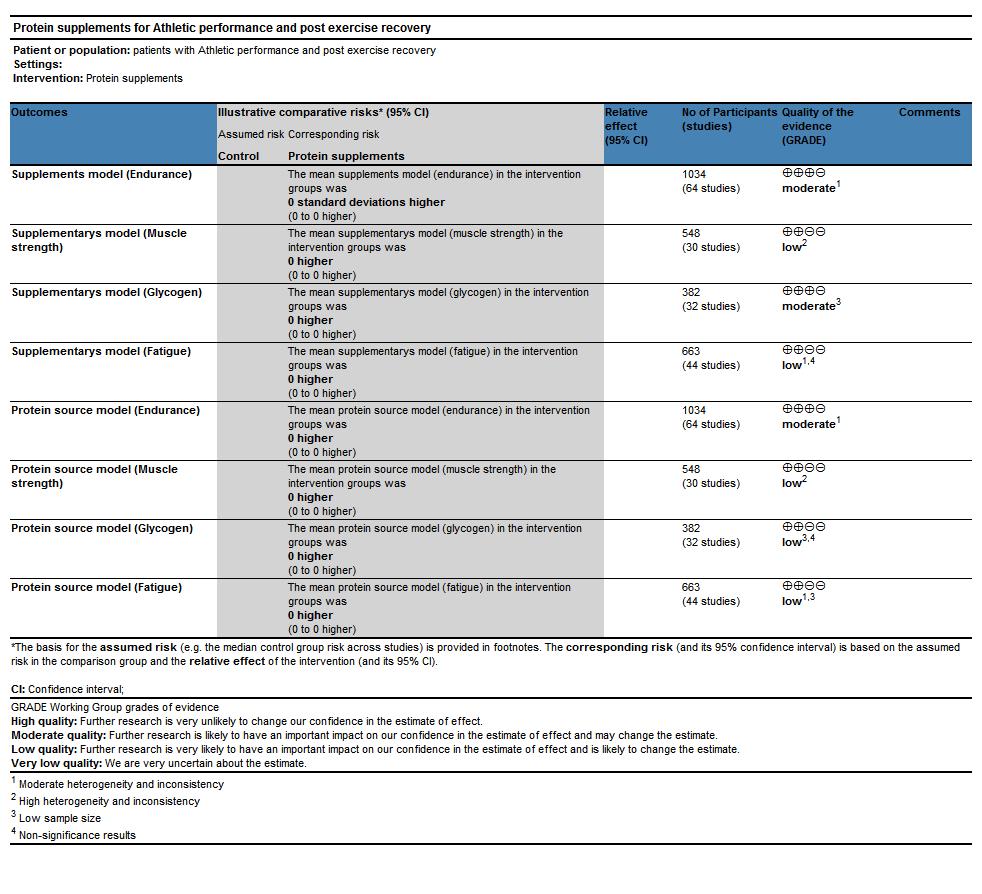
**

**Fig.S1** The GRADE Summary in Supplements and Protein Source Model

**
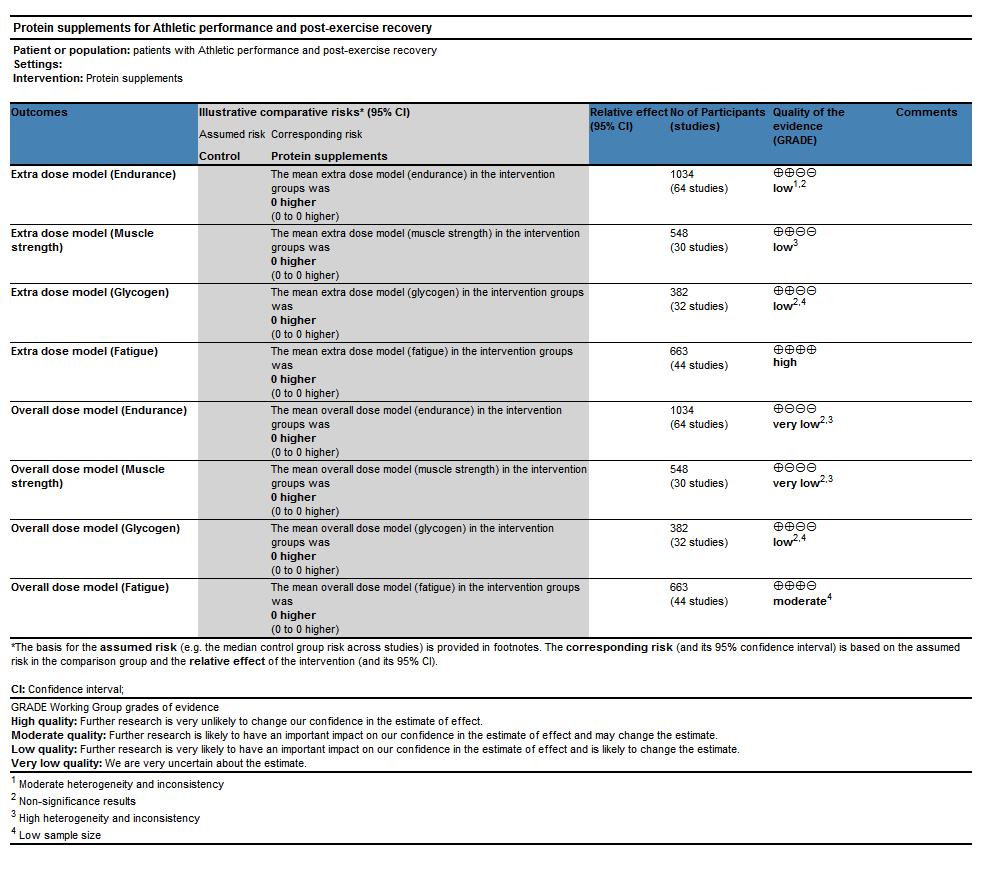
**

**Fig.S2** The GRADE Summary in Extra and Overall Protein Dose Model

**
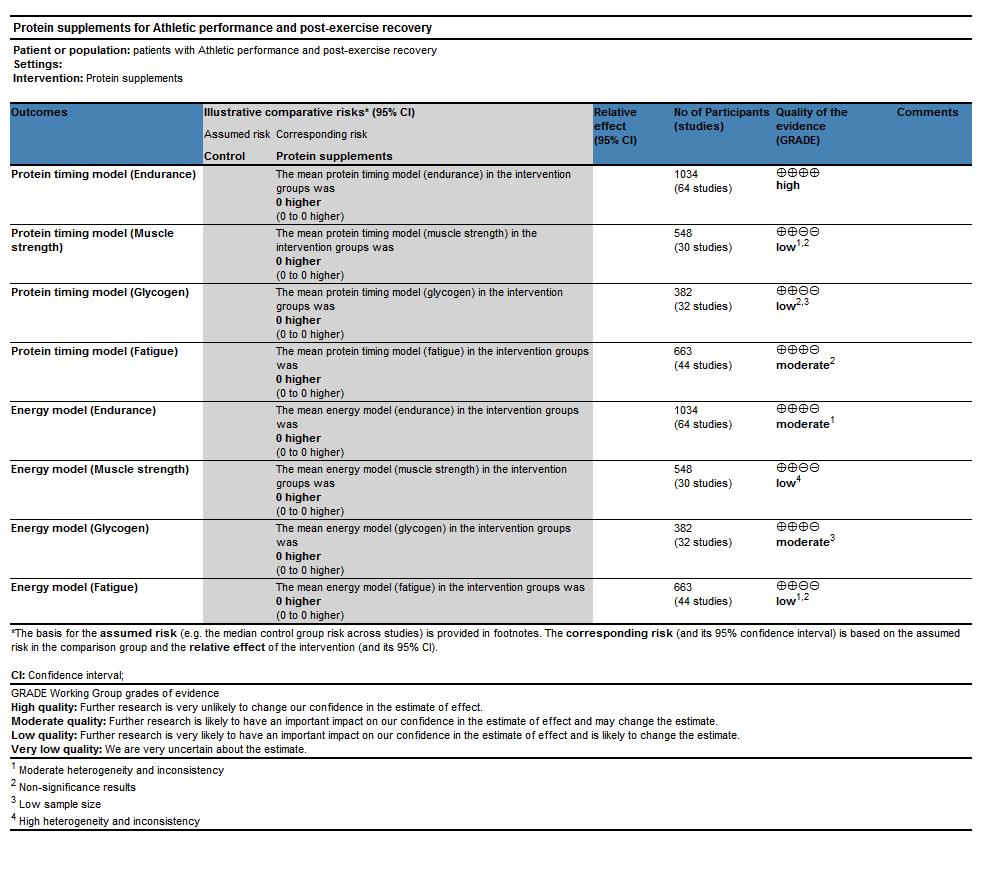
**

**Fig.S3** The GRADE Summary in Protein Timing and Energy Model

**
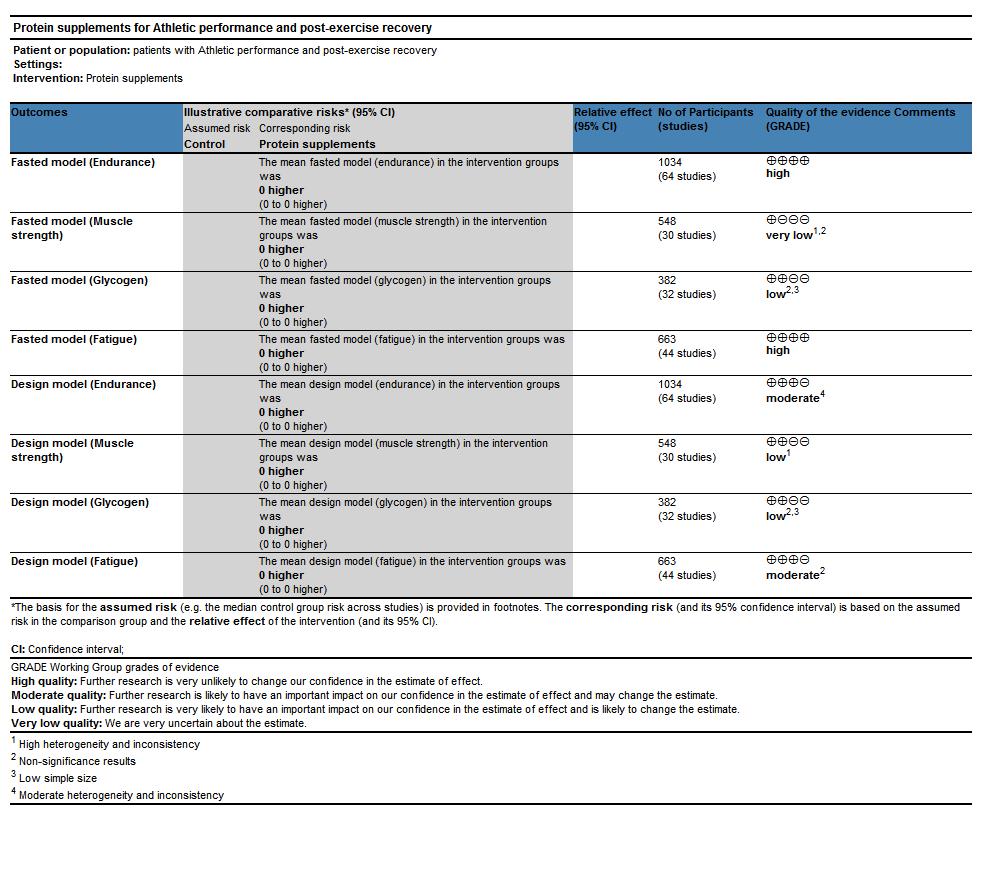
**

**Fig.S4** The GRADE Summary in Fasted and Design Model


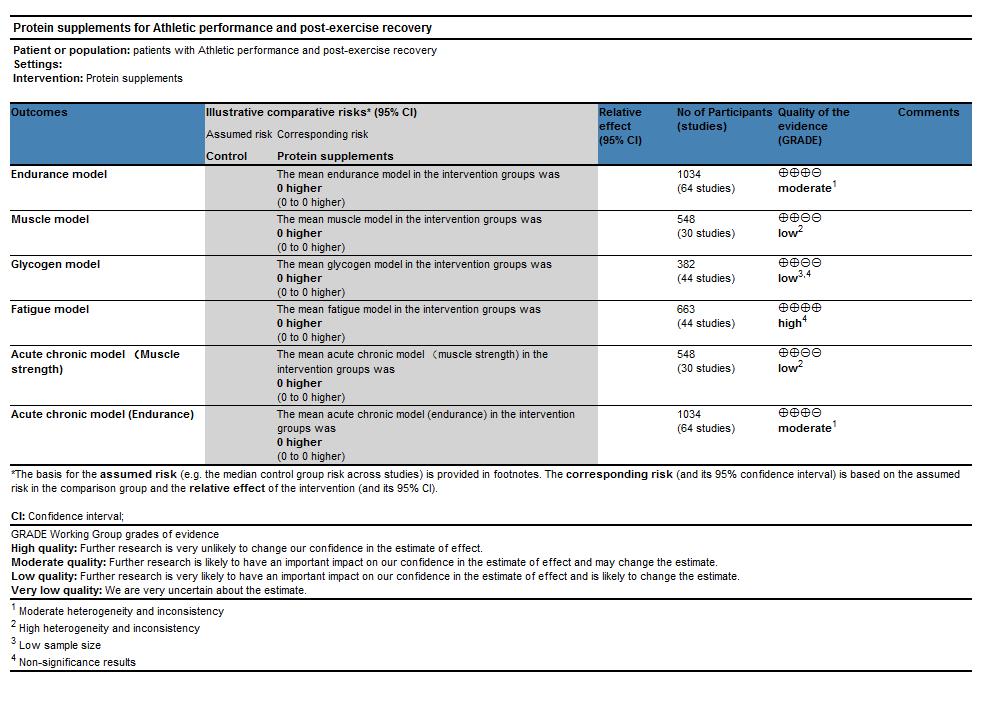


**Fig.S5** The GRADE Summary in Performance Model (Endurance, Muscle Strength, Glycogen and Fatigue) and Acute-Chronic Model


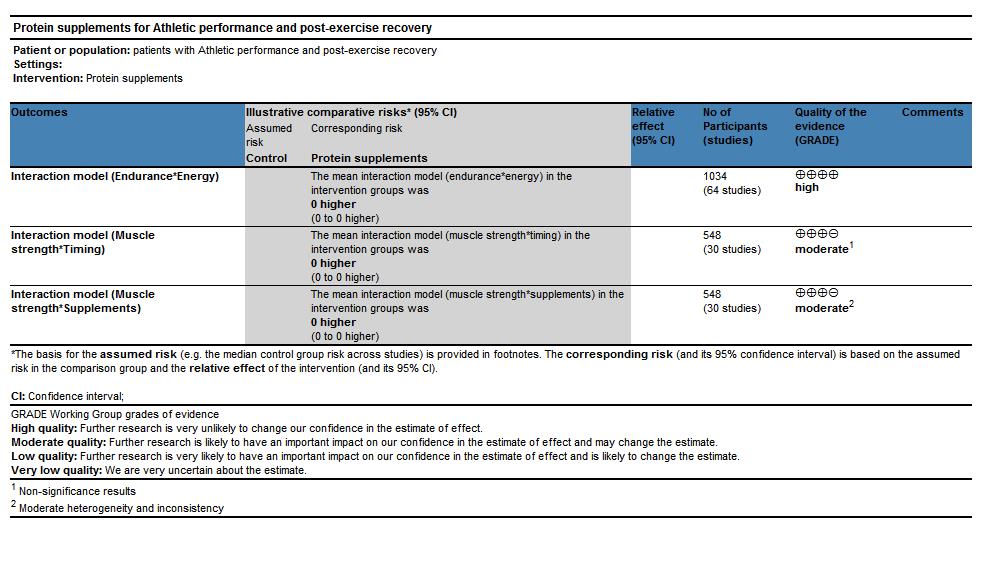


**Fig.S6** The GRADE Summary in Interaction Model
