# Supplementary material for: The effectiveness of protein supplements on athletic performance and post-exercise recovery − a Bayesian multilevel meta-analysis of randomized controlled trials
Source: J Int Soc Sports Nutr. 2025 Dec 23;23(1):2605338. doi: 10.1080/15502783.2025.2605338 (PMC12777903; doi:10.1080/15502783.2025.2605338)
Supplement: supplementary material — Supplementary_file_S8. [file RSSN_A_2605338_SM6198.docx]

**Supplementary File S8: R^2^ Density Plots**

**
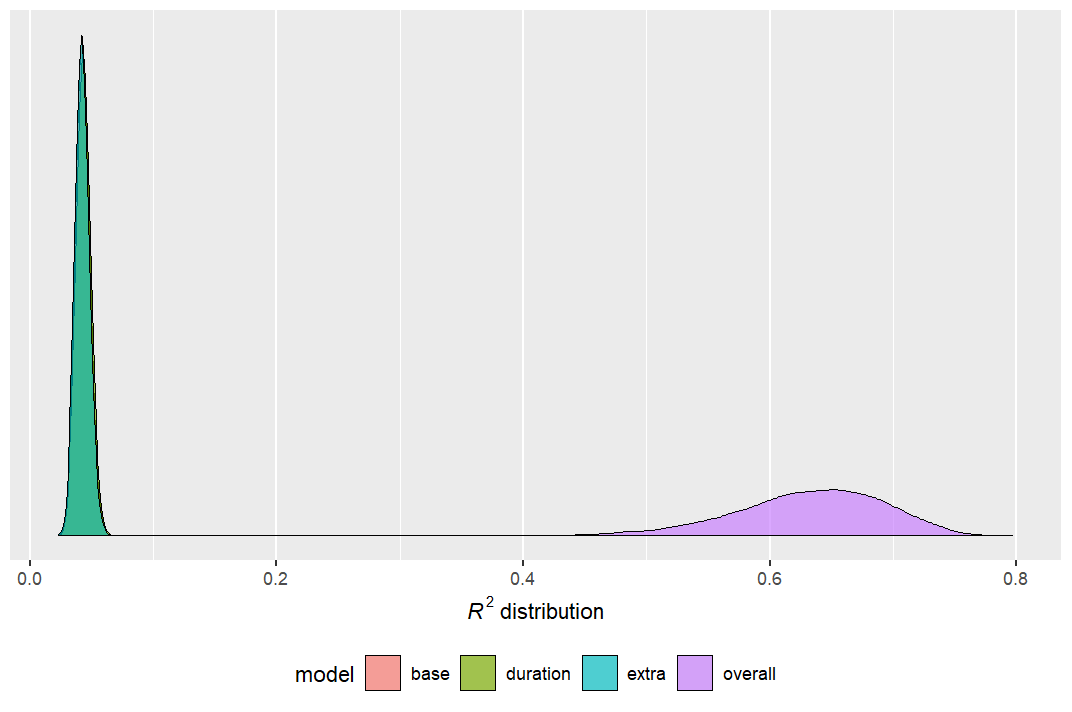
**

**Fig.S1** The R^2^ Density Plot in Non-linear Regression Model (Athletic Performance)


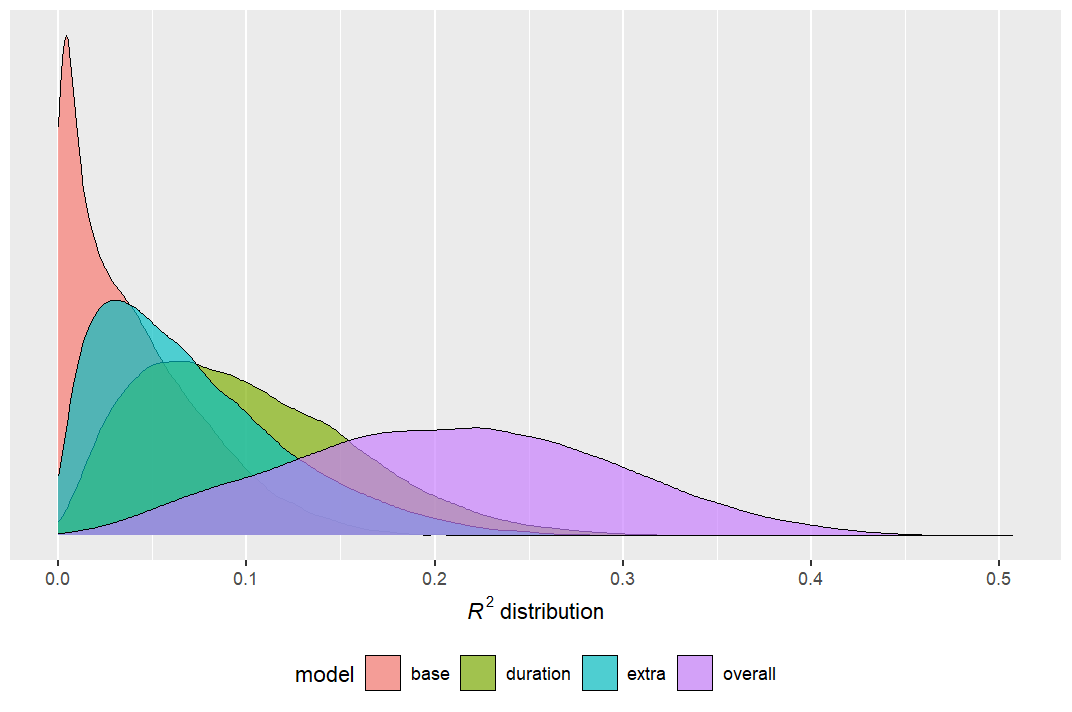


**Fig.S2** The R^2^ Density Plot in Non-linear Regression Model (Post-exercise Recovery)
