# Supplementary material for: The effectiveness of protein supplements on athletic performance and post-exercise recovery − a Bayesian multilevel meta-analysis of randomized controlled trials
Source: J Int Soc Sports Nutr. 2025 Dec 23;23(1):2605338. doi: 10.1080/15502783.2025.2605338 (PMC12777903; doi:10.1080/15502783.2025.2605338)
Supplement: supplementary material — Supplementary_file_S6. [file RSSN_A_2605338_SM6177.docx]

**Supplementary File S6: Multilevel Forest Plots**

**
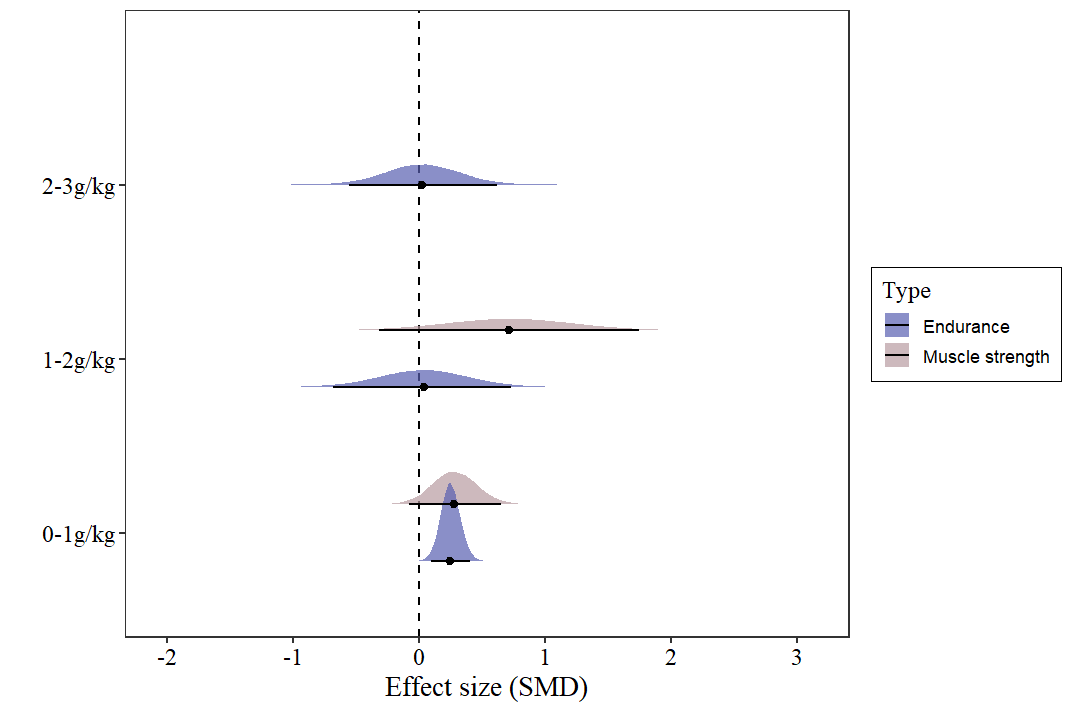
**

**Fig.S1** Forest Plot in Protein Dose Model (Athletic Performance)


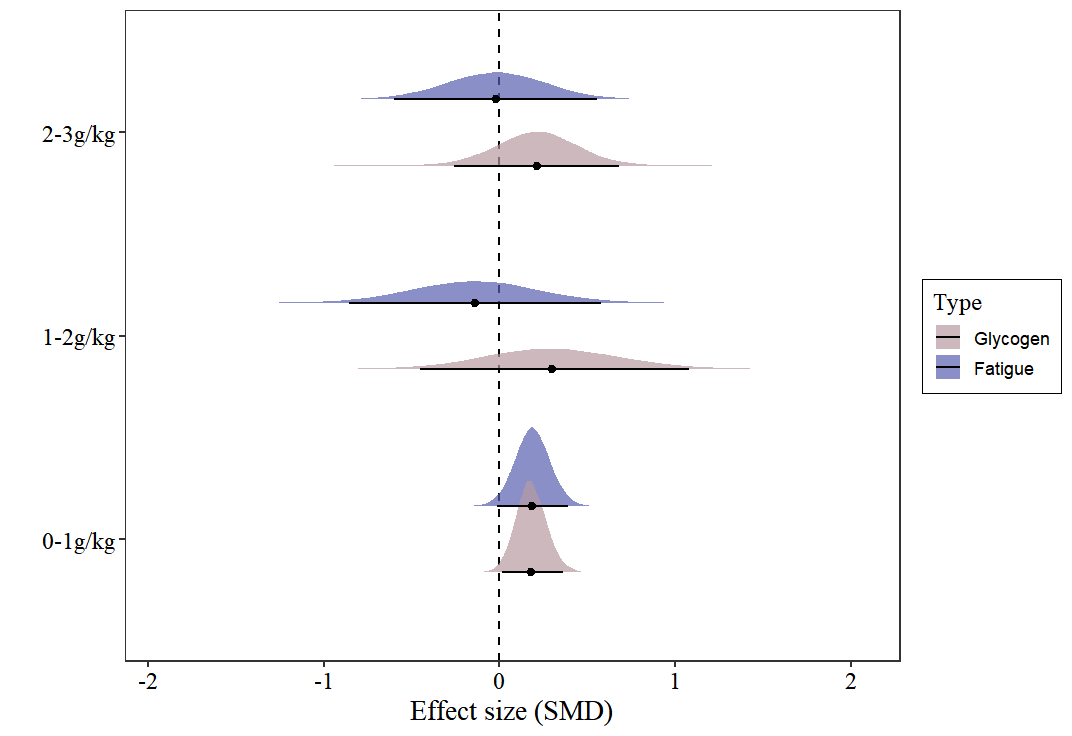


**Fig.S2** Forest Plot in Protein Dose Model (Post-exercise Recovery)


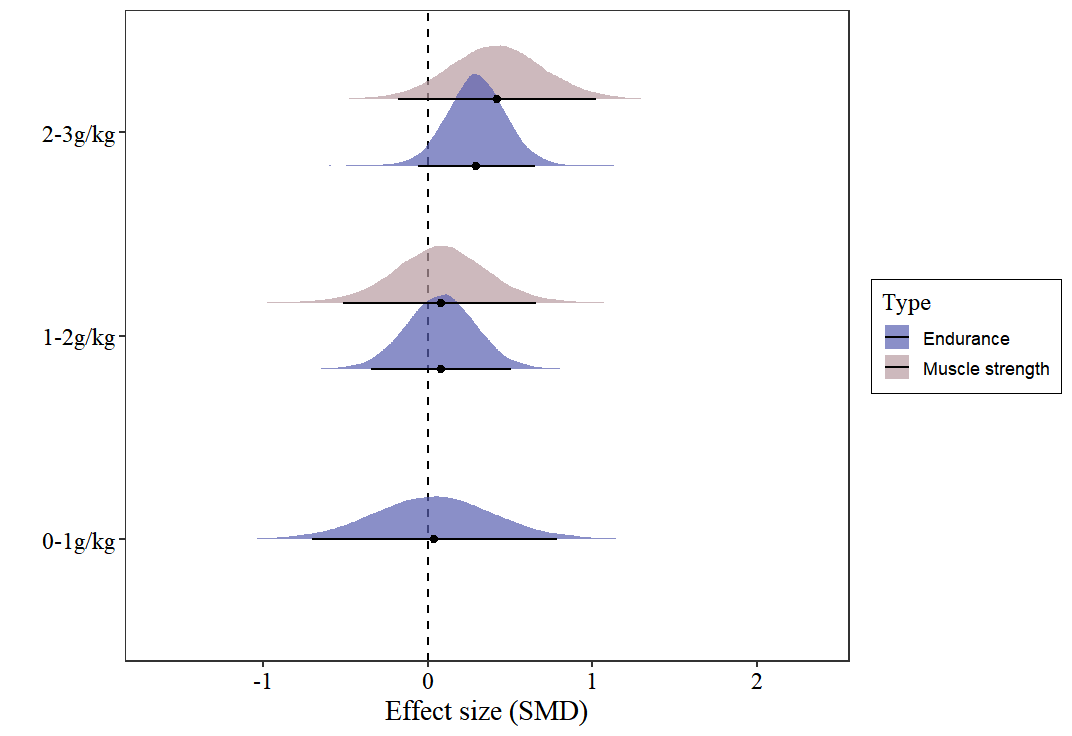


**Fig.S3** Forest Plot in Overall Dose Model (Athletic Performance)


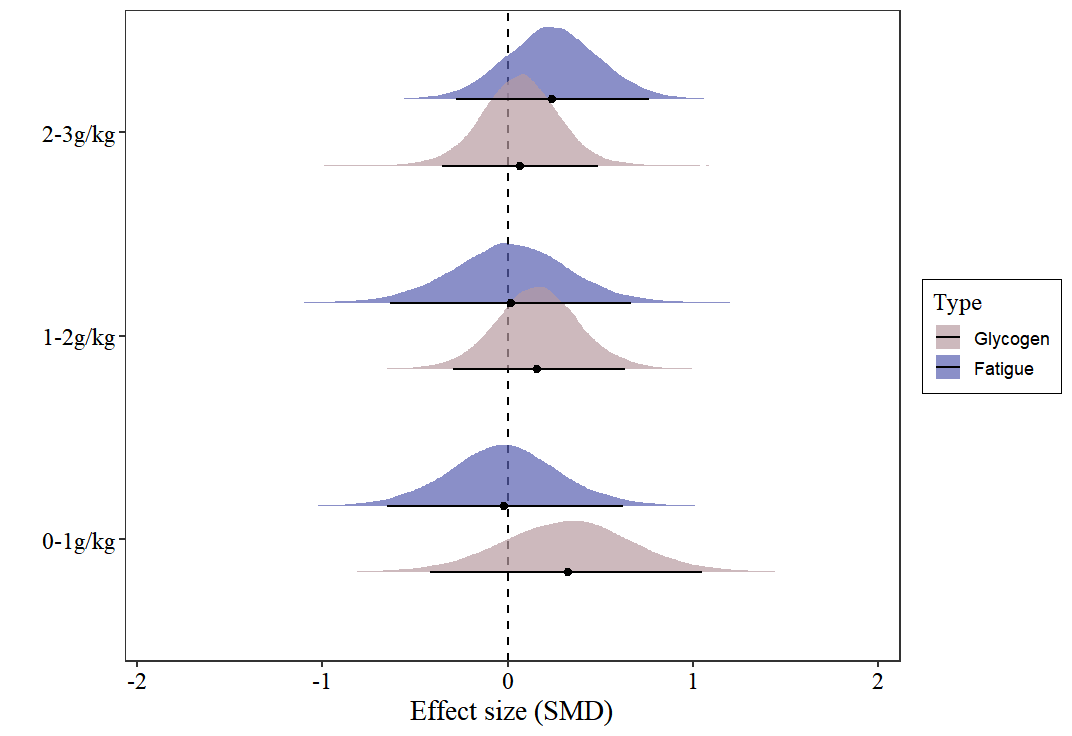


**Fig.S4** Forest Plot in Overall Dose Model (Post-exercise Recovery)

**
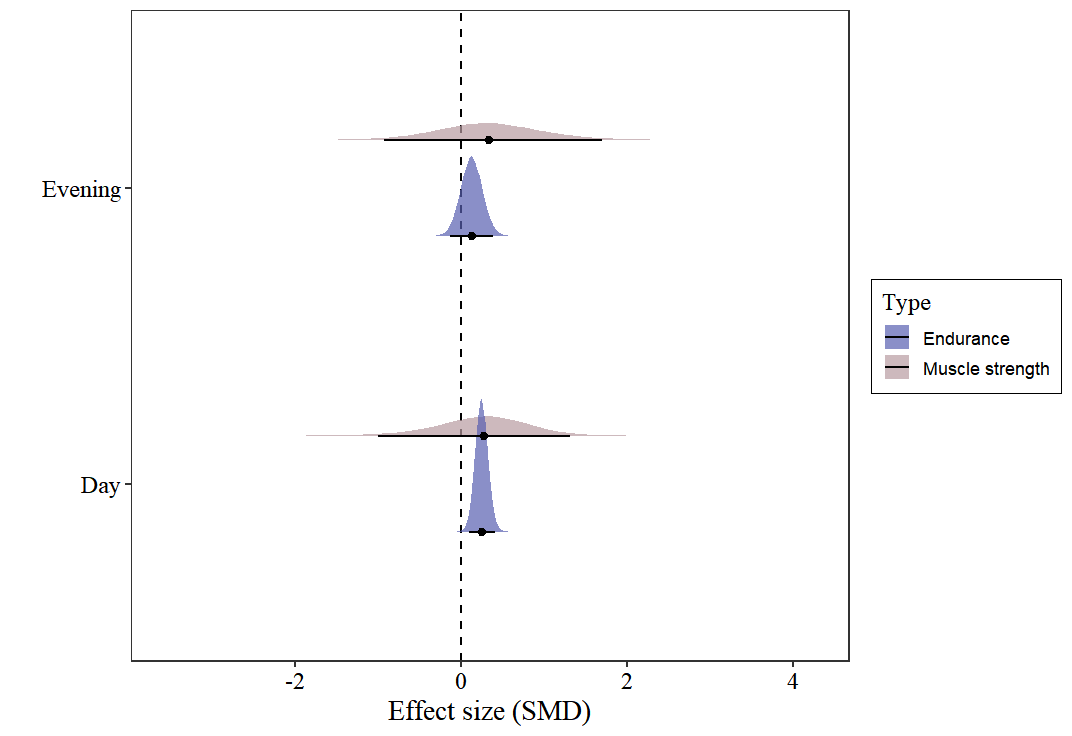
**

**Fig.S5** Forest Plot in Protein Timing Model (Athletic Performance)


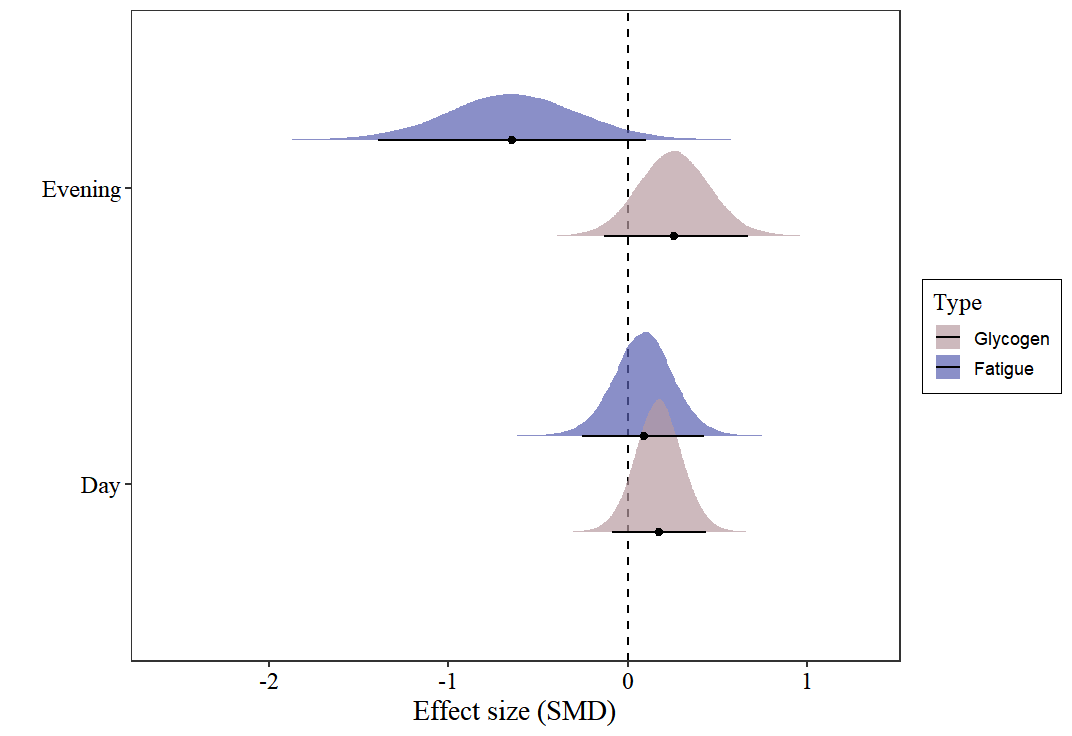


**Fig.S6** Forest Plot in Protein Timing Model (Post-exercise Recovery)


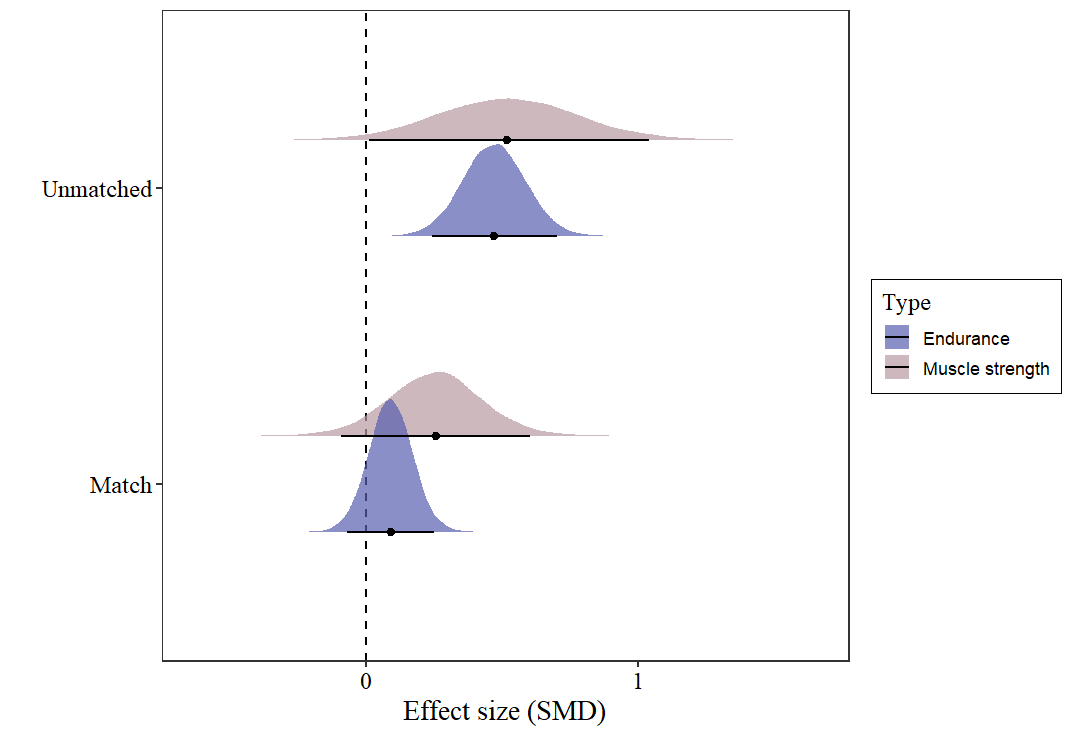


**Fig.S7** Forest Plot in Energy Model (Athletic Performance)


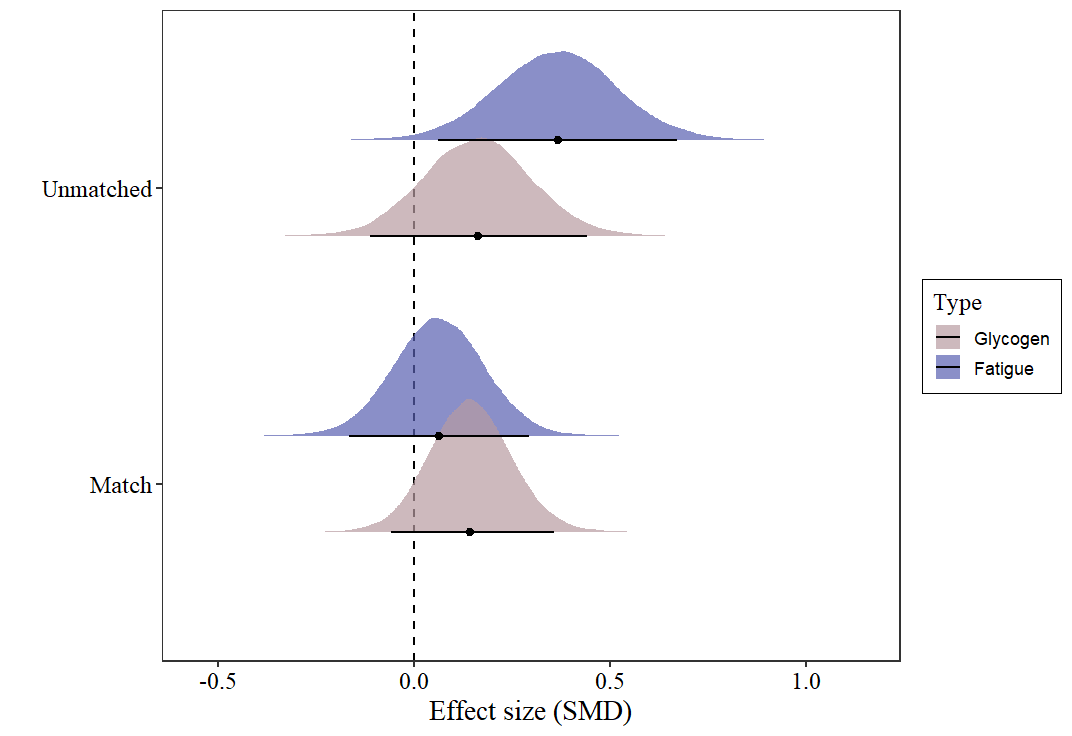


**Fig.S8** Forest Plot in Energy Model (Post-exercise Recovery)


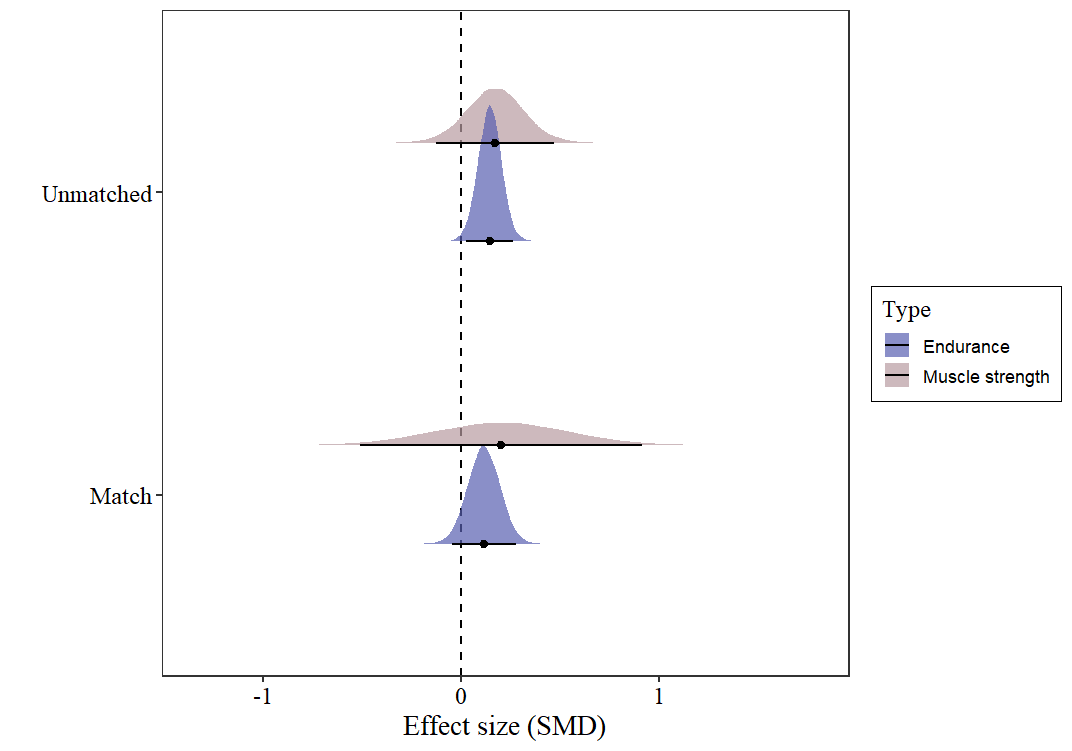


**Fig.S9** Forest Plot in Fasted Model (Athletic Performance)


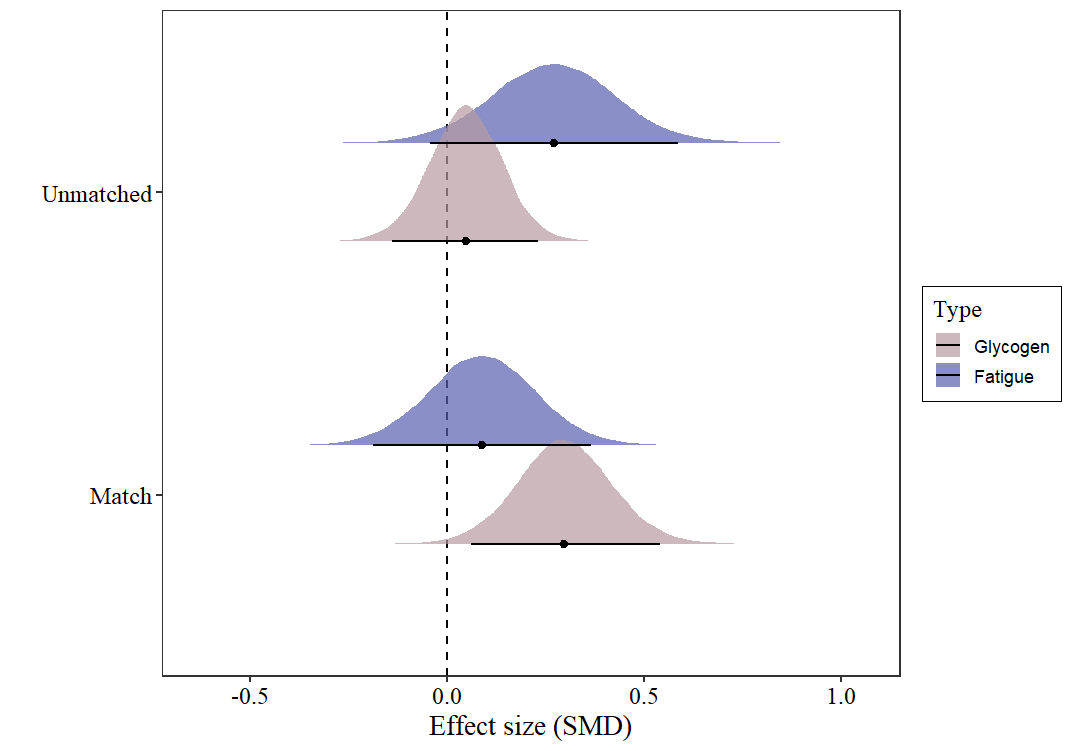


**Fig.S10** Forest Plot in Fasted Model (Post-exercise Recovery)


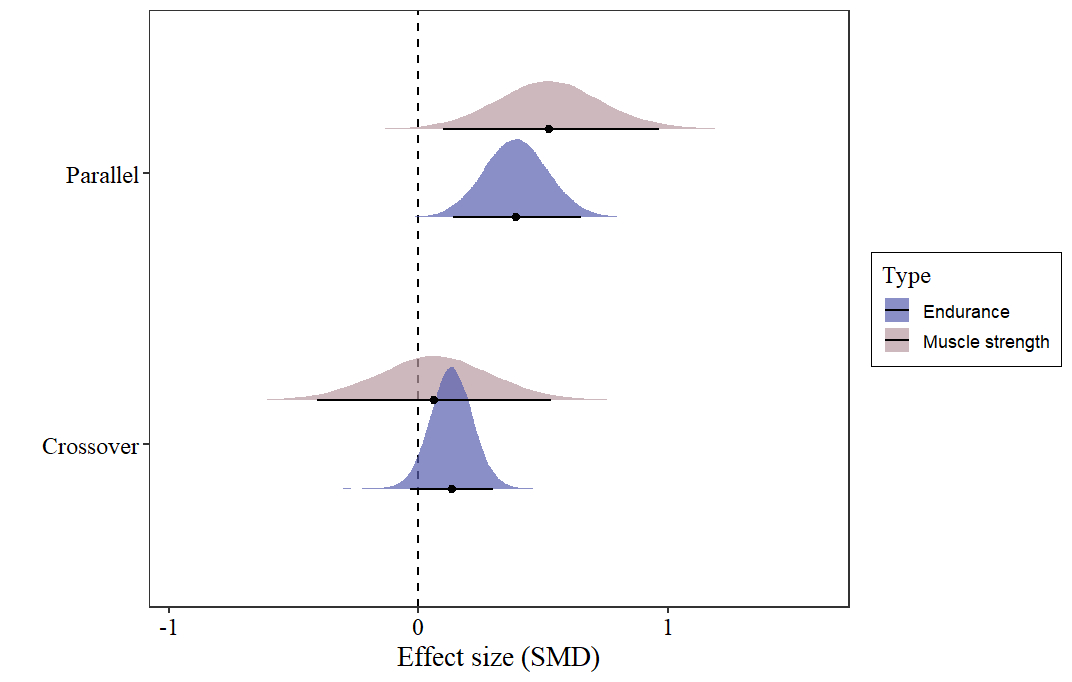


**Fig.S11** Forest Plot in Design Model (Athletic Performance)


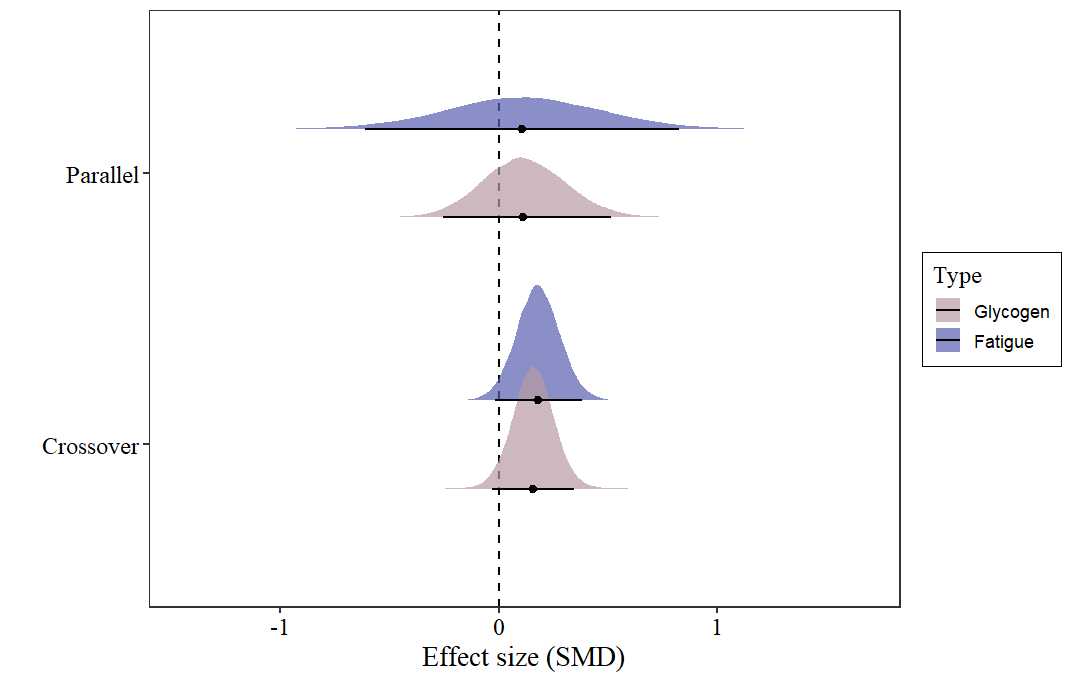


**Fig.S12** Forest Plot in Design Model (Post-exercise Recovery)


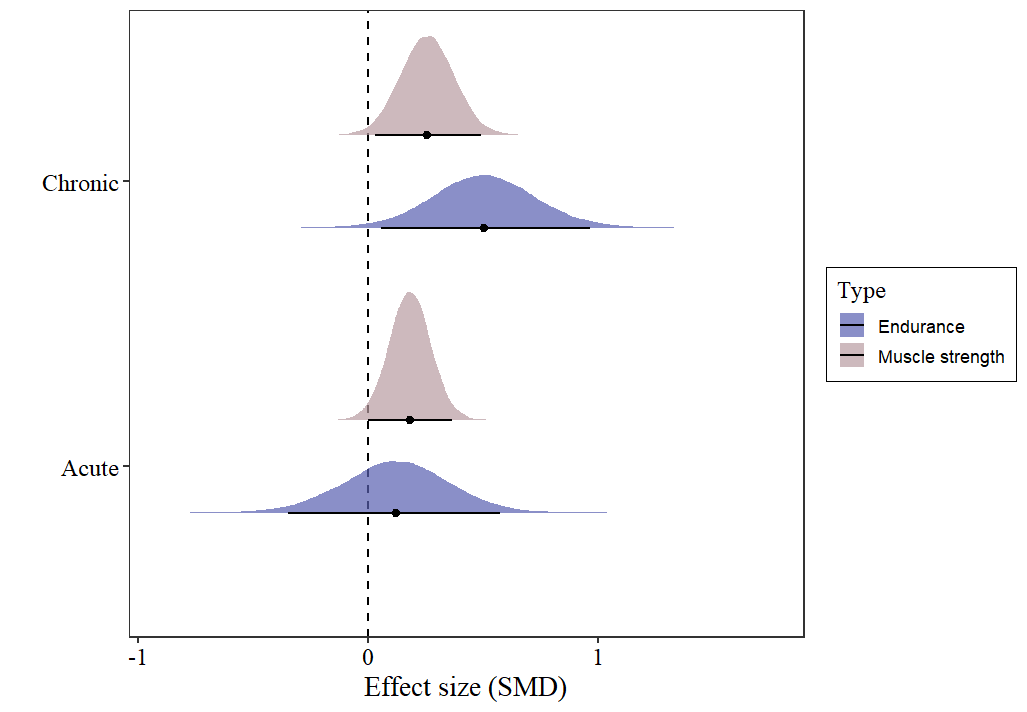


**Fig.S13** Forest Plot in Acute-Chronic Model


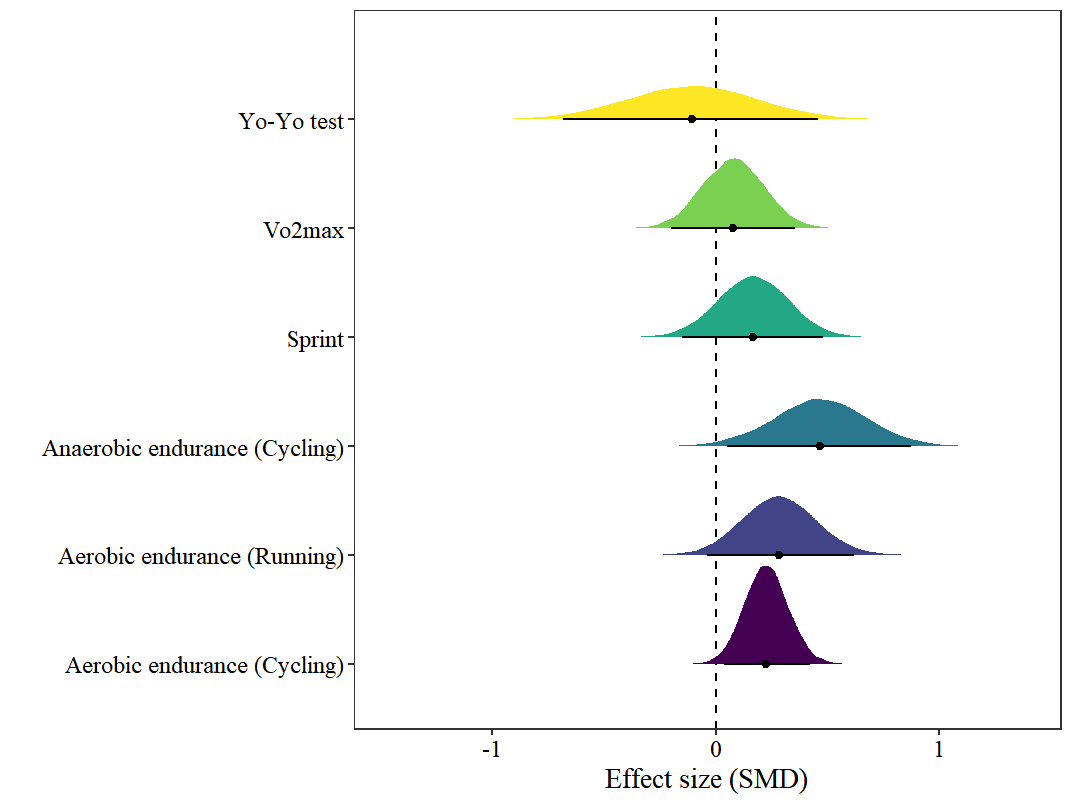


**Fig.S14** Forest Plot in Endurance Model


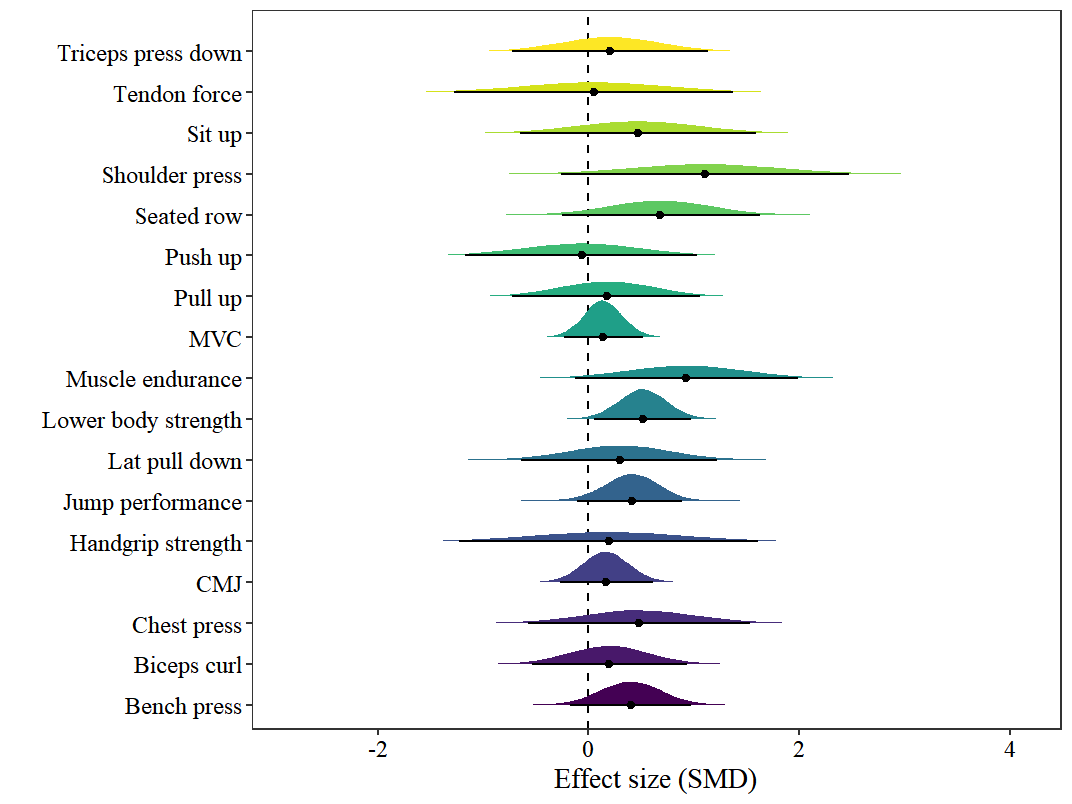


**Fig.S15** Forest Plot in Muscle Model


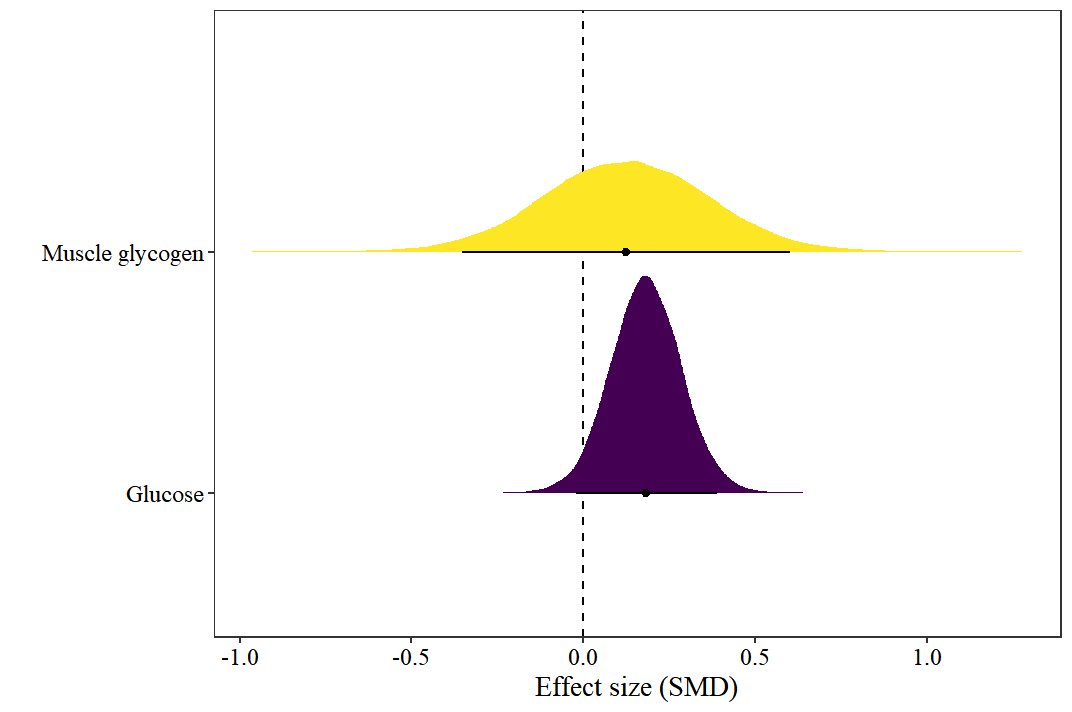


**Fig.S16** Forest Plot in Glycogen Model


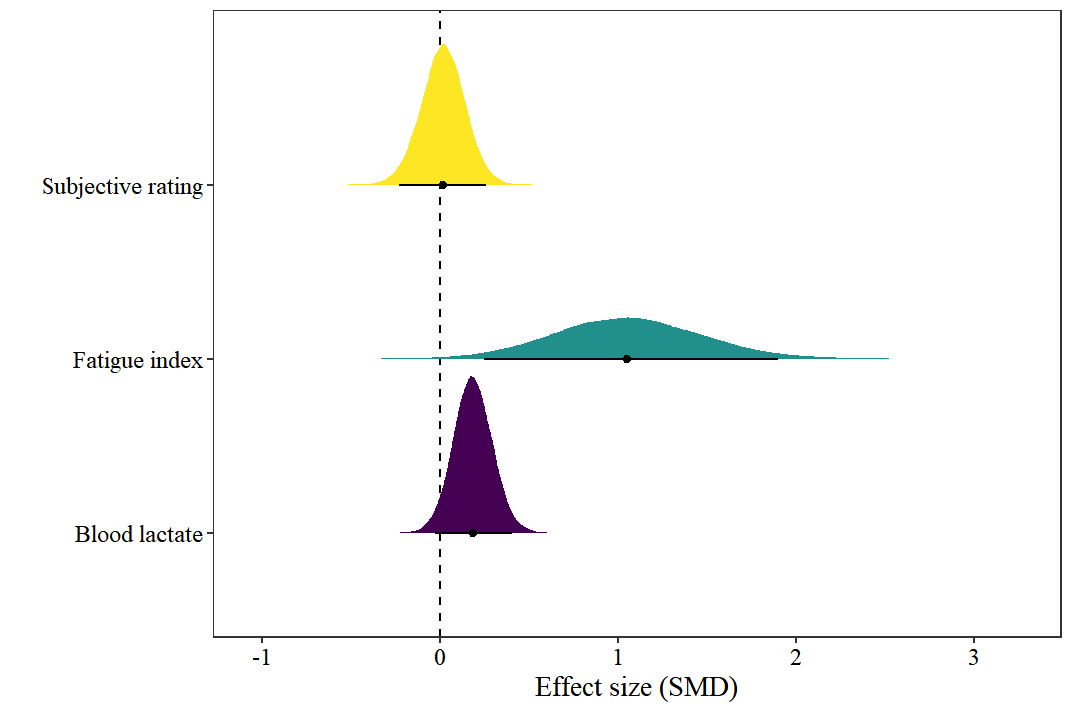


**Fig.S17** Forest Plot in Fatigue Model
