# Supplementary material for: The effectiveness of protein supplements on athletic performance and post-exercise recovery − a Bayesian multilevel meta-analysis of randomized controlled trials
Source: J Int Soc Sports Nutr. 2025 Dec 23;23(1):2605338. doi: 10.1080/15502783.2025.2605338 (PMC12777903; doi:10.1080/15502783.2025.2605338)
Supplement: supplementary material — Supplementary_file_S5. [file RSSN_A_2605338_SM6160.docx]

**Supplementary File S5:** Forest Plots and Cumulative Probability, HDI Distribution, and Posterior Density Distribution (SMD, τ_within_ and τ_between_) in the Null Model


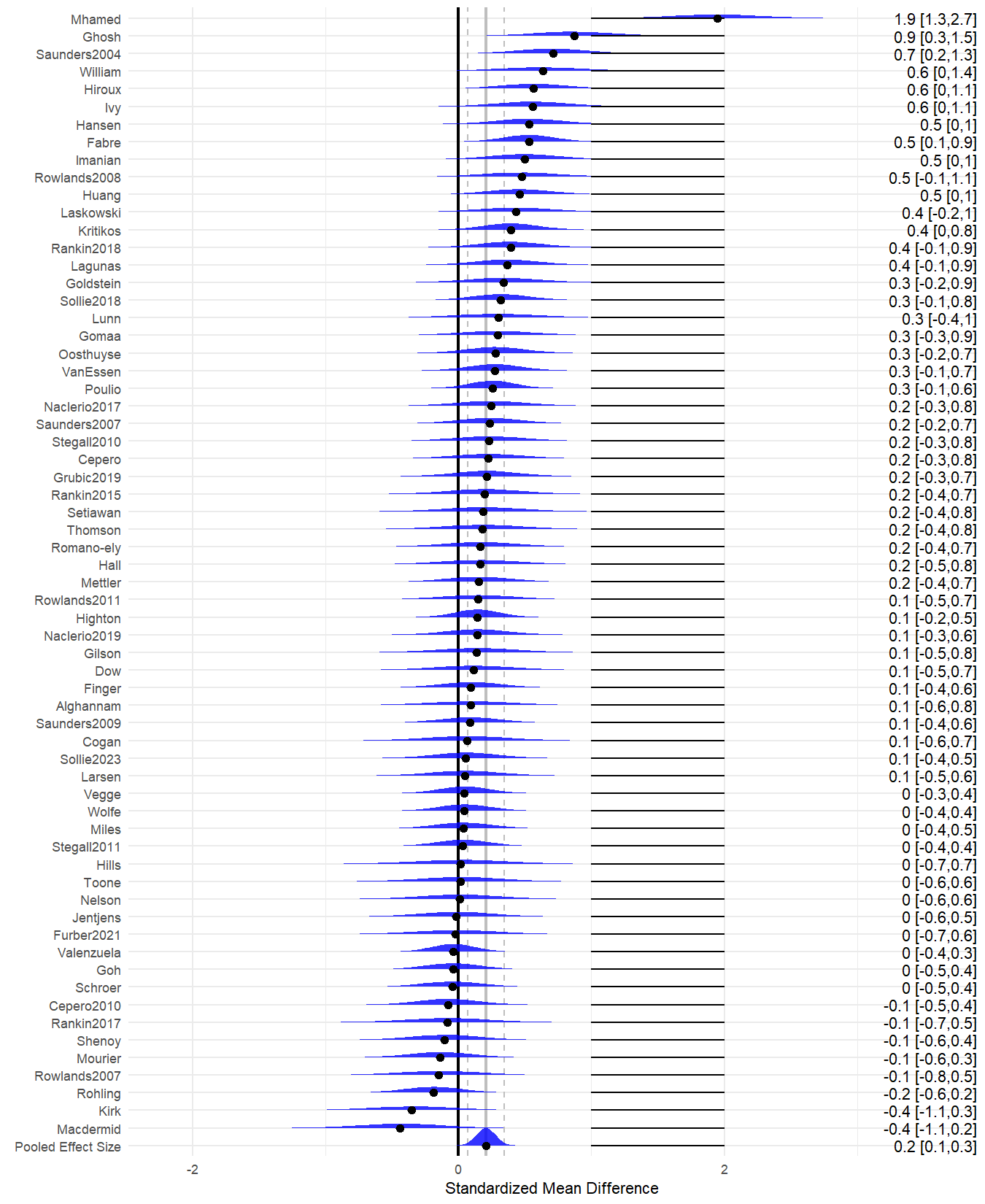


**Fig.S1** Forest Plot of Endurance Performance in the Null Model


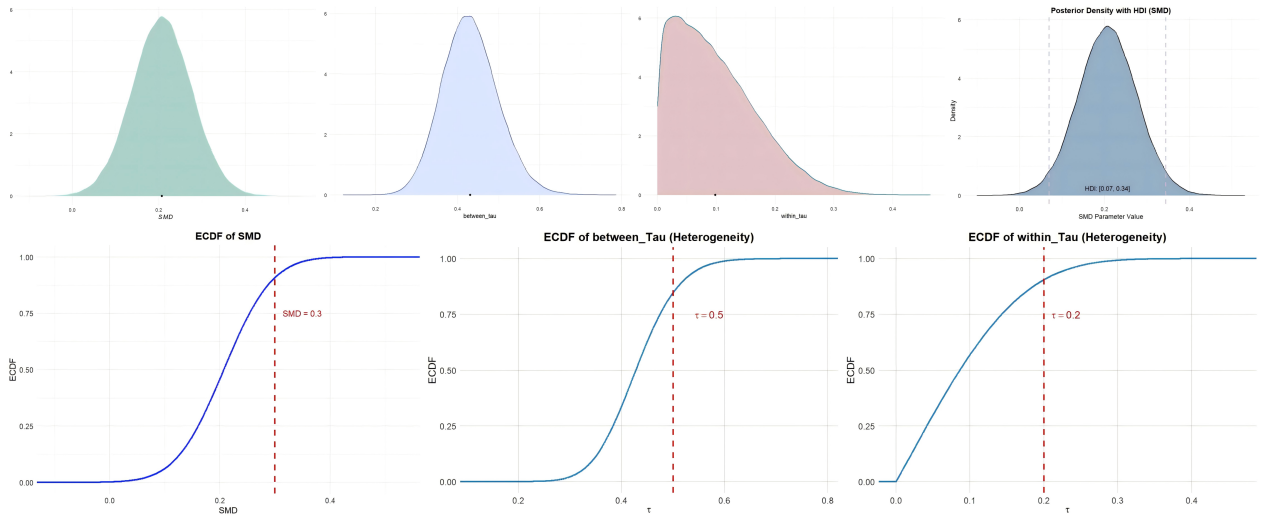


**Fig.S2** The Cumulative Probability, HDI Distribution, and Posterior Density Distribution (SMD, τ_within_, and τ_between_) in Endurance Performance


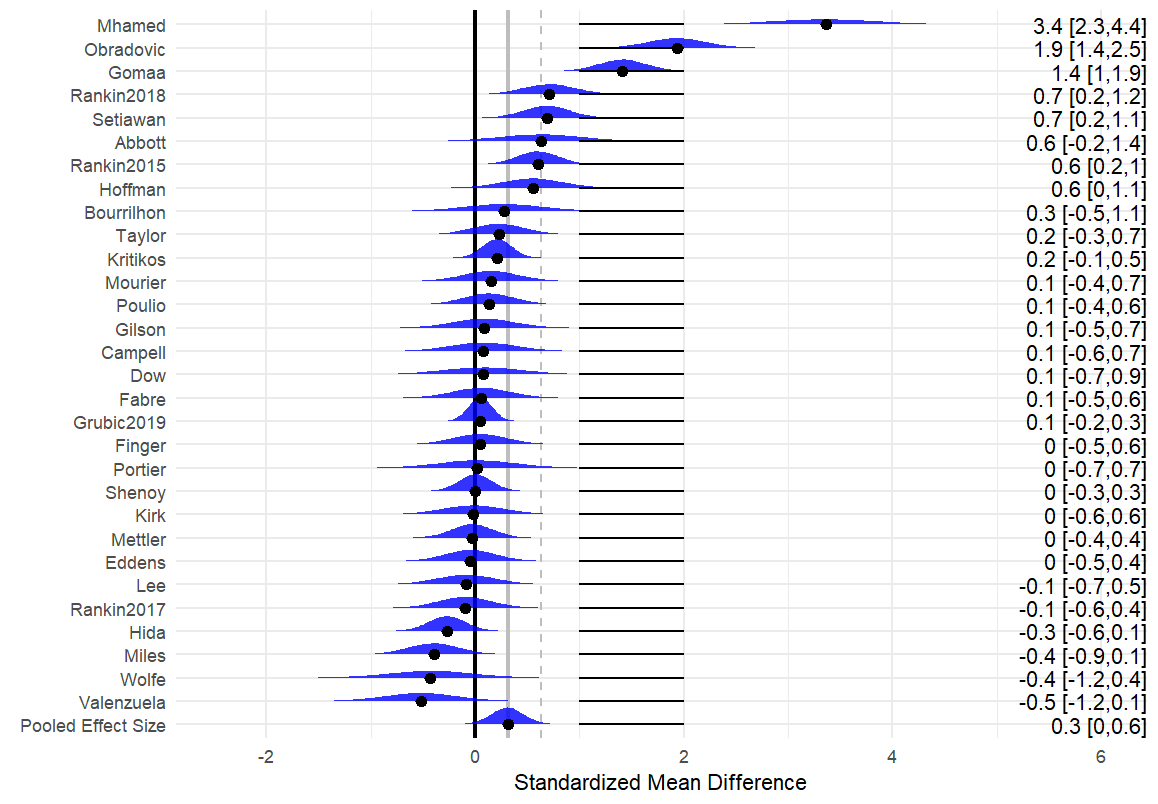


**Fig.S3** Forest Plot of Muscle Strength in the Null Model


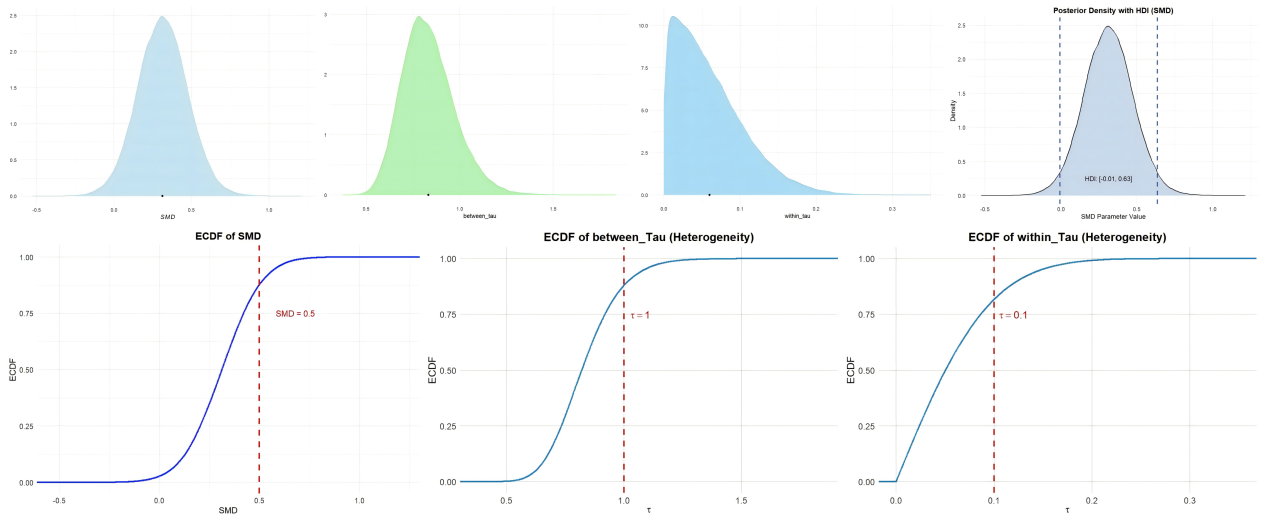


**Fig.S4** The Cumulative Probability, HDI Distribution, and Posterior Density Distribution (SMD, τ_within_, and τ_between_) in Muscle Strength


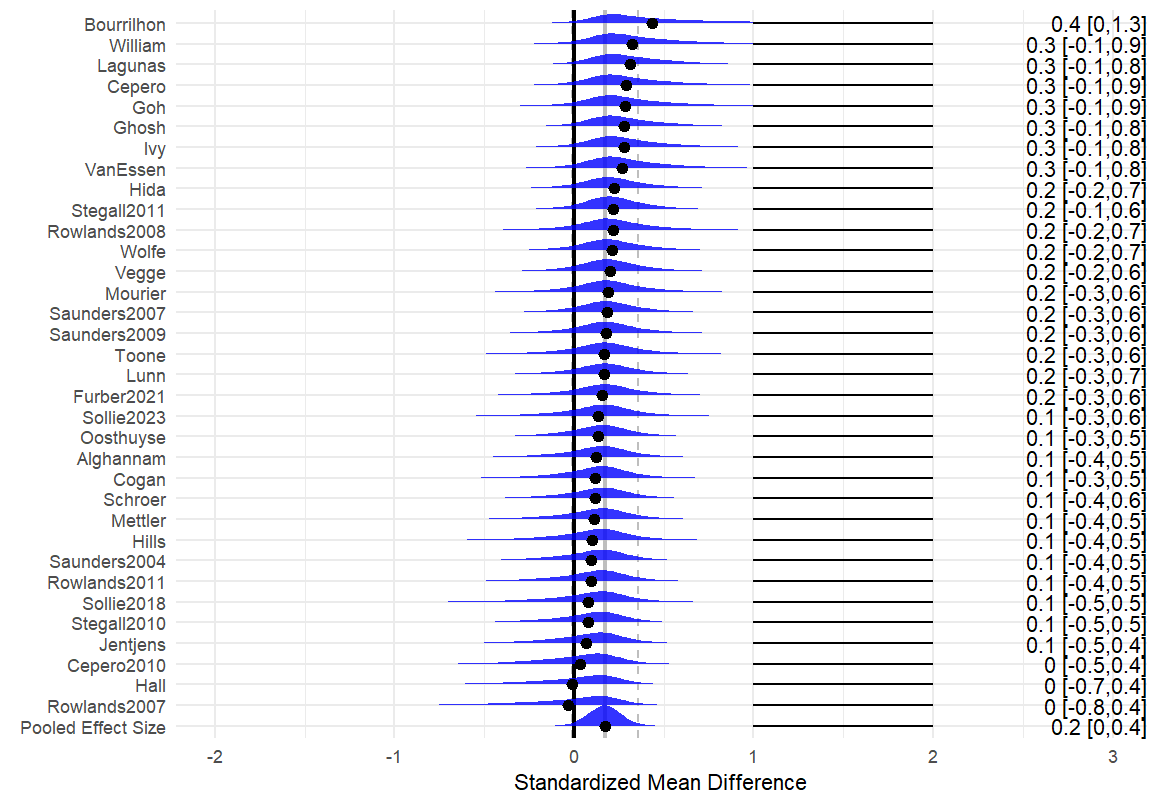


**Fig.S5** Forest Plot of Glycogen Resynthesis in the Null Model


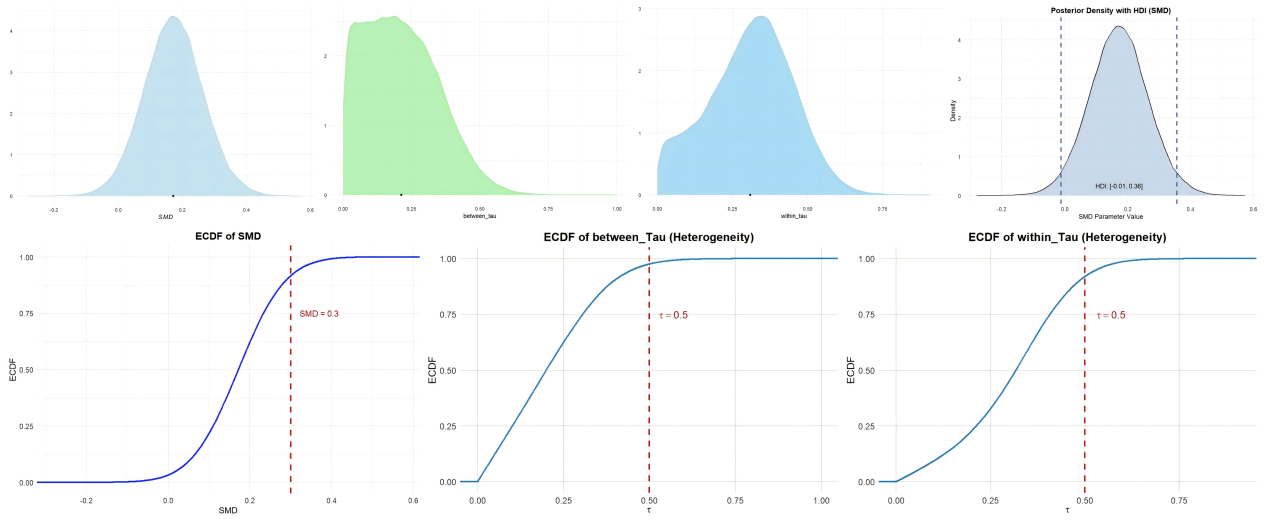


**Fig.S6** The Cumulative Probability, HDI Distribution, and Posterior Density Distribution (SMD, τ_within_, and τ_between_) in Glycogen Resynthesis


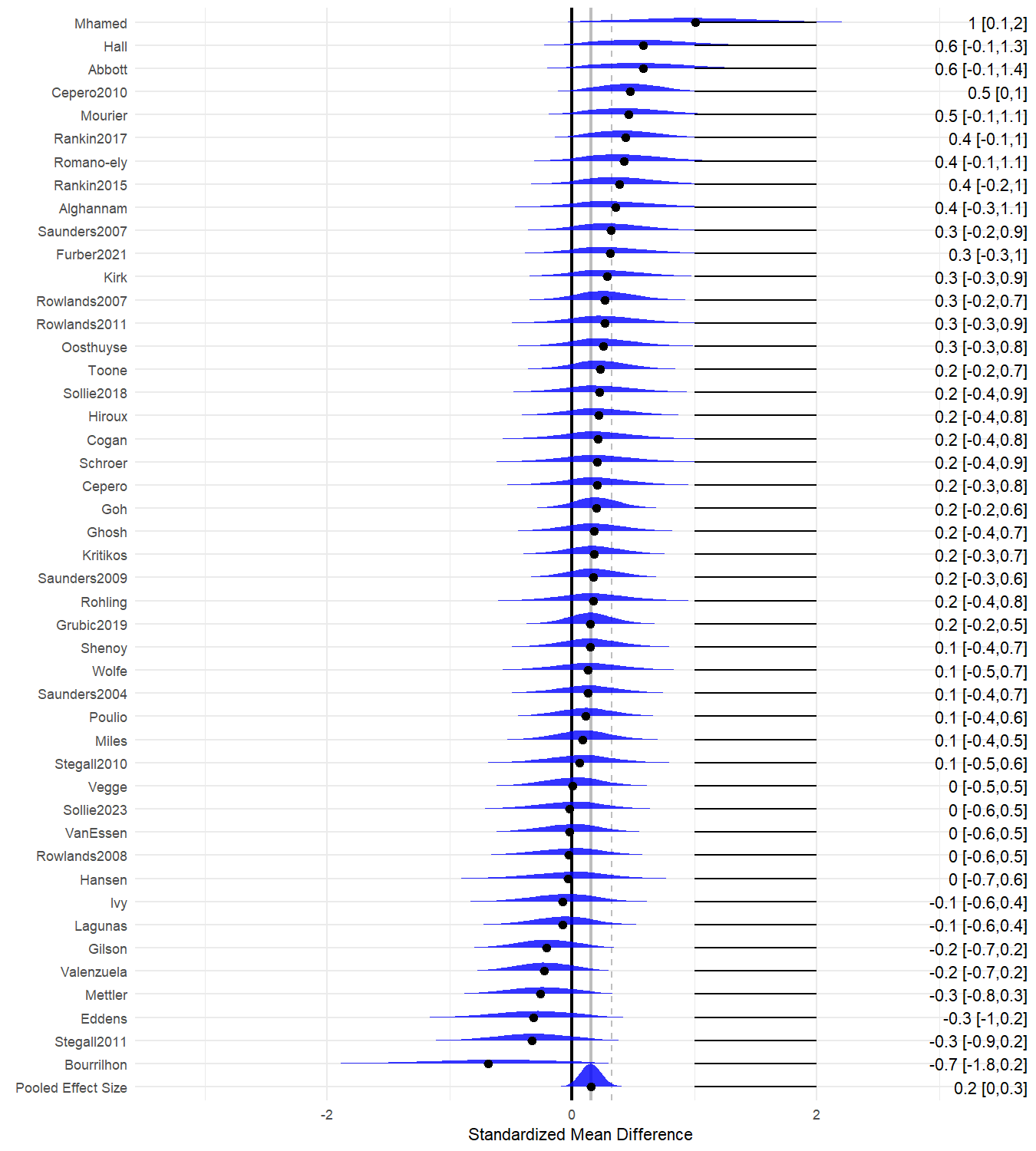


**Fig.S7** Forest Plot of Fatigue Recovery in the Null Model


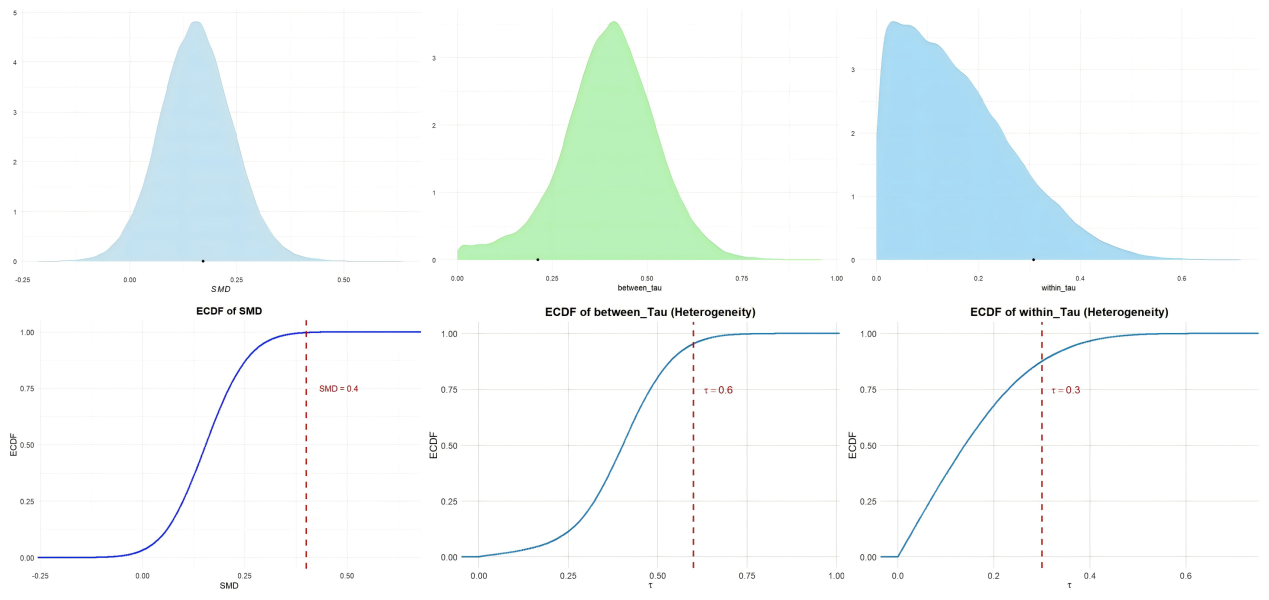


**Fig.S8** The Cumulative Probability and Posterior Density Distribution (SMD, τ_within,_ and τ_between_) in Fatigue Recovery
