# Supplementary material for: The effectiveness of protein supplements on athletic performance and post-exercise recovery − a Bayesian multilevel meta-analysis of randomized controlled trials
Source: J Int Soc Sports Nutr. 2025 Dec 23;23(1):2605338. doi: 10.1080/15502783.2025.2605338 (PMC12777903; doi:10.1080/15502783.2025.2605338)
Supplement: supplementary material — Supplementary_file_S3. [file RSSN_A_2605338_SM6153.docx]

**Supplementary File S3: Risk of bias (RoB) Assessment**

**
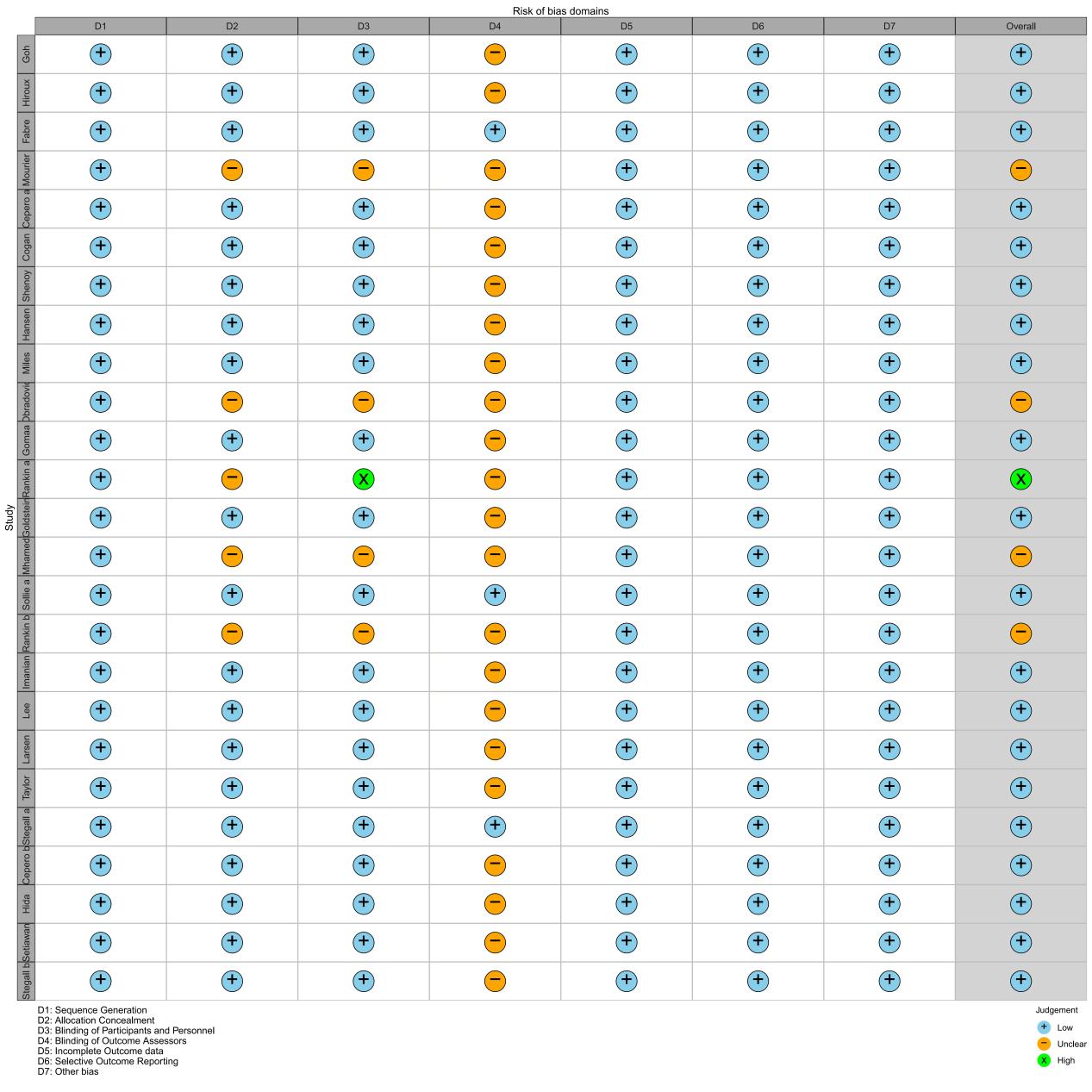
**

**Fig.S1** Risk of Bias Assessment (a)

**
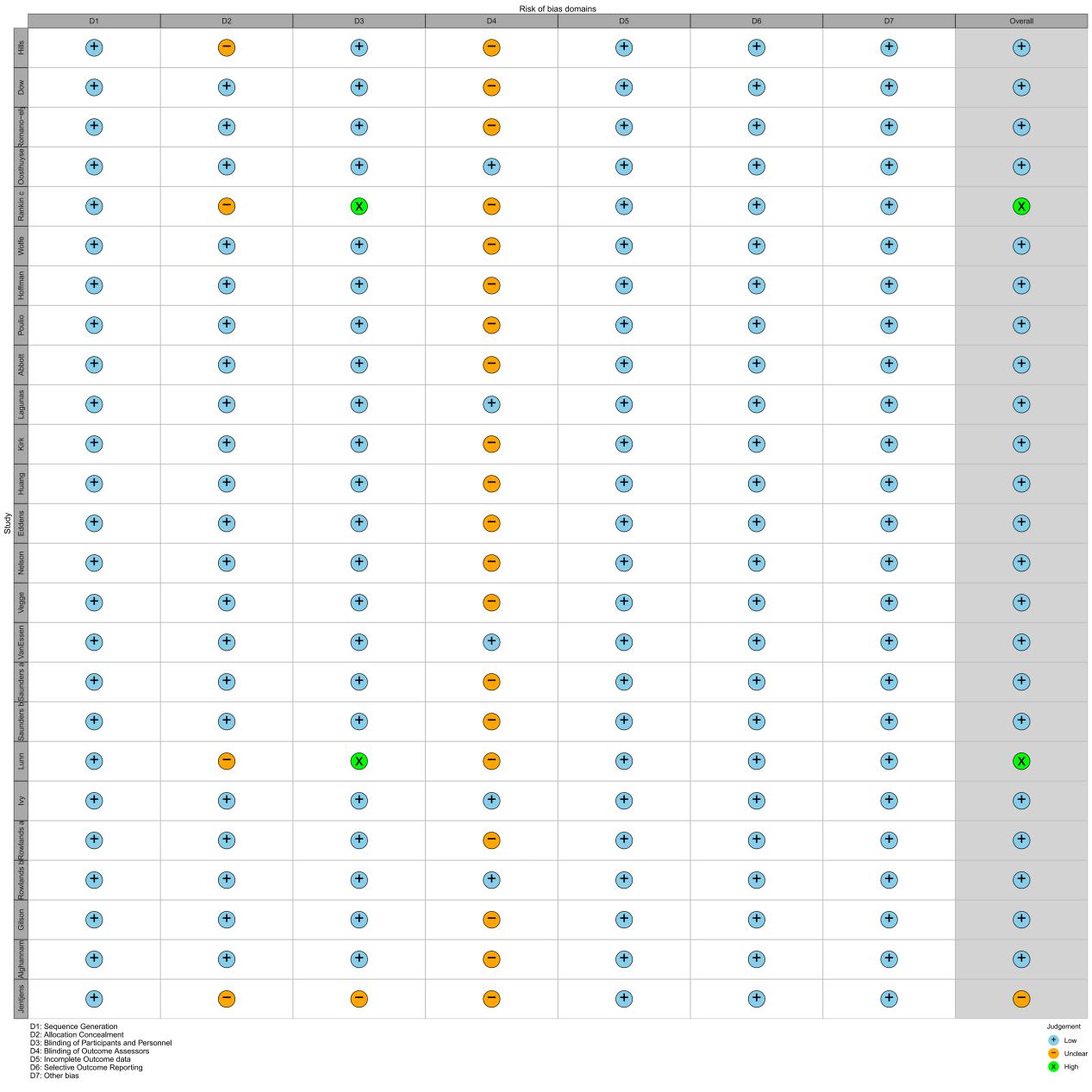
**

**Fig.S2** Risk of Bias Assessment (b)


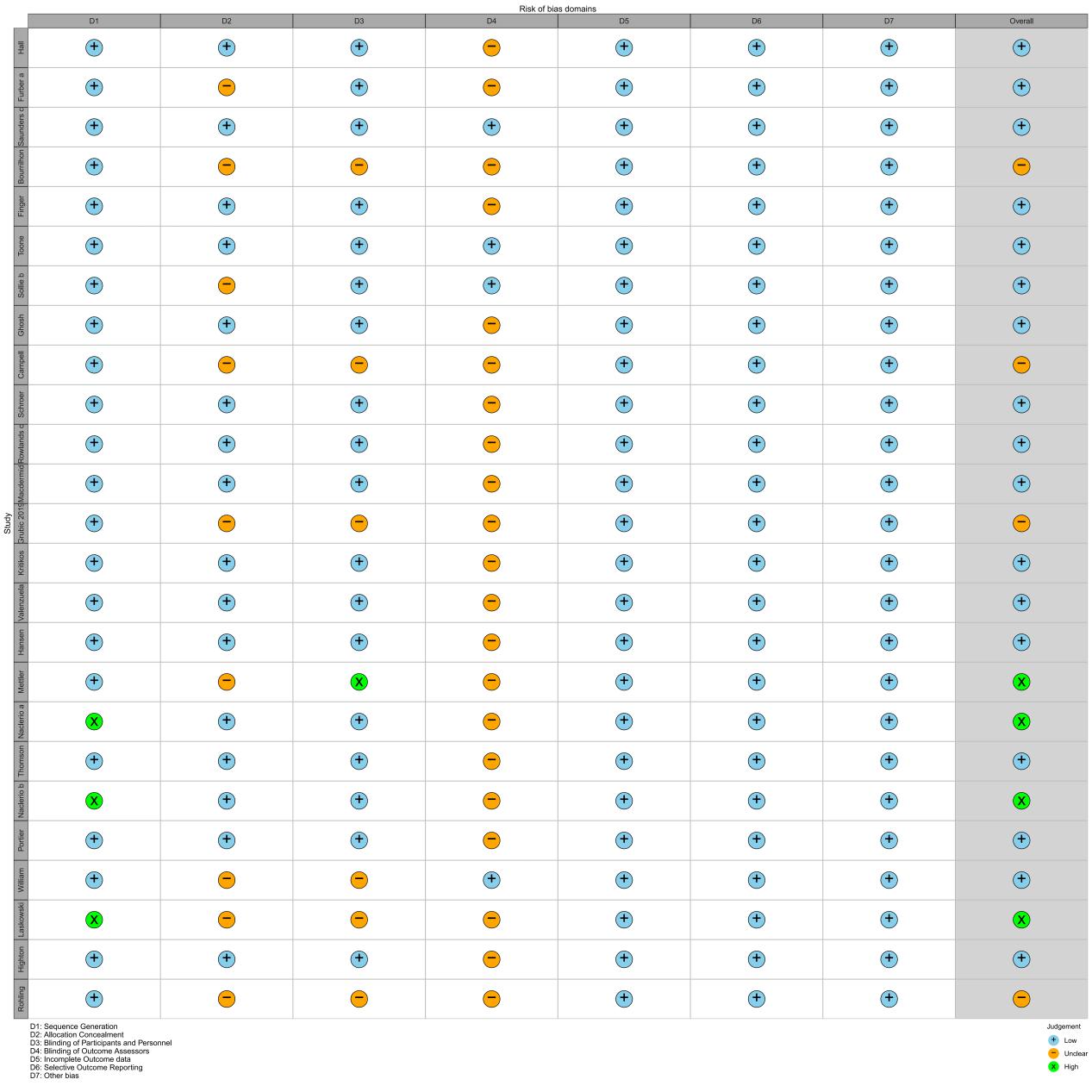


**Fig.S3** Risk of Bias Assessment (c)
