# Supplementary material for: The effectiveness of protein supplements on athletic performance and post-exercise recovery − a Bayesian multilevel meta-analysis of randomized controlled trials
Source: J Int Soc Sports Nutr. 2025 Dec 23;23(1):2605338. doi: 10.1080/15502783.2025.2605338 (PMC12777903; doi:10.1080/15502783.2025.2605338)
Supplement: supplementary material — Supplementary_file_S1. [file RSSN_A_2605338_SM6148.docx]

**Supplementary Table S1: Retrieve Records**

| Web of Science  September 26, 2024 | |
| --- | --- |
|  |  |
| 1 | (((((((((((((((ALL=(plant protein)) OR ALL=(animal protein)) OR ALL=(egg protein)) OR ALL=(beef protein )) OR ALL=(high protein)) OR ALL=(vegetable protein)) OR ALL=(whey protein )) OR ALL=(soy protein )) OR ALL=(pea protein )) OR ALL=(white protein)) OR ALL=(Amino acid)) OR ALL=(rice protein)) OR ALL=(potato protein)) OR ALL=(milk protein)) OR ALL=(wheat protein)) OR ALL=(casein protein) |
|  |  |
|  |  |
|  |  |
|  |  |
|  |  |
| 2 | ((((((((((ALL=(Athletic performance)) OR ALL=(sports performance)) OR ALL=(muscle strength)) OR ALL=(muscle power)) OR ALL=(endurance performance))) OR ALL=(aerobic ability)) OR ALL=(anaerobic ability)) OR ALL=(resistance training)) OR ALL=(lower body strength)) AND ALL=(Athletes) |
|  |  |
|  |  |
|  |  |
|  |  |
|  |  |
| 3 | #2 AND #1 |
|  |  |
|  |  |
| Scopus September 26, 2024 | |
|  |  |
| 1 | plant AND protein OR animal AND protein OR egg AND protein OR beef AND protein OR high AND protein OR vegetable AND protein OR whey AND protein OR soy AND protein OR pea AND protein OR white AND protein OR amino AND acid OR rice AND protein OR potato AND protein OR milk AND protein OR wheat AND protein OR casein AND protein AND athletic AND performance OR sports AND performance OR muscle AND strength OR muscle AND power OR endurance AND performance OR aerobic AND ability OR anaerobic AND ability OR resistance AND training OR lower AND body AND strength AND athletes |
|  |  |
|  |  |
|  |  |
|  |  |
|  |  |

| Pubmed September 26, 2024 | |
| --- | --- |
|  |  |
| 1 | Search: (((((((((((((((plant protein) OR (animal protein)) OR (egg protein)) OR (beef protein)) OR (high protein)) OR (vegetable protein)) OR (whey protein)) OR (soy protein)) OR (pea protein)) OR (white protein)) OR (Amino acid)) OR (rice protein)) OR (potato protein)) OR (milk protein)) OR (wheat protein)) OR (casein protein) |
|  |  |
|  |  |
|  |  |
|  |  |
|  |  |
| 2 | (((((((((Athletic performance) OR (sports performance)) OR (muscle strength)) OR (muscle power)) OR (endurance performance)) OR (aerobic ability)) OR (anaerobic ability)) OR (resistance training)) OR (lower body strength)) AND (Athletes) |
|  |  |
|  |  |
|  |  |
|  |  |
|  |  |
| 3 | Search: ((((((((((((((((plant protein) OR (animal protein)) OR (egg protein)) OR (beef protein)) OR (high  protein)) OR (vegetable protein)) OR (whey protein)) OR (soy protein)) OR (pea protein)) OR (white protein)) OR (Amino acid)) OR (rice protein)) OR (potato protein)) OR (milk protein)) OR (wheat protein)) OR (casein protein)) AND ((((((((((Athletic performance) OR (sports performance)) OR (muscle strength)) OR (muscle power)) OR (endurance performance)) OR (aerobic ability)) OR (anaerobic ability)) OR (resistance training)) OR (lower body strength)) AND (Athletes)) Filters: Randomized Controlled Trial |
|  |  |
|  |  |
| Ovid September 26, 2024 | |
|  |  |
| 1 | (plant protein or animal protein or egg protein or beef protein or high protein or vegetable protein or whey protein or soy protein or pea protein or white protein or Amino acid or rice protein or potato protein or milk protein or wheat protein or casein protein).mp. [mp=title, abstract, full text, caption text] |
|  |  |
|  |  |
|  |  |
|  |  |
|  |  |
| 2 | ((Athletic performance or sports performance or muscle strength or muscle power or endurance performance or aerobic ability or anaerobic ability or resistance training or lower body strength) and athletes).mp. [mp=title, abstract, full text, caption text] |
|  |  |
|  |  |
|  |  |
|  |  |
|  |  |
| 3 | 1 and 2 |
|  |  |
|  |  |

| EBSCO (MEDLINE Complete) September 26, 2024 | |
| --- | --- |
|  |  |
| 1 | plant protein OR animal protein OR egg protein OR beef protein OR high protein OR vegetable protein OR whey protein OR soy protein OR pea protein OR white protein OR rice protein OR potato protein |
|  |  |
|  |  |
|  |  |
|  |  |
|  |  |
| 2 | athletic performance OR sports performance OR muscle strength OR muscle power OR endurance performance OR aerobic abilities OR lower body strength AND athletes |
|  |  |
|  |  |
|  |  |
|  |  |
|  |  |
| 3 | S1 AND S2 |
|  |  |
|  |  |
| EBSCO (CINAHL Complete) September 26, 2024 | |
|  |  |
| 1 | plant protein OR animal protein OR egg protein OR beef protein OR high protein OR vegetable protein OR whey protein OR soy protein OR pea protein OR white protein OR rice protein OR potato protein |
|  |  |
|  |  |
|  |  |
|  |  |
|  |  |
| 2 | milk protein OR wheat protein OR casein protein |
|  |  |
|  |  |
|  |  |
|  |  |
|  |  |
| 3 | S1 OR S2 |
|  |  |
|  |  |
| 4 | athletic performance OR sports performance OR muscle strength OR muscle power OR  endurance performance OR aerobic abilities OR anaerobic abilities OR resistance training OR lower body strength AND athletes |
|  |  |
| 5 | S3 AND S4 |
|  |  |

| EBSCO (SPORTDiscus) September 26, 2024 | |
| --- | --- |
|  |  |
| 1 | plant protein OR animal protein OR egg protein OR beef protein OR high protein OR vegetable protein OR whey protein OR soy protein OR pea protein OR white protein OR rice protein OR potato protein |
|  |  |
|  |  |
|  |  |
|  |  |
|  |  |
| 2 | milk protein OR wheat protein OR casein protein |
|  |  |
|  |  |
|  |  |
|  |  |
|  |  |
| 3 | S1 OR S2 |
|  |  |
|  |  |
| 4 | athletic performance OR sports performance OR muscle strength OR muscle power OR  endurance performance OR aerobic abilities OR anaerobic abilities OR resistance training OR lower body strength AND athletes |
|  |  |
| 5 | S3 AND S4 |
|  |  |
